# Differences across the lifespan between females and males in the top 20 causes of disease burden globally: a systematic analysis of the Global Burden of Disease Study 2021

**DOI:** 10.1016/S2468-2667(24)00053-7

**Published:** 2024-05-01

**Authors:** Vedavati Patwardhan, Gabriela F Gil, Alejandra Arrieta, Jack Cagney, Erin DeGraw, Molly E Herbert, Mariam Khalil, Erin C Mullany, Erin M O’Connell, Cory N Spencer, Caroline Stein, Aiganym Valikhanova, Emmanuela Gakidou, Luisa S Flor

**Affiliations:** a Center on Gender Equity and Health, University of California, San Diego, CA, USA; b Institute for Health Metrics and Evaluation, University of Washington, Seattle, WA, USA; c Department of Health Metrics Sciences, School of Medicine, University of Washington, Seattle, WA, USA

## Abstract

**Background:**

Sex and gender shape health. There is a growing body of evidence focused on comprehensively and systematically examining the magnitude, persistence, and nature of differences in health between females and males. Here, we aimed to quantify differences in the leading causes of disease burden between females and males across ages and geographies.

**Methods:**

We used the Global Burden of Disease Study 2021 to compare disability-adjusted life-year (DALY) rates for females and males for the 20 leading causes of disease burden for individuals older than 10 years at the global level and across seven world regions, between 1990 and 2021. We present absolute and relative differences in the cause-specific DALY rates between females and males.

**Findings:**

Globally, females had a higher burden of morbidity-driven conditions with the largest differences in DALYs for low back pain (with 478·5 [95% uncertainty interval 346·3–632·8] more DALYs per 100 000 individuals among females than males), depressive disorders (348·3 [241·3–471·0]), and headache disorders (332·9 [48·3–731·9]), whereas males had higher DALY rates for mortality-driven conditions with the largest differences in DALYs for COVID-19 (with 1767·8 [1581·1–1943·5] more DALYs per 100 000 among males than females), road injuries (1012·2 [934·1–1092·9]), and ischaemic heart disease (1611·8 [1405·0–1856·3]). The differences between sexes became larger over age and remained consistent over time for all conditions except HIV/AIDS. The largest difference in HIV/AIDS was observed among those aged 25–49 years in sub-Saharan Africa with 1724·8 (918·8–2613·7) more DALYs per 100 000 among females than males.

**Interpretation:**

The notable health differences between females and males point to an urgent need for policies to be based on sex-specific and age-specific data. It is also important to continue promoting gender-sensitive research, and ultimately, implement interventions that not only reduce the burden of disease but also achieve greater health equity.

**Funding:**

Bill & Melinda Gates Foundation.

## Introduction

It is widely recognised that sex and gender interact with factors such as race, ethnicity, socioeconomic status, disability, age, and sexual orientation to shape human health.^1^ Sex refers to the biological factors that are associated with physical and physiological traits, including hormones, sex chromosomes, and reproductive anatomy, whereas gender relates to socially constructed roles, behaviours, and identities of women, men, and gender-diverse people, also influenced by historical and cultural contexts.^2^ These dynamic and intertwined biological and social processes result in men, women, and sex-diverse and gender-diverse individuals experiencing health and disease differently. Existing literature acknowledges, for instance, that women, despite experiencing a higher degree of non-fatal health conditions including chronic conditions, outlive men in almost all settings around the world,^3^ a phenomenon referred to as the male–female health-survival paradox.^4^ In the present analysis, we use available data to explore differences in health between females and males, as data on health outcomes for additional sex categorisations (such as intersex) and gender identities are very sparse.

Globally, funding agencies, peer-reviewed journals, and researchers have implemented policies requiring greater transparency in the reporting of sex and gender in studies, resulting in a growing body of evidence on the magnitude, persistence, and nature of sex and gender health differences and disparities.^5^, ^6^ *The Lancet* Series on gender equality norms and health, for example, explores the ways in which restrictive gender norms can influence all facets of an individual's wellbeing, providing innovative viewpoints and evidence on the effects of gender inequalities and norms on health, along with strategies to address these disparities.^7^, ^8^, ^9^, ^10^ Additionally, studies have also applied a gendered approach to both quantitative and qualitative data analysis for various specific health risks and outcomes, including BMI and obesity,^11^, ^12^ mood disorders and depression,^13^ HIV,^14^, ^15^ and non-communicable diseases.^16^ Identifying where important differences between females and males currently exist, across ages and geographies, is an important first step to enhancing this evidence base and to inform future research and policy making that advances health equity. The biological and social factors that influence health fluctuate with age, making it essential to understand the nature of sex and gender differences in health conditions at each life stage.^17^ As populations age, comprehensive sex-focused and gender-focused analyses that include older age groups have become particularly relevant.^18^

**Research in context**

**Evidence before this study**

In the past two decades, there has been a notable increase in attention on the intersection of sex, gender, and health, resulting in a growing body of evidence on the magnitude, persistence, and nature of sex and gender disparities across several health outcomes. To better understand the existing body of evidence, we searched for articles in English on PubMed and Google Scholar using the search terms “[female or male] health disparities”, “sex and gender influences on health”, “gender disparities in health”, and other cause-specific terms related to leading global causes of disease burden. We did not include any time restrictions to our search and identified articles that discussed the relationship between sex, gender, and different associated health outcomes for females and males. Increasingly, studies have begun elucidating the effects of gender norms on conditions, such as BMI and obesity, mood disorders and depression, HIV, and non-communicable diseases. Researchers have also used Global Burden of Disease (GBD) data within their studies to identify causes of disability-adjusted life-years (DALYs) that most disproportionately affect women and girls or men and boys globally. In some cases, these studies have added a layer of analysis to look more specifically at gender in relation to these health differences, and in one case identified new proxy measures to quantify gender influences on health. However, these studies have not systematically analysed the pattern of disease burden at different age ranges or across different geographies, which presents an opportunity to better understand global and regional trends in health differences across the life course.

**Added value of this study**

This study presents a systematic exploration of health differences between females and males across major causes of disease burden. We used data from GBD 2021 to analyse global and regional patterns in female and male health loss in different age groups and years for the 20 major causes of disease burden. DALY rates of mental, musculoskeletal, and neurological disorders globally were higher for females, whereas DALY rates of COVID-19, road injuries, ischaemic heart disease, stroke, liver disease, and tuberculosis were higher for males. For several conditions, the differences between females and males emerged at an early age and continued to grow over the life course. Additionally, our findings highlight various regional patterns in the distribution of the disease burden across age groups for females and males. Providing similar estimates over conditions, regions, and time enables researchers and policy makers to clearly identify key health differences, and inform priority areas for interventions targeting differences in female–male health outcomes.

**Implications of all the available evidence**

Our research findings reveal substantial global health differences between females and males, with little progress in bridging these health differences between 1990 and 2021. Most conditions that disproportionately affect females or males, such as depressive disorders, anxiety disorders, and road injuries, begin to differentiate in adolescence. Existing research suggests that this period coincides with a crucial age when gender norms and attitudes intensify and puberty reshapes self-perceptions. Together, this evidence highlights the importance of developing gender-sensitive interventions and preventive measures from a young age. The health differences continue to grow with age, creating significant differences in health between females and males across life stages. While the evidence base on these differences is growing, the increasing female to male ratio in ageing populations makes it crucial for researchers and policy makers to recognise that women's health-care needs extend beyond areas that have been prioritised to date, such as reproductive health services. Significant geographical differences in the trends of health differences between females and males were found in our analysis and across other research. These patterns highlight the complex and context-specific relationships between health and gender norms, economic conditions, and social practices, which require more granular analyses to provide deeper contextual insights into the underlying factors driving health differences. Furthermore, our analysis adds to the current global health movement calling for inclusive health data and incorporating information on sex and gender at both the point of data collection and reporting.

In this Article, we aimed to investigate health differences between females and males in the 20 leading global causes of disease burden using the results from the Global Burden of Diseases, Injuries, and Risk Factors Study (GBD) 2021. We report the excess disease burden of females and males, spanning age ranges from adolescence to older ages, at the global level and across seven world regions, from 1990 to 2021. Due to limitations in data availability, our analysis is driven by sex-disaggregated data, reflecting a binary framework (female or male); we have therefore elected to use the terms female and male throughout the text when referring to the findings of our study.

## Methods

### Data

GBD 2021 reported estimates of mortality and morbidity trends from 1990 to 2021 in 204 countries and territories for 371 diseases and injuries. We use these estimates to quantify differences between females and males across the top 20 causes of disability-adjusted life-years (DALYs) globally. The GBD complies with GATHER^19^ (appendix pp 2–4) and is registered and approved through the University of Washington Institutional Review Board (study number 9060).

Our analysis draws upon sex-specific GBD estimates to explore cause-specific DALY rates (per 100 000) for females and males. It is not possible to disentangle the effects of sex and gender on health using GBD estimates alone, owing to limitations inherent in the primary data that underpin our estimates. These data are predominantly reported with a distinction by sex only or frequently conflate the concepts of sex and gender. Although these restrictions exist, here we explore the differences in disease burden for females and males across different life stages, acknowledging that the health outcomes analysed are influenced by both sex and gender.^20^

### Disability-adjusted life-years

Our primary measures of interest are DALYs, which measure the total health loss due to both fatal and non-fatal disease burden and enable comparison across diseases and injuries.^21^ DALYs are calculated as the sum of years of life lost (YLLs) due to premature mortality and years of life lived with disability (YLDs). YLLs are derived as the number of deaths per 100 000 people multiplied by a global standard life expectancy at the age of death. YLDs are estimated as the combined prevalence and duration of a disease or injury weighted by a measure of disease severity (referred to as disability weights) that range from 0 (full health) to 1 (fatal severity). As the sum of YLLs and YLDs, one DALY represents the loss of 1 year of healthy life in the population. Details regarding the estimation of DALYs, YLLs, and YLDs, including methods to assess the relative morbidity and mortality from individual diseases and injuries, as well as the disability weights, have been published elsewhere.^22^

### Geographical units, cause levels, and time periods

GBD 2021 DALY estimates are available for females and males for 20 standard age bands for 204 countries and territories from early childhood to older than 95 years. These locations are grouped into seven super-regions according to income and epidemiological profile: central Europe, eastern Europe, and central Asia; high-income countries; Latin America and the Caribbean; North Africa and the Middle East; south Asia; southeast Asia, east Asia, and Oceania; and sub-Saharan Africa. The present analysis focuses on health patterns globally and for each of the seven super-regions as an overarching overview of differences between females and males.

Diseases and injuries in GBD 2021 are organised within a four-level hierarchical structure of cause categories, ranging from the broadest groupings at Level 1 to the most detailed at Level 4 (see appendix pp 5–12 for the full GBD cause hierarchy). Within our current analysis, we have focused on Level 3 causes as they constitute our primary outcomes of interest, ensuring consistency with the GBD's conventional reporting standard. The 175 Level 3 causes of interest were ranked globally according to the overall age-standardised rate of associated DALYs per 100 000 individuals older than 10 years in 2021. We focused our analysis on the top 20 causes according to this ranking, highlighting the differences in disease burden between females and males for causes with the greatest disease burden. Although this approach excludes important sex-specific causes, including some of the top causes of disease burden among females (see appendix pp 13–20 for the full ranking for females and males), it allows us to assess the health differences between females and males among key outcomes where comparisons are feasible. We present results for 2021, the most recent year with available GBD estimates, with comparisons over time from 1990.

### Analysis

We did descriptive analyses of differences in cause-specific DALY rates using R version 4.0.5 Patched (2021-04-30 r80288). We report findings for age groups corresponding to extended adolescence (aged 10–24 years), young adults (aged 25–49 years), middle-aged adults (aged 50–69 years), older adults (aged 70 years and older), and age-standardised (aged 10 years and older). Children younger than 10 years are not included due to the unique disease profile among young children. Of our five age groups, age group-specific DALY rates are available with no further processing for two GBD standard age groups: 50–69 years and 70 years and older. For the age groups 10–24 years and 25–49 years, we aggregated standard 5-year age group DALY rates using GBD age-specific, sex-specific, and super-region-specific population estimates. We used the GBD world population age standard weights (appendix p 12) to estimate age-standardised DALY rates among those aged 10 years and older.^3^ The age-standardised DALY rates facilitate comparisons across geography by accounting for differences in the population-level age distributions.

We analysed both the absolute and relative differences in DALY rates between females and males by cause, globally and across super-regions. The absolute differences between females and males were calculated as the age, cause, super-region, and year-specific female DALY rate minus the corresponding age, cause, super-region, and year-specific male DALY rate. A positive value indicated a higher rate for females than for males. The relative difference between females and males was determined as a percent difference, calculated as the age, cause, super-region, and year-specific absolute difference divided by the corresponding male rate of DALYs and multiplied by 100. A positive value implies higher relative rates among females than males. We also did a comparison of differences in DALY rates between 1990 and 2021 by subtracting the group-specific absolute difference in 2021 from that in 1990. We further examined the relative contribution of premature mortality (YLLs) versus morbidity (YLDs) to the disease burden for females and males, separately, by deriving age, cause, super-region, and year-specific ratios of YLD rates to YLL rates. Each of the metrics reported in this study, including the absolute and relative differences between females and males and change in difference over time, were calculated at the draw-level with 500 draws from the posterior distribution. We present 95% uncertainty intervals (UIs) for each based on the 2·5th and 97·5th percentile of the draws. These UIs reflect the degree of uncertainty surrounding the estimates presented and correspond to a range that is likely to include the true value in question. We evaluated the existence of an observed absolute or relative difference between females and males by whether the 95% UIs derived from the posterior distribution for the differences include zero (the absence of a difference).

### Role of the funding source

The funders of this study were not involved in the study design, data collection, data analysis, data interpretation, the writing of the report, or the decision to submit.

## Results

### Global and super-region patterns

**Figure 1:**
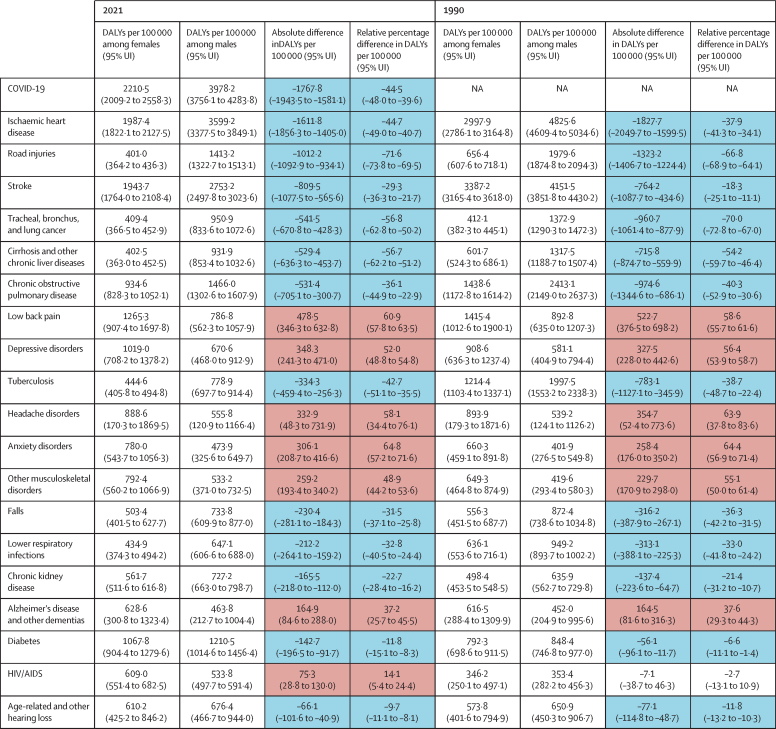
Global DALY rates (per 100 000 population) and 95% UIs for females and males and their absolute and relative differences in 1990 and 2021, age-standardised (10 years and older) The health outcomes presented here reflect the top 20 causes of disease burden, measured by DALYs, observed across females and males for the age group of 10 years and older globally in 2021. Health conditions are ranked by the magnitude of the absolute gap in DALYs per 100 000 regardless of the direction of the gap. The absolute differences between females and males were calculated as the DALY rate for females minus the rate for males for each specific cause and year, with a positive value indicating a higher rate for females than for males. The relative gap was computed as a relative percent difference, with a positive value indicating higher values among females relative to males. Cell colours denote whether the absolute and relative gaps in DALY rates indicate that the cause disproportionately affects females (red) or males (blue). DALYs=disability-adjusted life-years. UI=uncertainty interval. NA=not available.

**Figure 2:**
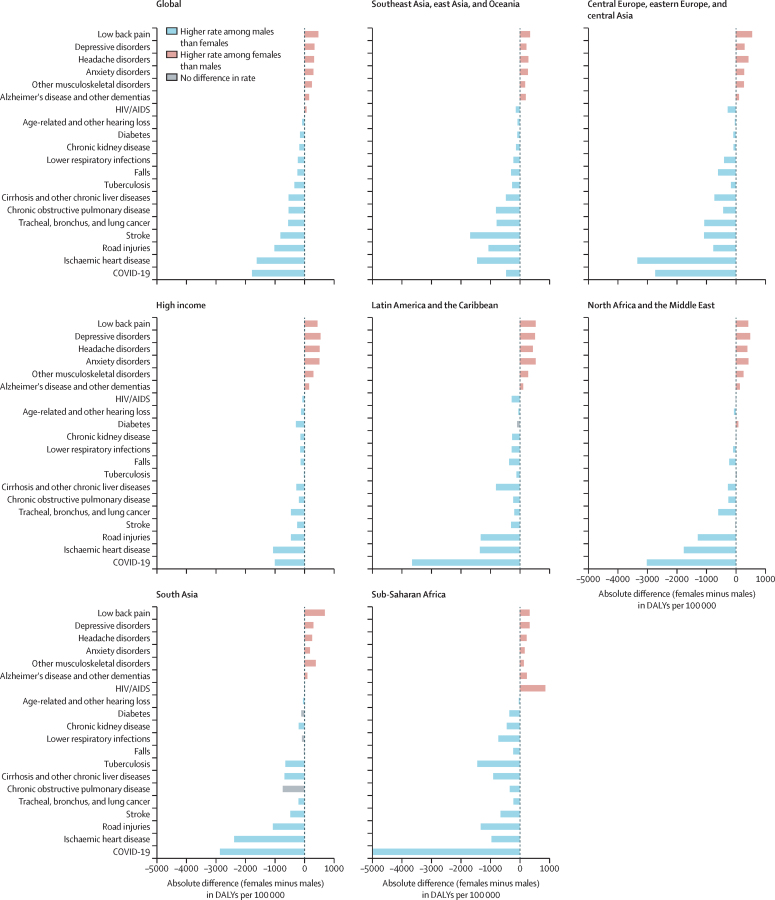
Global and regional absolute difference in DALY rates (per 100 000 population) between females and males, age-standardised (10 years and older), 2021 The causes of disease burden represented on the y-axis are the top 20 causes of DALYs observed across females and males for the age group of 10 years and older globally in 2021. Health conditions are ranked based on the difference in age-standardised DALY rates (per 100 000 population) between females and males observed at the global level. The absolute differences between females and males were calculated as the DALY rate for females minus the rate for males for each specific cause and geography. A positive value indicates a higher rate for females than for males. The direction of the differences is also represented through colour coding. Grey bars reflect causes in which the 95% uncertainty interval of the absolute difference includes zero, reflecting no observed difference between females and males. DALY=disability-adjusted life-year.

**Figure 3:**
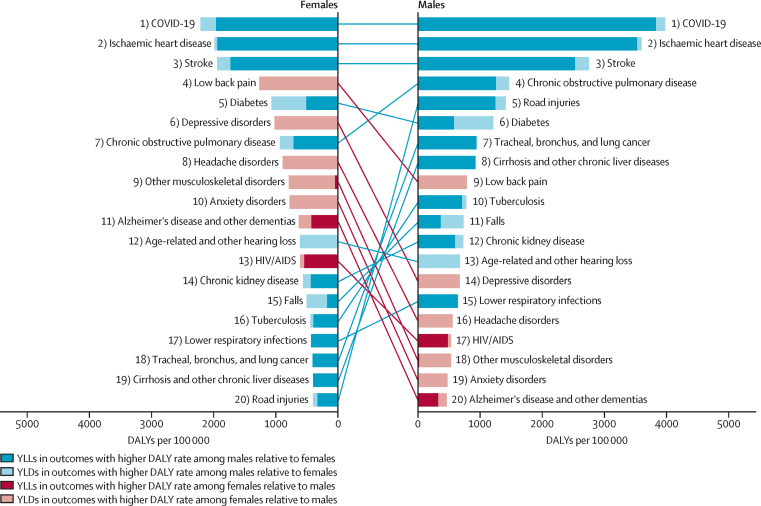
Global rankings of the top 20 causes of DALYs globally for females and males, age-standardised (10 years and older), 2021 The list of causes of disease burden represents the top 20 causes of age-standardised DALYs observed across females and males for the age group of 10 years and older globally in 2021. This same list of health conditions was ranked according to the DALY rates (per 100 000 population) for both females and males globally in 2021 for the same age group. The colours of the bars and lines denote whether DALY rates are higher for females (red) or males (blue) as established by whether the 95% uncertainty interval of the absolute difference in DALY rates includes zero. The degree of transparency reflects the composition of DALYs for each cause, split between mortality (YLL) and morbidity (YLD). DALY=disability-adjusted life-year. YLL=years of life lost. YLD=years lived with disability.

The age-standardised (aged 10 years and older) DALY rates (per 100 000 population) for both females and males in 2021 and 1990 globally are displayed in figure 1. DALY rates were higher for males than females for 13 of the 20 causes. The seven causes with higher DALY rates for females than males are low back pain, depressive disorders, headache disorders, anxiety disorders, other musculoskeletal disorders, dementia, and HIV/AIDS (figures 1, 2). Most of these health conditions ranked within the top ten major causes of disease burden for females in 2021 (appendix pp 13–20). Furthermore, morbidity (measured using YLDs) constituted much of the burden for causes with higher DALY rates among females, whereas premature mortality (measured as YLLs) formed a larger share of the causes with higher rates among males (figure 3).

The largest absolute difference disfavouring females globally was observed for low back pain with an estimated 478·5 (95% UI 346·3–632·8) more DALYs per 100 000 among females than among males in 2021 (figure 1). We found particularly pronounced differences for low back pain and other musculoskeletal disorders in south Asia (693·7 [497·2–925·2] and 388·8 [282·1–517·9] more DALYs per 100 000 among females, respectively) and in central Europe, eastern Europe, and central Asia (low back pain: 550·8 [398·2–726·5] and other musculoskeletal disorders: 277·9 [199·7–372·5]; figure 2; appendix pp 21–28). The higher burden of mental disorders faced by females compared with males was also observed across all regions (figures 1, 2). Specifically, global DALY rates for depressive disorders were 1019·0 (708·2–1378·2) for females and 670·6 (468·0–912·9) for males, marking it the condition with the second-largest absolute difference that disproportionately affects females (figure 1). Regionally, this difference was most pronounced in countries in the high-income region (553·7 [392·0–750·7] more DALYs per 100 000 among females), closely followed by Latin America and the Caribbean (515·5 [342·6–718·1]) and north Africa and the Middle East (491·9 [324·2–703·2]; figure 2; appendix pp 21–28). A similar pattern was noted for anxiety disorders, with the greatest absolute differences observed in Latin America and the Caribbean (538·9 [360·7–739·5] more DALYs per 100 000 for females than males), high-income region (512·2 [347·4–706·7]), and north Africa and the Middle East (427·8 [275·5–610·5]; figure 2; appendix pp 21–28). When focusing on relative differences, anxiety disorders emerged as the primary cause of excess burden for females, with global rates for females being 64·8% (95% UI 57·2%–71·6%) higher than those for males (figure 1; appendix p 69). Both the relative and absolute burden of headache disorders, other musculoskeletal disorders, and dementia were higher for females across all world regions (figure 2). Geographical patterns indicate that the global absolute difference in HIV/AIDS rates is primarily driven by sub-Saharan Africa (figure 2; appendix pp 21–28) where females have a higher DALY rate than males. North Africa and the Middle East also stand out from global patterns with higher rates among females than males for diabetes, which otherwise had higher rates among males across four other super-regions and globally (Figure 1, Figure 2; appendix pp 21–28).

Of the 20 causes of interest, 13 showed higher age-standardised global DALY rates among males compared with females (figure 2). Given its overall magnitude, COVID-19 had the largest absolute difference between females and males globally in 2021 with 1767·8 (95% UI 1581·1–1943·5) more DALYs per 100 000 among males than among females (figure 1). COVID-19 disproportionately affected men in all regions, with the widest differences observed in sub-Saharan Africa (4994·2 [4524·2–5378·3] more DALYs per 100 000 among males compared with females), Latin American and the Caribbean (3651·3 [2821·5–4448·3]), and north Africa and the Middle East (3009·6 [2570·4–3381·6]; figure 2; appendix pp 21–28). Next was ischaemic heart disease (figure 1). The region with the largest absolute difference in the burden of ischaemic heart disease was central Europe, eastern Europe, and central Asia (figure 2), where the male DALY rate was higher by 3333·2 (2803·5–3898·3) DALYs per 100 000 than the female rate (appendix pp 21–28). Although COVID-19 and ischaemic heart disease had the largest absolute differences between females and males, they did not have the greatest relative differences between females and males. When looking at the relative differences, road injuries show the largest differences, particularly in Latin America and the Caribbean and in south Asia (appendix p 69). Lung cancer and cirrhosis ranked second and third in terms of conditions with the largest relative difference disfavouring males globally (appendix p 69).

### Patterns by age

**Figure 4:**
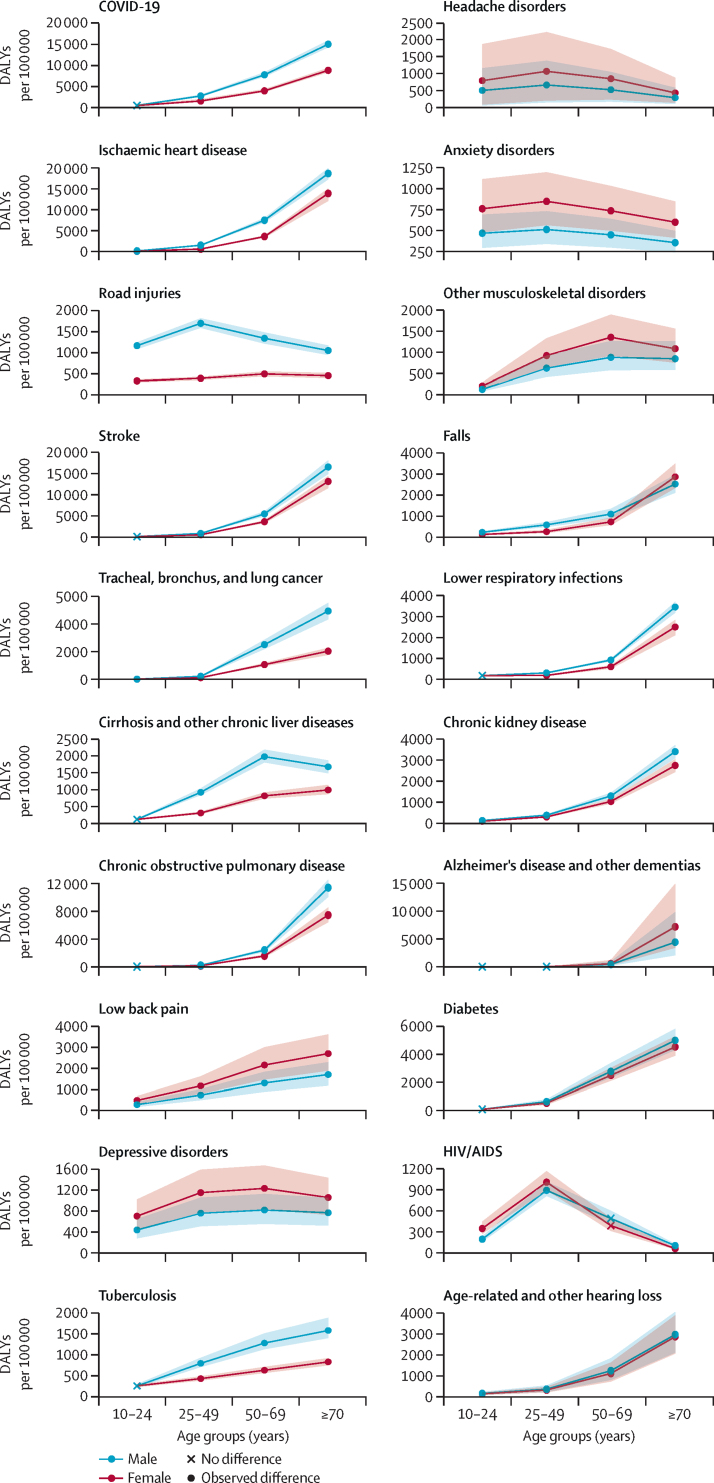
Global DALY rates (per 100 000 population) and 95% UIs for females and males across age groups, 2021 The figure displays a list of the top 20 causes of disease burden, represented by age-standardised DALYs, observed across females and males for the age group of 10 years and older globally in 2021. Health conditions are ordered according to the magnitude of the overall age-standardised absolute difference in DALYs per 100 000 in 2021 between females and males. The red colour corresponds to rates among females, the blue colour corresponds to rates among males, and 95% UIs for each estimate are shown as a shaded coloured area around each point estimate. The shape of the points reflects age groups in which the 95% UI of the absolute difference includes zero, reflecting no observed difference between females and males for the given cause. DALY=disability-adjusted life-year. UI=uncertainty interval.

For conditions in which global female DALY rates were higher, differences between females and males began early in life (figure 4; appendix pp 29–33). The disparity in mental, neurological, and musculoskeletal disorders disfavouring females aged 10–24 years intensified globally among those aged 25–49 years (figure 4; appendix pp 71–72). Across all regions and conditions, excluding COVID-19, the largest absolute difference noted among those aged 25–49 years was for HIV/AIDS in sub-Saharan Africa (1724·8 [918·8–2613·7] more DALYs per 100 000 among females than males; appendix pp 34–67). The disparity between female and male rates of low back pain and other musculoskeletal disorder DALYs continued to widen at ages 50–69 years, whereas there was a modest reduction in the female–male difference in DALYs due to anxiety, depressive, and headache disorders (figure 4; appendix p 72). Finally, the difference in low back pain further increased in the oldest age group, with Alzheimer's and other types of dementia emerging as leading conditions of excess disease burden among females (figure 4; appendix p 73). Although the disease burden due to falls disproportionately affected males for most of their lives, this trend reversed in later ages (figure 4; appendix p 73). Relative differences for the different age groups are in the appendix (pp 74–77).

In contrast, the differences in ischaemic heart disease, lung cancer, and chronic kidney disease, of which males had a higher burden, were small at early ages (10–24 years) but widened consistently over the course of life (figure 4; appendix p 70). Other differences in DALY rates disadvantaging males emerged for COVID-19, tuberculosis, cirrhosis and other liver diseases, and chronic obstructive pulmonary disease (COPD) between ages 25 and 49 years and continued to increase at ages 50–69 years (figure 4; appendix pp 71–72). Road injuries, however, presented an exception to this global pattern. From ages 10 to 24 years, males bore a higher burden of global DALY rates in all regions, making it the most notable cause of female–male differences that disadvantaged males in this age group (appendix p 70). This difference continued to widen at ages 25–49 years, when males had 1301·8 (1202·7–1405·6) more DALYs due to road injuries than females globally, but diminished in older ages (figure 4; appendix pp 71–73).

### Global trends over time

**Figure 5:**
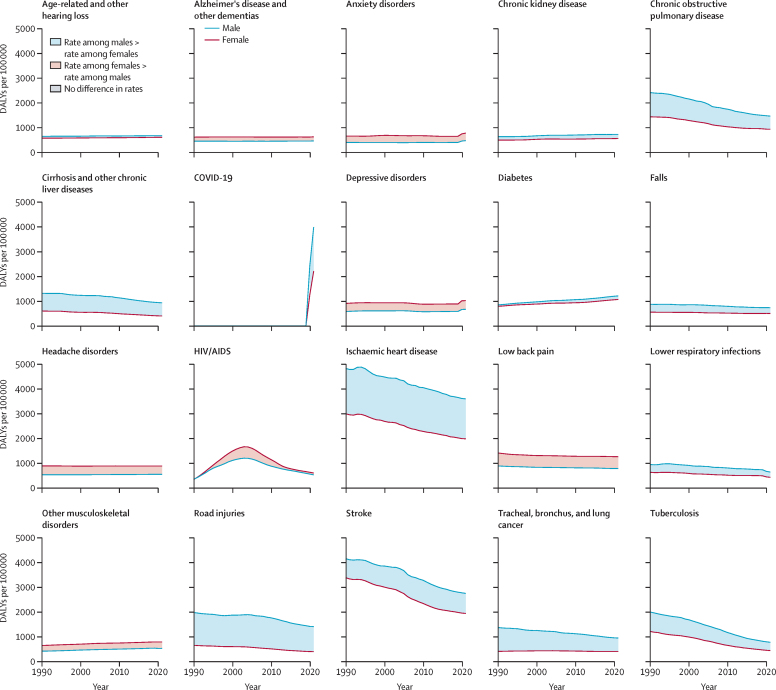
Temporal pattern of global absolute difference between females and males in DALY rates (per 100 000 population) between 1990 and 2021, age-standardised (10 years and older) The figure displays the time trends in age-standardised DALYs per 100 000 for the top 20 causes of age-standardised disease burden observed for females and males aged 10 years and above globally in 2021. The red coloured line corresponds to the mean estimate of rates among females and the blue coloured line corresponds to the mean estimate of rates among males. The distance between the lines reflects the absolute difference between females and males, whereas the colour shading of this space corresponds to whether females had a higher rate of DALYs than males (red) or vice versa (blue). Grey shading, as seen in the early years of HIV/AIDS, indicates that the 95% uncertainty interval of the absolute difference between females and males includes zero and suggests no difference in rate. DALY=disability-adjusted life-year.

With some exceptions, we observed gradual changes over time in the absolute differences between female and male age-standardised rates of DALYs (figure 5). For depressive disorders, anxiety disorders, other musculoskeletal disorders, and diabetes, the differences between female and male rates grew slowly between 1990 and 2021 (figure 5; appendix p 78). Although these four conditions had an increase in the difference disfavouring females, diabetes had an increase in the absolute difference from 56·1 (11·7–96·1) more DALYs per 100 000 among males than females in 1990 to 142·7 (91·7–196·5) in 2021 (figure 1). Similarly gradual changes in differences were evident in the eight conditions where the absolute difference between females and males decreased, including for COPD and tracheal, bronchus, and lung cancer (figure 5). However, some of this apparent progress was largely driven by changes in the overall disease burden, with the relative difference between females and males being higher for low back pain, road injuries, and ischaemic heart disease in 2021 than in 1990 (figure 1; appendix p 79). Furthermore, we observed no changes in the absolute difference between age-standardised DALY rates among females and males for Alzheimer's disease and other dementias, chronic kidney disease, tuberculosis, cirrhosis and other liver diseases, stroke, and ischaemic heart disease between 1990 and 2021 (appendix p 78).

Although the global pattern in the difference between age-standardised DALY rates for females and for males was stable over time for most causes, HIV/AIDS showed substantial temporal variation. For HIV/AIDS, the difference between females and males increased between 1990 and 2021 from no observed difference to disadvantaging females (figure 1). This shift followed a hike in the global age-standardised DALY rate of HIV/AIDS among females from 1990 through to the early 2000s that was not matched by an increase observed in DALY rates among males (figure 5). The global peak was largely driven by the HIV/AIDS crisis in sub-Saharan Africa, where we observed the greatest absolute difference in 2003 at 5768·5 (4750·6–7025·2) more DALYs per 100 000 among females than males (appendix p 80). In other super-regions, we did not observe similar temporal patterns between 1990 and 2021 (appendix pp 81–86), including in the high-income region where there were 393·3 (384·3–405·1) more DALYs per 100 000 among males than females in 1990 (appendix p 81). Sub-Saharan Africa remains the only region where the DALY rate for HIV/AIDS remains higher for females than for men in 2021. A few other causes saw some less drastic geographical variations away from otherwise consistent patterns of disparity over time, including an increasing absolute difference in ischaemic heart disease-related DALYs per 100 000 between females and males in south Asia and in southeast Asia, east Asia, and Oceania (appendix pp 85–86).

## Discussion

Drawing upon GBD 2021 estimates, we identified persistent health differences between females and males across the top 20 causes of disease burden over the last three decades. In 2021, overall, males faced a higher disease burden than females. For 13 of the top 20 causes, including COVID-19, road injuries, and a range of cardiovascular, respiratory, and liver diseases, the DALY rates for males were higher than for females. Females showed higher age-standardised DALY rates associated with low back pain, depressive disorders, headache disorders, anxiety, other musculoskeletal disorders, Alzheimer's disease and other dementias, and HIV/AIDS than males. These differences highlight the importance of taking into consideration the different health needs of females and males across the life course.

Historically, the focus on women's health has been largely focused on sexual and reproductive concerns, which, although crucial, do not encompass the full spectrum of health issues affecting females throughout the life course.^23^ Our analysis highlights, for example, the disproportionate toll of morbidity-driven conditions among females, with the greatest difference observed for mental disorders and musculoskeletal disorders. Non-communicable diseases more commonly experienced by females remain deprioritised in research funding,^24^ scientific literature,^25^ and, most notably, in health systems planning, both in terms of trained professionals and funding allocated to these conditions, despite the health and economic burden associated with them.^26^, ^27^, ^28^ For example, WHO's 2020 Mental Health Atlas highlights the global shortage of health-care workers trained in mental health, especially in low-income and middle-income countries (LMICs), where the rate of mental health workers can be as low as two per 100 000 population, in contrast to over 60 in high-income countries. Furthermore, the report reveals that, on a global scale, only 2·1% of government health expenditure is on mental health, which is particularly concerning given the massive burden associated with mental health disorders and the fact that they disproportionately affect females in all world regions.^29^

Notably, the health differences we have identified begin at a young age—a life stage marked by pubertal changes and intensified gender socialisation, when gender identity, roles, and norms sharply diverge and gain prominence^30^—underscoring the need for early targeted responses. Additionally, the differences between females and males continue to grow over age for many conditions, leaving females with higher degrees of morbidity throughout their lifetime, which is on average longer than for males. Ageing populations across the world place increased demand on already strained health-care systems, calling for increased funding and stronger infrastructure to support the changing needs of their populations.^31^ The higher degree of morbidity among females paired with the increasing ratio of females to males in ageing populations^32^ are crucial components for policy makers to consider as they prepare their health systems for the next decades. This is particularly important for LMICs where the transition in the age structure of their populations is happening alongside persistent challenges from infectious diseases.^33^

Our findings also spotlight the significant health challenges faced by males that necessitate the development of specific strategies and national health plans.^34^ Among these challenges are enduring health issues that lead to premature mortality, notably cardiovascular diseases, cancers, and road injuries, as well as recent and evolving health threats such as the COVID-19 pandemic. Globally, new initiatives have started to be rolled out to develop health strategies for men, including in the 2018 Strategy on the Health and Well-Being of Men in the WHO European region, which was ratified by 53 member countries. However, to date, only seven countries have designated national-level policies to address men's health.^35^, ^36^ Furthermore, just as with the differences disadvantaging females, health differences affecting males, such as road injuries and ischaemic heart disease, often have their roots early in life, highlighting the importance of interventions and preventive measures that get implemented from a young age. Such initiatives need to recognise the different health-seeking patterns of males and should, among other strategies, engage in cross-sectoral actions, including working with the social and education sectors to promote positive and healthy gender norms and roles at all levels of education, to address behavioural risks that are globally more common among males, such as smoking and alcohol use.^35^, ^37^, ^38^ These behaviours typically begin during adolescence and continue into adulthood, creating long-term exposure pathways associated with adverse health outcomes including lower respiratory infections, COPD, lung cancer, cardiovascular conditions, and cirrhosis and other liver diseases.^39^, ^40^ The disproportionate toll of road injuries on males highlights another area in which intensified preventive measures across transportation, safety, health, and social systems are urgently needed to mitigate the risks of traffic accidents and their consequent mortality and morbidity among young adult males.^41^

The persistent differences observed across various health conditions between females and males underscore the paramount importance of sex-specific and gender identity-specific data. This is one of the important components of the larger toolbox for crafting sex-responsive and gender-responsive policies and programmatic actions. Despite repeated commitments, including the Sustainable Development Goals’ specific target (17.18) advocating for sex-disaggregated health data,^42^ the requirements for sex and gender analysis from certain funding agencies,^6^ and the adoption of the Sex and Gender Equity in Research guidelines by several peer-reviewed journals,^5^ there remain persistent and substantial differences in the availability of sex-disaggregated data in peer-reviewed publications^43^ and administrative data systems.^44^ Even for COVID-19, a condition that dominated global attention over the past 4 years, and where marked differences in outcomes between females and males have been noted—we found 44·5% lower COVID-19 burden among females than males, reflecting the largest absolute difference across all conditions globally—approximately 60% of countries did not consistently report sex-disaggregated data on both cases and deaths.^45^, ^46^ Information on gender identity is even more scarce, and is often partially or entirely absent from population-based surveys that collect data on health and gender-related indicators,^47^ despite recent efforts advocating for more gender-inclusive data.^48^ This is an additional indication of the need for enhanced data availability to effectively monitor, understand, and address the health needs of various population groups.

Although disaggregating data by sex and gender identity is crucial, it constitutes only the initial step in unravelling the roots of health disparities between men, women, and gender-diverse individuals.^49^ To effectively tailor responses that accommodate the life experiences and health challenges of all and promote changes, it is imperative to continue innovating in the analysis of secondary health data from a gender perspective.^8^, ^50^ This involves acknowledging gender as a social construct and focusing on the power dynamics and societal norms, which are key drivers of health disparities.^10^ This should be done in parallel with in-depth qualitative research to construct health equity strategies that are sensitive to and take into consideration the intricate relationship between gender, health, and other social determinants.^51^ An approach that encompasses several geographical and social contexts is crucial for pinpointing and addressing the particular health needs of diverse populations. For example, our analysis of HIV/AIDS indicated that aside from sub-Saharan Africa, where females faced significantly higher burden, males were disproportionately affected in all other regions. This pattern highlights the need for targeted policies and planning to address the specific risk behaviours, social dynamics, and access to health-care services of females and males in various parts of the world. Without granular and intersectional insights, the systemic barriers that sustain health inequities will remain unchallenged.

Our study's findings must be considered within the context of its limitations. First, this research inherits the general limitations of the GBD, which are extensively described elsewhere.^22^ For instance, although the GBD strives to adjust for sources of bias inherent in self-reported data, such as recall bias and desirability bias, as well as non-sampling errors and differential diagnostic patterns, we recognise that the GBD methods are not able to entirely address and correct for all biases in reported data or fully control for differences in diagnostic likelihood for specific conditions. For example, the under-diagnosis and treatment of depression in males^52^ and of cardiovascular diseases in females^53^ have been previously discussed. Additionally, the effect of imbalanced or incomplete representation of population groups, such as the under-representation of men and some age groups in topic-specific surveys, can skew the accuracy of conclusions drawn regarding the health needs of all people.^47^, ^54^ Therefore, systematic biases present in epidemiological data might affect the apparent magnitude of the health differences described in our study. To partially explore the significance of this limitation, we ran the same analysis on the 2019 DALY estimates from WHO and found substantial agreement with our key results.^55^ Second, our final estimations follow sex disaggregation in the GBD, which adheres to a binary framework (female or male), thereby preventing the production of estimates for gender-diverse or sex-diverse groups and limiting our analysis. Since health differences are often exacerbated for sex-diverse and gender-diverse individuals, there is a need for data spanning the gender spectrum to support more inclusive health research.^56^ Third, GBD data alone cannot be used to disentangle the influences of sex and gender on health outcomes and the data also have limited capacity for supporting an intersectional analysis of how sex and gender, as a social construct, interact with other determinants of health, including, but not limited to, race, ethnicity, social class, and sexual orientation. Finally, by examining the top 20 global causes of disease burden for females and males combined, our study does not encompass female-specific and male-specific conditions, such as gynaecological diseases or prostate cancer, nor does it address those conditions that, while affecting both females and males, are particularly relevant exclusively to females or males (eg, dietary iron deficiency for females; self-harm for males; appendix pp 13–20). Similarly, our analysis might not fully represent conditions that are especially pertinent to some age groups or geographical regions. The standardisation of analysed conditions was a necessary step to facilitate cross-population comparisons and to highlight areas where sex-responsive and gender-responsive interventions could yield substantial health benefits. However, it is important to acknowledge that this approach does not explicitly consider variations in health burdens that are specific to sex, age, or location, and that further research is needed for more nuanced priority-setting.

To conclude, our analysis using GBD 2021 estimates showed persistent health differences between females and males from 1990 to 2021. We notably highlight the diverse and evolving health needs of females and males across the globe and through various life stages, including a higher burden of morbidity-related conditions in females and a disproportionate burden of COVID-19 and road injuries in males. These findings underscore the importance of adopting a life course approach in strategic planning for health systems. This approach should encompass early interventions tailored towards preventing the onset and exacerbation of specific health conditions throughout the life course, along with investments, particularly in LMICs, to strengthen and expand services for conditions predominantly affecting individuals older than 50 years, including mental health and musculoskeletal disorders. Effective design and implementation of health system strategies requires an understanding of the complex interplay between sex and gender, and other social determinants of health. As the global population ages, progress towards an equitable and healthy future for all can only be achieved through concerted, sex-informed and gender-informed strategies that recognise the distinct health challenges faced by men and women at different stages of life.

### Contributors

#### Data sharing

The estimates and results of the present analyses are in figure 1 and throughout the appendix. To download the data used in these analyses, please visit the Global Health Data Exchange GBD 2021 website at https://ghdx.healthdata.org/gbd-2021. The code used for generating these analyses is available at https://github.com/ihmeuw/gem.

## Declaration of interests

We declare no competing interests.
