# Supplementary material for: Differences across the lifespan between females and males in the top 20 causes of disease burden globally: a systematic analysis of the Global Burden of Disease Study 2021
Source: Lancet Public Health. 2024 May 1;9(5):e282–94. doi: 10.1016/S2468-2667(24)00053-7 (PMC11080072; doi:10.1016/S2468-2667(24)00053-7)

THE LANCET
Public Health

Supplementary appendix

This appendix formed part of the original submission and has been peer reviewed. We post it as supplied by the authors.

Supplement to: Patwardhan V, Gil GF, Arrieta A, et al. Differences across the lifespan between females and males in the top 20 causes of disease burden globally: a systematic analysis of the Global Burden of Disease Study 2021. *Lancet Public Health* 2024; **9:** e282–94.

Supplementary appendix to “Differences across the Lifespan between Female and Males in the Top 20 Causes of Disease Burden Globally: A Systematic Analysis of the Global Burden of Disease Study 2021”

This appendix provides further methodological detail and supplemental results.

Table of Contents

Section S1. Global Burden of Disease Study materials 2

Table S1. GATHER Checklist 2

Table S2. GBD Cause Hierarchy 5

Table S3. World population age-standard GBD weights for standard five-year age groups above 10 years old 12

Section S2. Supplementary Results 13

Table S4. DALYs per 100,000 individuals over 10+ years of age globally in 2021 by cause for all level 3 hierarchy GBD causes and ranking for females and males 13

Table S5. Age-standardised (10 years and older) Disability-Adjusted Life Year (DALY) rates (per 100,000 population) and the absolute and relative gaps between females and males for the top 20 global causes by year and super-region 21

Table S6. Global Disability-Adjusted Life Year (DALY) rates (per 100,000 population) for females and males by age and year and the absolute and relative gaps between females and males for the top 20 global causes 29

Table S7. Location-specific Disability-Adjusted Life Year (DALY) rates (per 100,000 population) for females and males and the absolute and relative gaps between females and males for the top 20 global causes by year, age, and super-region 34

Section S3. Supplementary Figures 68

Figure S1. Global and regional relative difference in Disability-Adjusted Life Year (DALY) rates (per 100,000 population) between females and males, age-standardised (10 years and older), 2021 69

Figure S2. Global and regional absolute difference in Disability-Adjusted Life Year (DALY) rates (per 100,000 population) between females and males, 10-24 years old, 2021 70

Figure S3. Global and regional absolute difference in Disability-Adjusted Life Year (DALY) rates (per 100,000 population) between females and males, 25-49 years old, 2021 71

Figure S4. Global and regional absolute difference in Disability-Adjusted Life Year (DALY) rates (per 100,000 population) between females and males, 50-69 years old, 2021 72

Figure S5. Global and regional absolute difference in Disability-Adjusted Life Year (DALY) rates (per 100,000 population) between females and males, 70+ years old, 2021 73

Figure S6. Global and regional relative difference in Disability-Adjusted Life Year (DALY) rates (per 100,000 population) between females and males, 10-24 years old, 2021 74

Figure S7. Global and regional relative difference in Disability-Adjusted Life Year (DALY) rates (per 100,000 population) between females and males, 25-49 years old, 2021 ... 75

Figure S8. Global and regional relative difference in Disability-Adjusted Life Year (DALY) rates (per 100,000 population) between females and males, 50-69 years old 2021 ... 76

Figure S9. Global and regional relative difference in Disability-Adjusted Life Year (DALY) rates (per 100,000 population) between females and males, 70+ years old, 2021 ... 77

Figure S10. Change in global absolute difference in Disability-Adjusted Life Year (DALY) rates (per 100,000 population) among females and males from 1990 to 2021, age-standardised (10 years and older) ... 78

Figure S11. Change in global relative difference in Disability-Adjusted Life Year (DALY) rates (per 100,000 population) among females and males from 1990 to 2021, age-standardised (10 years and older) ... 79

Figure S12. Temporal pattern of absolute difference between females and males in Disability-Adjusted Life Year (DALY) rates (per 100,000 population) between 1990 and 2021 in sub-Saharan Africa, age-standardised (10 years and older) ... 80

Figure S13. Temporal pattern of absolute difference between females and males in Disability-Adjusted Life Year (DALY) rates (per 100,000 population) between 1990 and 2021 in high-income countries, age-standardised (10 years and older) ... 81

Figure S14. Temporal pattern of absolute difference between females and males in Disability-Adjusted Life Year (DALY) rates (per 100,000 population) between 1990 and 2021 in Central Europe, Eastern Europe, and Central Asia, age-standardised (10 years and older) ... 82

Figure S15. Temporal pattern of absolute difference between females and males in Disability-Adjusted Life Year (DALY) rates (per 100,000 population) between 1990 and 2021 in Latin America and Caribbean, age-standardised (10 years and older) ... 83

Figure S16. Temporal pattern of absolute difference between females and males in Disability-Adjusted Life Year (DALY) rates (per 100,000 population) between 1990 and 2021 in North Africa and Middle East, age-standardised (10 years and older) ... 84

Figure S17. Temporal pattern of absolute difference between females and males in Disability-Adjusted Life Year (DALY) rates (per 100,000 population) between 1990 and 2021 in South Asia, age-standardised (10 years and older) ... 85

Figure S18. Temporal pattern of absolute difference between females and males in Disability-Adjusted Life Year (DALY) rates (per 100,000 population) between 1990 and 2021 in Southeast Asia, East Asia, and Oceania, age-standardised (10 years and older) ... 86

Section S1. Global Burden of Disease Study materials

Table S1. GATHER Checklist

Checklist of information that should be included in new reports of global health estimates

Item #	Checklist item	Reported on page #

**Objectives and funding**		
**1**	Define the indicator(s), populations (including age, sex, and geographic entities), and time period(s) for which estimates were made.	Main text: Methods – "Data", "Geographical units, cause levels, and time periods", and "Analysis", Pages 6-8
**2**	List the funding sources for the work.	Main text: Acknowledgments<br>Main text: Methods, "Role of the funding source", Page 8
**Data Inputs**		
For all data inputs from multiple sources that are synthesized as part of the study:		
**3**	Describe how the data were identified and how the data were accessed.	Main text: Methods, "Data", Page 5-6
**4**	Specify the inclusion and exclusion criteria. Identify all ad-hoc exclusions.	Main text: Methods, "Geographical units, cause levels, and time periods", Page 7<br>Cause estimates were eligible for analysis based on their ranking of overall associated age-standardized 10+ DALYs per 100,000 individuals in 2021.
**5**	Provide information on all included data sources and their main characteristics. For each data source used, report reference information or contact name/institution, population represented, data collection method, year(s) of data collection, sex and age range, diagnostic criteria or measurement method, and sample size, as relevant.	Main text: Methods, "Data", Page 5-6<br>Main text: Methods, "Geographical units, cause levels, and time periods", Page 7
**6**	Identify and describe any categories of input data that have potentially important biases (e.g., based on characteristics listed in item 5).	Main text: Methods, "Data" and "Disability-adjusted life years (DALYs)", Page 5-6<br>Main text: Discussion, Page 15-16
For data inputs that contribute to the analysis but were not synthesized as part of the study:		
**7**	Describe and give sources for any other data inputs.	Not applicable
For all data inputs:		
**8**	Provide all data inputs in a file format from which data can be efficiently extracted (e.g., a spreadsheet rather than a PDF), including all relevant meta-data listed in item 5. For any data inputs that cannot be shared because of ethical or legal reasons, such as third-party ownership, provide a contact name or the name of the institution that retains the right to the data.	Data inputs used in this analysis are available for download in excel format at https://ghdx.healthdata.org/gbd-2021 . [This link is not yet live but will be upon publication].
**Data analysis**		
**9**	Provide a conceptual overview of the data analysis method. A diagram may be helpful.	Main text: Methods, "Data" and "Disability-adjusted life years (DALYs)", Page 5-6<br>Main text: Methods, "Analysis", Page 7-8
**10**	Provide a detailed description of all steps of the analysis, including mathematical formulae. This description should cover, as relevant, data cleaning, data pre-processing, data adjustments and weighting of data sources, and mathematical or statistical model(s).	Main text: Methods; "Analysis", Page 7-8
**11**	Describe how candidate models were evaluated and how the final model(s) were selected.	Not applicable as the present study reports on a descriptive analysis.

**12**	Provide the results of an evaluation of model performance, if done, as well as the results of any relevant sensitivity analysis.	Not applicable as the present study reports on a descriptive analysis.
**13**	Describe methods for calculating uncertainty of the estimates. State which sources of uncertainty were, and were not, accounted for in the uncertainty analysis.	Main text: Methods, "Data" and "Analysis, Pages 5-8<br>More details on the uncertainty for the input estimates can be found in the GBD 2021 Capstone publications cited. Descriptive metrics were calculated with 500 draws to iterate out uncertainty.
**14**	State how analytic or statistical source code used to generate estimates can be accessed.	Code used to produce the descriptive measures and statistical tests for the present analysis can be found at https://github.com/ihmeuw/gem [The code will be made available upon publication].
**Results and Discussion**		
**15**	Provide published estimates in a file format from which data can be efficiently extracted.	The published estimates will be made available in an excel format at [The link will be made available upon resubmission]. They are also available in Appendix Section S2.
**16**	Report a quantitative measure of the uncertainty of the estimates (e.g. uncertainty intervals).	95% uncertainty intervals are provided for all mean estimates in the main text and appendix.<br>Main text: Results, Page 9-12<br>Table 1<br>Appendix: Section S2
**17**	Interpret results in light of existing evidence. If updating a previous set of estimates, describe the reasons for changes in estimates.	Main text: Discussion, Page 12-15
**18**	Discuss limitations of the estimates. Include a discussion of any modelling assumptions or data limitations that affect interpretation of the estimates.	Main text: Discussion, Page 15-17<br>Main text: Methods, "Data", Page 5-6

This checklist should be used in conjunction with the GATHER statement and Explanation and Elaboration document, found on gather-statement.org

Table S2. GBD Cause Hierarchy

Level 1 causes	Level 2 causes	Level 3 causes	Level 4 causes
Communicable, maternal, neonatal, and nutritional diseases	Neglected tropical diseases and malaria	Malaria	
		Chagas disease	
		Leishmaniasis	Visceral leishmaniasis
			Cutaneous and mucocutaneous leishmaniasis
		African trypanosomiasis	
		Schistosomiasis	
		Cysticercosis	
		Cystic echinococcosis	
		Lymphatic filariasis	
		Onchocerciasis	
		Trachoma	
		Dengue	
		Yellow fever	
		Rabies	
		Intestinal nematode infections	Ascariasis
			Trichuriasis
			Hookworm disease
		Food-borne trematodiases	
		Other neglected tropical diseases	
		Leprosy	
		Ebola	
		Zika virus	
		Guinea worm disease	
	Nutritional deficiencies	Protein-energy malnutrition	
		Iodine deficiency	
		Vitamin A deficiency	
		Dietary iron deficiency	
		Other nutritional deficiencies	
	HIV/AIDS and sexually transmitted infections	HIV/AIDS	HIV/AIDS - Drug-susceptible Tuberculosis
			HIV/AIDS - Multidrug-resistant Tuberculosis without extensive drug resistance
			HIV/AIDS - Extensively drug-resistant Tuberculosis
			HIV/AIDS resulting in other diseases
		Sexually transmitted infections excluding HIV	Syphilis
			Chlamydial infection
			Gonococcal infection
			Trichomoniasis
			Genital herpes
			Other sexually transmitted infections
		Tuberculosis	Latent tuberculosis infection

	Respiratory infections and tuberculosis		Drug-susceptible tuberculosis
			Multidrug-resistant tuberculosis without extensive drug resistance
			Extensively drug-resistant tuberculosis
		Lower respiratory infections	
		Upper respiratory infections	
		Otitis media	
		COVID-19	
	Enteric infections	Diarrheal diseases	
		Other intestinal infectious diseases	
		Typhoid and paratyphoid	Typhoid fever
			Paratyphoid fever
		Invasive Non-typhoidal Salmonella (iNTS)	
	Other infectious diseases	Meningitis	
		Encephalitis	
		Diphtheria	
		Pertussis	
		Tetanus	
		Measles	
		Varicella and herpes zoster	
		Acute hepatitis	Acute hepatitis A
			Acute hepatitis B
			Acute hepatitis C
			Acute hepatitis E
		Other unspecified infectious diseases	
	Maternal and neonatal disorders	Maternal disorders	Maternal hemorrhage
			Maternal sepsis and other maternal infections
			Maternal hypertensive disorders
			Maternal obstructed labor and uterine rupture
			Maternal abortion and miscarriage
			Ectopic pregnancy
			Indirect maternal deaths
			Late maternal deaths
			Maternal deaths aggravated by HIV/AIDS
			Other direct maternal disorders
		Neonatal disorders	Neonatal preterm birth
			Neonatal encephalopathy due to birth asphyxia and trauma
			Neonatal sepsis and other neonatal infections
			Hemolytic disease and other neonatal jaundice
			Other neonatal disorders
Non-communicable disea	Neoplasms	Esophageal cancer	
		Stomach cancer	

		Liver cancer	Liver cancer due to hepatitis B
			Liver cancer due to hepatitis C
			Liver cancer due to alcohol use
			Liver cancer due to NASH
			Hepatoblastoma
			Liver cancer due to other causes
		Larynx cancer	
		Tracheal, bronchus, and lung cancer	
		Breast cancer	
		Cervical cancer	
		Uterine cancer	
		Prostate cancer	
		Colon and rectum cancer	
		Lip and oral cavity cancer	
		Nasopharynx cancer	
		Other pharynx cancer	
		Gallbladder and biliary tract cancer	
		Pancreatic cancer	
		Malignant skin melanoma	
		Non-melanoma skin cancer	Non-melanoma skin cancer (squamous-cell carcinoma)
			Non-melanoma skin cancer (basal-cell carcinoma)
		Ovarian cancer	
		Testicular cancer	
		Kidney cancer	
		Bladder cancer	
		Brain and central nervous system cancer	
		Thyroid cancer	
		Mesothelioma	
		Hodgkin lymphoma	
		Non-Hodgkin lymphoma	Burkitt lymphoma
			Other non-Hodgkin lymphoma
		Multiple myeloma	
		Leukemia	Acute lymphoid leukemia
			Chronic lymphoid leukemia
			Acute myeloid leukemia
			Chronic myeloid leukemia
			Other leukemia
		Other malignant neoplasms	
		Other neoplasms	Myelodysplastic, myeloproliferative, and other hematopoietic neoplasms
			Benign and in situ intestinal neoplasms
			Benign and in situ cervical and uterine neoplasms

			Other benign and in situ neoplasms
		Eye cancer	Retinoblastoma
			Other eye cancers
		Soft tissue and other extraosseous sarcomas	
		Malignant neoplasm of bone and articular cartilage	
		Neuroblastoma and other peripheral nervous cell tumors	
	Cardiovascular diseases	Rheumatic heart disease	
		Ischemic heart disease	
		Stroke	Ischemic stroke
			Intracerebral hemorrhage
			Subarachnoid hemorrhage
		Hypertensive heart disease	
		Cardiomyopathy and myocarditis	Myocarditis
			Alcoholic cardiomyopathy
			Other cardiomyopathy
		Atrial fibrillation and flutter	
		Aortic aneurysm	
		Lower extremity peripheral arterial disease	
		Endocarditis	
		Non-rheumatic valvular heart disease	Non-rheumatic calcific aortic valve disease
			Non-rheumatic degenerative mitral valve disease
			Other non-rheumatic valve diseases
		Other cardiovascular and circulatory diseases	
		Pulmonary Arterial Hypertension	
	Chronic respiratory diseases	Chronic obstructive pulmonary disease	
		Pneumoconiosis	Silicosis
			Asbestosis
			Coal workers pneumoconiosis
			Other pneumoconiosis
		Asthma	
		Interstitial lung disease and pulmonary sarcoidosis	
		Other chronic respiratory diseases	
	Digestive diseases	Cirrhosis and other chronic liver diseases	Chronic hepatitis B including cirrhosis
			Chronic hepatitis C including cirrhosis
			Cirrhosis due to alcohol
			Nonalcoholic fatty liver disease including cirrhosis
			Cirrhosis due to other causes
		Appendicitis	
		Paralytic ileus and intestinal obstruction	
		Inguinal, femoral, and abdominal hernia	
		Inflammatory bowel disease	
		Vascular intestinal disorders	

		Gallbladder and biliary diseases	
		Pancreatitis	
		Other digestive diseases	
		Upper digestive system diseases	Peptic ulcer disease
			Gastritis and duodenitis
			Gastroesophageal reflux disease
	Neurological disorders	Alzheimer's disease and other dementias	
		Parkinson's disease	
		Idiopathic epilepsy	
		Multiple sclerosis	
		Motor neuron disease	
		Other neurological disorders	
		Headache disorders	Migraine
			Tension-type headache
	Mental disorders	Schizophrenia	
		Depressive disorders	Major depressive disorder
			Dysthymia
		Bipolar disorder	
		Anxiety disorders	
		Eating disorders	Anorexia nervosa
			Bulimia nervosa
		Autism spectrum disorders	
		Attention-deficit/hyperactivity disorder	
		Conduct disorder	
		Idiopathic developmental intellectual disability	
		Other mental disorders	
	Musculoskeletal disorders	Rheumatoid arthritis	
		Osteoarthritis	Osteoarthritis hip
			Osteoarthritis knee
			Osteoarthritis hand
			Osteoarthritis other
		Low back pain	
		Neck pain	
		Gout	
		Other musculoskeletal disorders	
	Other non-communicable diseases	Urinary diseases and male infertility	Urinary tract infections and interstitial nephritis
			Urolithiasis
			Benign prostatic hyperplasia
			Male infertility
			Other urinary diseases
		Gynecological diseases	Uterine fibroids
			Polycystic ovarian syndrome

			Female infertility
			Endometriosis
			Genital prolapse
			Premenstrual syndrome
			Other gynecological diseases
		Hemoglobinopathies and hemolytic anemias	Thalassemias
			Thalassemias trait
			Sickle cell disorders
			Sickle cell trait
			G6PD deficiency
			G6PD trait
			Other hemoglobinopathies and hemolytic anemias
		Endocrine, metabolic, blood, and immune disorders	
		Congenital birth defects	Neural tube defects
			Congenital heart anomalies
			Orofacial clefts
			Down syndrome
			Turner syndrome
			Klinefelter syndrome
			Other chromosomal abnormalities
			Congenital musculoskeletal and limb anomalies
			Urogenital congenital anomalies
			Digestive congenital anomalies
			Other congenital birth defects
		Oral disorders	Caries of deciduous teeth
			Caries of permanent teeth
			Periodontal diseases
			Edentulism
			Other oral disorders
		Sudden infant death syndrome	
	Skin and subcutaneous diseases	Dermatitis	Atopic dermatitis
			Contact dermatitis
			Seborrhoeic dermatitis
		Psoriasis	
		Scabies	
		Fungal skin diseases	
		Viral skin diseases	
		Acne vulgaris	
		Alopecia areata	
		Pruritus	
		Urticaria	
		Decubitus ulcer	

		Other skin and subcutaneous diseases	
		Bacterial skin diseases	Cellulitis
			Pyoderma
	Sense organ diseases	Age-related and other hearing loss	
		Other sense organ diseases	
		Blindness and vision loss	Glaucoma
			Cataract
			Age-related macular degeneration
			Refraction disorders
			Near vision loss
			Other vision loss
	Substance use disorders	Alcohol use disorders	
		Drug use disorders	Opioid use disorders
			Cocaine use disorders
			Amphetamine use disorders
			Cannabis use disorders
			Other drug use disorders
	Diabetes and kidney diseases	Diabetes mellitus	Diabetes mellitus type 1
			Diabetes mellitus type 2
		Acute glomerulonephritis	
		Chronic kidney disease	Chronic kidney disease due to diabetes mellitus type 1
			Chronic kidney disease due to diabetes mellitus type 2
			Chronic kidney disease due to hypertension
			Chronic kidney disease due to glomerulonephritis
			Chronic kidney disease due to other and unspecified causes
Injuries	Transport injuries	Road injuries	Pedestrian road injuries
			Cyclist road injuries
			Motorcyclist road injuries
			Motor vehicle road injuries
			Other road injuries
		Other transport injuries	
	Unintentional injuries	Falls	
		Drowning	
		Fire, heat, and hot substances	
		Poisonings	Poisoning by carbon monoxide
			Poisoning by other means
		Exposure to mechanical forces	Unintentional firearm injuries
			Other exposure to mechanical forces
		Adverse effects of medical treatment	
		Animal contact	Venomous animal contact
			Non-venomous animal contact

		Foreign body	Pulmonary aspiration and foreign body in airway
			Foreign body in eyes
			Foreign body in other body part
		Exposure to forces of nature	
		Environmental heat and cold exposure	
		Other unintentional injuries	
	Self-harm and interpersonal violence	Self-harm	Self-harm by firearm
			Self-harm by other specified means
		Interpersonal violence	Physical violence by firearm
			Physical violence by sharp object
			Sexual violence
			Physical violence by other means
		Police conflict and executions	
		Conflict and terrorism	

Table S3. World population age-standard GBD weights for standard five-year age groups above 10 years old

**5-year age group**	**Age-standard weight**
10 to 14 years old	0.089936
15 to 19 years old	0.082891
20 to 24 years old	0.078012
25 to 29 years old	0.075914
30 to 34 years old	0.073217
35 to 39 years old	0.068281
40 to 44 years old	0.061474
45 to 49 years old	0.055113
50 to 54 years old	0.049131
55 to 59 years old	0.043459
60 to 64 years old	0.036822
65 to 69 years old	0.029851
70 to 74 years old	0.022653
75 to 79 years old	0.015976
80 to 84 years old	0.010973
85 to 89 years old	0.006045
90 to 94 years old	0.002467
95+ years old	0.000785

Section S2. Supplementary Results

Table S4. DALYs per 100,000 individuals over 10+ years of age globally in 2021 by cause for all level 3 hierarchy GBD causes and ranking for females and males

Cause	Overall 10+ age-standardized DALYs per 100 000 (95% UI)	Overall cause ranking	Cause ranking among females	Cause ranking among males
COVID-19	3051·4 (2852·2-3373·2)	1	1	1
Ischemic heart disease	2754·4 (2580·8-2900·8)	2	2	2
Stroke	2324·4 (2143·3-2486·3)	3	3	3
Chronic obstructive pulmonary disease	1171·3 (1085·1-1262·7)	4	7	4
Diabetes mellitus	1135·7 (961·3-1359·7)	5	5	6
Low back pain	1030·7 (738·2-1380·5)	6	4	9
Road injuries	908·3 (850·5-972·0)	7	24	5
Depressive disorders	845·2 (589·4-1146·7)	8	6	15
Headache disorders	721·5 (145·5-1513·1)	9	8	18
Other musculoskeletal disorders	663·7 (466·1-901·1)	10	10	20
Tracheal, bronchus, and lung cancer	663·7 (597·2-730·8)	11	22	7
Cirrhosis and other chronic liver diseases	663·0 (613·7-724·5)	12	23	8
Age-related and other hearing loss	642·3 (445·4-893·8)	13	13	14
Chronic kidney disease	638·2 (586·0-694·6)	14	16	12
Anxiety disorders	626·9 (434·1-846·6)	15	11	21
Falls	620·4 (512·8-750·3)	16	18	11
Tuberculosis	607·4 (551·7-674·9)	17	19	10
HIV/AIDS	570·3 (526·3-632·4)	18	14	19
Alzheimer's disease and other dementias	561·5 (265·2-1191·5)	19	12	22
Lower respiratory infections	531·3 (488·5-573·4)	20	21	16
Self-harm	510·7 (478·8-545·2)	21	31	13
Gynecological diseases	420·8 (294·6-586·8)	22	9	170

Blindness and vision loss	418·2 (273·8-614·6)	23	20	26
Interpersonal violence	393·4 (370·5-418·7)	24	44	17
Diarrheal diseases	384·4 (266·4-563·5)	25	26	27
Hypertensive heart disease	375·6 (318·2-412·3)	26	27	28
Colon and rectum cancer	352·7 (326·4-377·5)	27	34	24
Dietary iron deficiency	345·9 (232·9-492·3)	28	17	55
Oral disorders	332·4 (198·1-501·9)	29	30	29
Stomach cancer	327·2 (282·4-372·2)	30	40	23
Osteoarthritis	304·5 (145·8-614·1)	31	28	36
Neck pain	300·5 (201·6-425·3)	32	29	37
Breast cancer	297·7 (279·2-317·6)	33	15	142
Asthma	270·9 (217·0-336·5)	34	33	35
Neonatal disorders	254·3 (186·0-319·2)	35	35	32
Alcohol use disorders	251·9 (200·5-315·1)	36	61	25
Drug use disorders	237·8 (194·4-277·3)	37	42	31
Upper digestive system diseases	221·5 (169·8-302·9)	38	38	39
Schizophrenia	221·4 (163·8-284·9)	39	39	40
Malaria	218·5 (80·0-466·0)	40	41	38
Rheumatic heart disease	194·2 (166·8-227·9)	41	37	49
Maternal disorders	193·7 (166·9-226·9)	42	25	170
Esophageal cancer	185·0 (164·5-207·9)	43	65	30
Liver cancer	181·9 (164·4-203·7)	44	57	34
Idiopathic epilepsy	176·8 (136·9-222·5)	45	45	46
Endocrine, metabolic, blood, and immune disorders	171·8 (130·2-232·0)	46	36	59
Pancreatic cancer	162·3 (149·9-174·9)	47	47	47
Urinary diseases and male infertility	159·2 (139·1-184·1)	48	54	44

Cardiomyopathy and myocarditis	155·4 (143·3-168·6)	49	60	42
Cervical cancer	143·3 (131·2-155·9)	50	32	170
Exposure to mechanical forces	143·0 (113·5-179·0)	51	71	41
Autism spectrum disorders	142·8 (97·0-201·8)	52	63	48
Drowning	142·3 (132·1-151·4)	53	73	43
Leukemia	139·9 (118·8-155·2)	54	52	51
Congenital birth defects	133·7 (110·1-164·2)	55	48	57
Other cardiovascular and circulatory diseases	133·3 (111·0-162·3)	56	51	53
Other mental disorders	132·7 (84·9-200·1)	57	55	52
Conflict and terrorism	126·9 (105·1-158·4)	58	90	45
Atrial fibrillation and flutter	126·3 (105·7-152·4)	59	53	56
Hemoglobinopathies and hemolytic anemias	123·5 (93·8-161·3)	60	43	74
Bipolar disorder	121·2 (78·6-174·3)	61	50	62
Other unintentional injuries	120·0 (91·6-147·6)	62	74	50
Prostate cancer	119·5 (105·4-128·8)	63	173	33
Brain and central nervous system cancer	116·1 (99·9-134·8)	64	58	58
Gallbladder and biliary diseases	112·3 (85·3-149·3)	65	49	65
Parkinson's disease	111·6 (100·6-121·4)	66	64	54
Non-Hodgkin lymphoma	105·9 (97·9-115·7)	67	66	60
Fire, heat, and hot substances	100·7 (85·2-119·9)	68	56	67
Dermatitis	87·6 (54·3-133·1)	69	59	75
Other malignant neoplasms	85·3 (76·2-92·8)	70	67	68
Lip and oral cavity cancer	84·3 (76·3-91·1)	71	81	61
Meningitis	82·7 (74·1-95·7)	72	68	69
Acne vulgaris	81·5 (51·1-128·2)	73	62	77

Paralytic ileus and intestinal obstruction	81·3 (71·2-91·4)	74	72	66
Ovarian cancer	74·1 (67·2-80·5)	75	46	170
Conduct disorder	68·5 (36·5-110·0)	76	86	70
Bladder cancer	64·2 (59·1-70·2)	77	106	63
Other transport injuries	64·2 (58·8-70·6)	78	110	64
Scabies	64·0 (35·6-104·4)	79	75	79
Pancreatitis	60·0 (53·3-68·9)	80	102	71
Interstitial lung disease and pulmonary sarcoidosis	59·0 (51·2-65·8)	81	89	76
Animal contact	57·1 (47·7-67·6)	82	84	82
Other neurological disorders	55·8 (46·7-66·6)	83	80	84
Kidney cancer	54·3 (51·2-57·1)	84	103	73
Gallbladder and biliary tract cancer	53·8 (45·3-62·5)	85	79	89
Eating disorders	53·8 (32·6-85·0)	86	70	104
Upper respiratory infections	52·6 (31·7-77·3)	87	82	88
Protein-energy malnutrition	52·5 (45·7-62·0)	88	93	83
Psoriasis	52·3 (38·1-69·7)	89	83	86
Other sense organ diseases	51·7 (31·4-77·7)	90	78	92
Non-rheumatic valvular heart disease	49·5 (44·6-54·9)	91	95	85
Other unspecified infectious diseases	48·3 (35·8-61·5)	92	77	106
Other skin and subcutaneous diseases	48·1 (24·6-86·6)	93	87	93
Typhoid and paratyphoid	47·9 (23·3-84·4)	94	92	90
Adverse effects of medical treatment	47·0 (41·0-53·3)	95	88	95
Foreign body	47·0 (40·6-54·2)	96	105	81
Viral skin diseases	46·4 (29·7-68·8)	97	96	91
Aortic aneurysm	45·5 (41·8-49·0)	98	107	80

Idiopathic developmental intellectual disability	44·9 (20·4-76·8)	99	91	102
Rheumatoid arthritis	44·7 (33·5-57·9)	100	76	115
Larynx cancer	44·6 (41·5-48·0)	101	140	72
Fungal skin diseases	42·7 (17·3-88·5)	102	99	98
Encephalitis	42·7 (37·4-49·0)	103	98	99
Urticaria	42·3 (27·8-59·7)	104	85	109
Acute hepatitis	41·5 (34·9-50·5)	105	101	94
Other digestive diseases	40·6 (35·8-46·0)	106	100	100
Other pharynx cancer	40·3 (37·3-43·5)	107	133	78
Multiple myeloma	37·4 (32·8-41·4)	108	104	97
Uterine cancer	36·6 (32·5-40·6)	109	69	170
Malignant neoplasm of bone and articular cartilage	36·6 (30·0-41·4)	110	108	96
Nasopharynx cancer	35·8 (31·7-39·9)	111	123	87
Poisonings	33·8 (25·4-37·9)	112	115	101
Iodine deficiency	33·5 (17·6-60·2)	113	97	120
Other neglected tropical diseases	31·2 (22·6-42·1)	114	94	135
Other chronic respiratory diseases	30·6 (25·8-36·3)	115	111	108
Endocarditis	28·9 (25·6-32·3)	116	117	107
Bacterial skin diseases	27·8 (24·9-31·7)	117	116	111
Inguinal, femoral, and abdominal hernia	27·6 (22·7-33·6)	118	130	105
Otitis media	27·1 (15·9-44·3)	119	113	113
Schistosomiasis	25·6 (14·8-44·0)	120	112	116
Gout	25·2 (17·1-35·8)	121	141	103
Vascular intestinal disorders	24·9 (23·0-26·8)	122	114	117
Malignant skin melanoma	24·5 (21·5-26·7)	123	121	112

Environmental heat and cold exposure	23·2 (17·1-28·1)	124	136	110
Lower extremity peripheral arterial disease	23·2 (18·9-30·2)	125	119	118
Soft tissue and other extraosseous sarcomas	22·1 (19·0-26·9)	126	122	119
Inflammatory bowel disease	22·0 (19·1-25·5)	127	118	123
Sexually transmitted infections excluding HIV	21·7 (14·3-33·1)	128	109	137
Dengue	20·0 (9·3-32·1)	129	125	125
Appendicitis	18·4 (15·9-21·6)	130	127	129
Thyroid cancer	18·1 (16·0-20·0)	131	120	138
Onchocerciasis	18·1 (10·8-27·3)	132	131	127
Lymphatic filariasis	18·0 (10·5-30·8)	133	146	114
Cysticercosis	18·0 (11·4-26·4)	134	124	134
Non-melanoma skin cancer	17·8 (15·9-19·7)	135	137	121
Other neoplasms	17·7 (15·2-21·0)	136	134	122
Other nutritional deficiencies	17·5 (14·9-20·9)	137	126	136
Invasive Non-typhoidal Salmonella (iNTS)	17·1 (8·8-28·9)	138	132	131
Hodgkin lymphoma	17·1 (12·3-22·1)	139	135	126
Food-borne trematodiases	14·5 (8·3-23·9)	140	144	130
Motor neuron disease	14·2 (13·2-15·4)	141	143	133
Multiple sclerosis	14·2 (12·2-16·5)	142	129	145
Police conflict and executions	13·9 (11·9-17·3)	143	154	124
Attention-deficit/hyperactivity disorder	13·5 (7·6-21·8)	144	148	128
Exposure to forces of nature	13·1 (10·5-16·4)	145	128	148
Decubitus ulcer	12·0 (9·2-13·3)	146	142	141
Intestinal nematode infections	11·8 (7·0-18·6)	147	139	143

Pruritus	11·7 (5·7-21·4)	148	138	144
Mesothelioma	10·0 (9·2-10·7)	149	153	139
Testicular cancer	8·6 (8·1-9·2)	150	173	132
Alopecia areata	8·3 (5·4-11·7)	151	145	152
Leishmaniasis	8·1 (5·0-15·3)	152	149	146
Tetanus	7·6 (3·3-12·1)	153	151	147
Pulmonary Arterial Hypertension	7·5 (6·3-8·5)	154	147	150
Pneumoconiosis	6·5 (5·7-7·5)	155	165	140
Varicella and herpes zoster	6·3 (5·0-8·1)	156	150	151
Rabies	5·8 (3·6-8·0)	157	155	149
Vitamin A deficiency	5·5 (3·6-7·9)	158	152	153
Acute glomerulonephritis	4·1 (2·8-5·2)	159	156	154
Chagas disease	3·5 (3·0-3·9)	160	160	156
Eye cancer	3·3 (2·5-4·1)	161	158	157
Yellow fever	3·3 (1·2-7·0)	162	162	155
Measles	3·1 (1·8-5·2)	163	157	158
Pertussis	2·9 (1·2-6·0)	164	159	159
Neuroblastoma and other peripheral nervous cell tumors	1·9 (1·6-2·1)	165	163	160
Trachoma	1·8 (1·2-2·5)	166	161	161
Cystic echinococcosis	1·3 (1·0-1·6)	167	164	162
African trypanosomiasis	0·9 (0·3-2·3)	168	166	163
Diphtheria	0·5 (0·4-0·6)	169	168	164
Other intestinal infectious diseases	0·4 (0·3-0·5)	170	167	165
Leprosy	0·3 (0·2-0·5)	171	169	166
Ebola	0·0 (0·0-0·0)	172	170	167
Zika virus	0·0 (0·0-0·0)	173	171	168

Guinea worm disease	0·0 (0·0-0·0)	174	172	169
Sudden infant death syndrome	0·0 (0·0-0·0)	175	173	170

The health outcomes colored in green here reflect the top 20 causes of disease burden, measured by Disability-Adjusted Life Years (DALYs), observed across females and males for the age group of 10 years and older globally in 2021. Health conditions are ranked by the age-standardized (10 years and older) health burden in DALYs per 100,000 individuals across both females and males.

Table S5. Age-standardised (10 years and older) Disability-Adjusted Life Year (DALY) rates (per 100,000 population) and the absolute and relative gaps between females and males for the top 20 global causes by year and super-region

Location	Cause	2021				1990			
		**DALYs per 100,000 among Females (95% UI)**	**DALYs per 100,000 among Males (95% UI)**	**Absolute gap in DALYs per 100,000**	**Relative % gap in DALYs per 100,000**	**DALYs per 100,000 among Females (95% UI)**	**DALYs per 100,000 among Males (95% UI)**	**Absolute gap in DALYs per 100,000**	**Relative % gap in DALYs per 100,000**
Central Europe, Eastern Europe, and Central Asia	Age-related and other hearing loss	551·9 (384·8–768·6)	583·9 (404·2–810·2)	-32·0 (-55·1–-13·5)	-5·4 (-7·5–-2·8)	557·2 (389·6–773·9)	589·3 (412·0–814·6)	-32·1 (-53·5–-14·0)	-5·4 (-7·3–-2·9)
	Alzheimer's disease and other dementias	522·5 (249·5–1102·1)	415·1 (194·7–899·8)	107·3 (54·8–205·5)	26·8 (19·1–33·7)	526·9 (250·2–1129·5)	421·0 (195·7–932·7)	105·8 (53·3–195·6)	26·0 (20·9–31·3)
	Anxiety disorders	691·3 (471·7–944·9)	409·4 (276·1–564·3)	281·9 (194·4–385·6)	69·0 (62·5–75·4)	544·5 (377·5–733·9)	327·7 (224·3–449·7)	216·8 (150·4–293·5)	66·3 (59·1–72·8)
	COVID-19	3911·4 (3322·4–4545·6)	6638·4 (5666·9–7590·6)	-2727·0 (-3633·5–-1826·9)	-40·9 (-50·5–-29·7)	NA	NA	NA	NA
	Chronic kidney disease	305·6 (267·0–351·1)	379·9 (340·6–420·6)	-74·3 (-92·8–-41·0)	-19·6 (-24·6–-11·2)	277·2 (245·3–310·5)	362·0 (328·7–397·1)	-84·8 (-95·2–-75·3)	-23·5 (-26·5–-20·2)
	Chronic obstructive pulmonary disease	301·8 (277·3–328·0)	723·8 (682·2–766·3)	-421·9 (-459·4–-384·5)	-58·3 (-61·2–-55·0)	569·3 (538·6–597·3)	1603·9 (1556·1–1648·5)	-1034·6 (-1071·6–-1000·2)	-64·5 (-66·0–-63·0)
	Cirrhosis and other chronic liver diseases	651·1 (599·5–709·8)	1374·7 (1282·6–1469·6)	-723·6 (-827·8–-628·7)	-52·6 (-57·1–-47·9)	410·6 (401·8–419·1)	887·6 (869·4–906·6)	-477·0 (-496·6–-459·2)	-53·7 (-54·9–-52·7)
	Depressive disorders	952·5 (674·0–1290·3)	651·8 (453·0–887·4)	300·7 (207·5–408·3)	46·2 (41·7–49·9)	846·6 (585·3–1158·4)	566·6 (391·2–777·2)	280·0 (191·8–381·4)	49·4 (45·4–53·5)
	Diabetes mellitus	830·9 (694·1–1018·1)	912·4 (760·8–1101·8)	-81·5 (-118·2–-48·6)	-8·9 (-12·1–-5·6)	487·8 (413·0–581·9)	533·0 (450·5–639·8)	-45·2 (-63·9–-30·4)	-8·5 (-10·8–-6·1)
	Falls	484·4 (358·8–638·0)	1083·4 (868·9–1355·8)	-599·1 (-725·8–-500·2)	-55·4 (-58·9–-51·7)	624·1 (476·3–801·3)	1412·4 (1150·0–1729·7)	-788·3 (-957·4–-668·6)	-55·9 (-58·7–-53·1)
	HIV/AIDS	273·7 (263·4–287·1)	545·5 (525·9–569·9)	-271·7 (-284·2–-262·1)	-49·8 (-50·5–-48·8)	35·7 (35·2–36·6)	133·0 (130·5–137·2)	-97·3 (-100·7–-95·3)	-73·1 (-73·4–-73·0)
	Headache disorders	986·7 (302·6–2011·6)	559·5 (182·7–1150·0)	427·1 (117·6–890·1)	75·9 (49·1–89·2)	996·3 (311·9–2028·7)	558·5 (187·0–1142·6)	437·8 (122·3–904·2)	78·0 (50·8–92·2)
	Ischemic heart disease	3456·1 (3125·5–3795·6)	6789·3 (6257·3–7335·4)	-3333·2 (-3898·3–-2803·5)	-49·0 (-54·2–-43·6)	5024·1 (4767·5–5151·0)	10179·4 (9908·6–10365·1)	-5155·4 (-5269·4–-5021·3)	-50·6 (-52·2–-49·8)
	Low back pain	1807·1 (1298·6–2425·2)	1256·3 (892·5–1694·1)	550·8 (398·2–726·5)	43·9 (39·9–47·4)	1905·2 (1370·7–2551·0)	1293·4 (919·6–1743·9)	611·9 (445·6–809·6)	47·4 (43·2–51·3)
	Lower respiratory infections	234·3 (217·5–253·2)	626·4 (582·9–667·5)	-392·1 (-433·1–-354·2)	-62·6 (-65·8–-59·1)	230·7 (221·3–236·9)	517·6 (506·7–528·1)	-287·0 (-298·2–-276·8)	-55·4 (-57·1–-54·1)
	Other musculoskeletal disorders	365·5 (261·1–498·9)	87·6 (58·7–130·5)	277·9 (199·7–372·5)	323·3 (243·2–412·2)	278·0 (194·5–390·5)	32·5 (21·7–49·6)	245·5 (168·4–346·6)	774·9 (536·1–1083·9)

	Road injuries	306·8 (277·3–339·8)	1062·2 (983·4–1150·9)	-755·4 (-820·4–-689·8)	-71·1 (-73·2–-68·8)	654·5 (610·9–706·4)	2640·3 (2533·9–2764·9)	-1985·8 (-2057·7–-1919·9)	-75·2 (-76·0–-74·4)
	Stroke	2212·5 (2035·1–2373·6)	3284·1 (3070·0–3504·5)	-1071·6 (-1273·6–-871·0)	-32·6 (-37·5–-27·5)	4219·7 (4033·6–4349·1)	5498·0 (5343·3–5620·6)	-1278·3 (-1352·0–-1205·7)	-23·3 (-25·0–-22·0)
	Tracheal, bronchus, and lung cancer	338·0 (312·9–362·3)	1402·3 (1308·8–1485·2)	-1064·3 (-1149·8–-974·7)	-75·9 (-77·7–-73·9)	335·5 (325·0–343·7)	2567·3 (2515·0–2619·2)	-2231·8 (-2279·0–-2182·0)	-86·9 (-87·3–-86·6)
	Tuberculosis	69·1 (62·2–76·4)	230·4 (211·5–251·0)	-161·3 (-181·2–-143·6)	-70·0 (-73·2–-66·4)	120·2 (112·6–128·0)	512·6 (491·8–532·5)	-392·3 (-408·7–-376·9)	-76·5 (-77·4–-75·5)
High-income	Age-related and other hearing loss	384·2 (267·0–532·4)	489·0 (336·3–681·9)	-104·7 (-157·3–-66·0)	-21·3 (-23·6–-18·7)	389·6 (271·2–538·7)	498·4 (343·7–696·1)	-108·8 (-160·5–-69·4)	-21·8 (-24·1–-19·1)
	Alzheimer's disease and other dementias	640·1 (311·1–1291·3)	473·7 (216·8–1016·6)	166·5 (92·1–254·3)	37·2 (25·3–44·3)	650·7 (308·3–1367·7)	489·0 (224·0–1063·3)	161·7 (82·2–291·4)	34·3 (26·5–40·0)
	Anxiety disorders	1123·7 (769·6–1529·7)	611·6 (420·0–837·8)	512·2 (347·4–706·7)	84·0 (73·6–93·8)	896·2 (624·3–1217·1)	486·5 (330·4–669·7)	409·7 (276·5–562·7)	84·4 (73·4–94·4)
	COVID-19	1105·5 (1022·7–1269·1)	2104·4 (2045·7–2211·8)	-998·9 (-1042·9–-932·6)	-47·5 (-50·3–-42·1)	NA	NA	NA	NA
	Chronic kidney disease	349·5 (309·8–385·5)	479·5 (434·1–518·7)	-130·0 (-144·2–-114·5)	-27·1 (-30·1–-24·3)	277·3 (246·3–305·8)	388·0 (351·3–422·7)	-110·8 (-119·8–-101·3)	-28·6 (-30·4–-26·8)
	Chronic obstructive pulmonary disease	491·0 (448·8–524·6)	670·6 (627·5–708·0)	-179·6 (-204·0–-155·4)	-26·8 (-30·0–-23·4)	461·1 (426·7–491·2)	1005·9 (961·5–1047·2)	-544·8 (-572·9–-516·5)	-54·2 (-56·4–-52·1)
	Cirrhosis and other chronic liver diseases	220·7 (210·7–227·5)	484·9 (473·1–495·9)	-264·2 (-272·3–-255·9)	-54·5 (-55·7–-53·5)	325·6 (315·0–332·8)	862·7 (834·2–879·4)	-537·1 (-552·2–-512·7)	-62·3 (-63·2–-61·3)
	Depressive disorders	1300·3 (914·1–1744·1)	746·6 (518·4–1009·0)	553·7 (392·0–750·7)	74·2 (69·4–78·7)	968·9 (681·8–1307·2)	590·0 (407·5–803·8)	378·9 (270·1–505·2)	64·3 (60·4–68·5)
	Diabetes mellitus	710·7 (549·0–920·6)	988·6 (783·8–1247·8)	-277·9 (-335·6–-229·3)	-28·2 (-30·3–-26·3)	575·5 (500·5–675·6)	710·0 (614·3–836·5)	-134·6 (-160·5–-109·7)	-18·9 (-20·9–-17·4)
	Falls	579·9 (429·2–756·6)	703·6 (554·8–882·0)	-123·7 (-139·0–-108·0)	-17·9 (-23·3–-13·1)	592·4 (439·7–771·1)	846·4 (669·4–1060·9)	-253·9 (-297·5–-221·1)	-30·2 (-34·3–-26·6)
	HIV/AIDS	40·2 (34·5–47·4)	100·3 (86·8–117·7)	-60·2 (-71·2–-52·0)	-60·0 (-62·2–-57·5)	71·4 (69·0–74·7)	464·7 (453·3–479·7)	-393·3 (-405·1–-384·3)	-84·6 (-84·8–-84·4)
	Headache disorders	1072·3 (213·1–2236·8)	554·5 (129·2–1166·6)	517·7 (76·5–1137·4)	91·8 (46·9–130·3)	1075·9 (214·5–2258·9)	550·7 (128·4–1160·9)	525·2 (79·6–1143·3)	93·9 (49·0–135·2)
	Ischemic heart disease	717·0 (634·4–763·9)	1768·8 (1675·0–1822·8)	-1051·8 (-1074·5–-1028·7)	-59·5 (-62·6–-58·0)	2168·4 (1978·8–2268·2)	4768·3 (4603·8–4859·7)	-2599·9 (-2646·2–-2563·7)	-54·5 (-57·1–-53·2)
	Low back pain	1601·6 (1157·8–2137·8)	1156·2 (833·8–1541·6)	445·4 (323·2–583·7)	38·6 (36·8–40·5)	1690·5 (1216·4–2263·8)	1229·3 (882·5–1655·6)	461·2 (334·2–616·4)	37·6 (35·6–39·4)
	Lower respiratory infections	178·0 (155·8–190·9)	313·3 (289·8–326·6)	-135·3 (-140·9–-130·0)	-43·2 (-46·5–-40·8)	339·9 (309·0–356·1)	650·7 (616·6–668·0)	-310·8 (-317·1–-303·4)	-47·8 (-50·2–-46·3)
	Other musculoskeletal disorders	1006·9 (714·0–1354·7)	700·4 (494·4–954·7)	306·5 (221·0–401·1)	43·9 (40·4–47·8)	800·4 (570·9–1079·2)	491·9 (343·6–684·8)	308·5 (229·8–404·3)	63·3 (55·1–73·0)

	Road injuries	276·2 (250·3–310·8)	722·9 (669·6–781·1)	-446·7 (-476·1–-419·6)	-61·8 (-63·2–-60·2)	670·2 (617·5–735·3)	1875·7 (1775·0–1998·0)	-1205·5 (-1270·0–-1152·5)	-64·3 (-65·3–-63·1)
	Stroke	665·4 (591·9–725·4)	899·4 (835·1–960·0)	-233·9 (-258·6–-215·2)	-26·0 (-30·2–-23·5)	1604·8 (1474·4–1695·2)	2115·7 (2024·2–2197·6)	-510·9 (-558·3–-477·5)	-24·2 (-27·2–-22·3)
	Tracheal, bronchus, and lung cancer	503·7 (464·1–526·0)	954·0 (907·9–984·2)	-450·4 (-471·4–-430·8)	-47·2 (-49·2–-45·7)	561·1 (537·6–574·3)	1908·1 (1864·5–1940·1)	-1347·0 (-1372·2–-1320·3)	-70·6 (-71·3–-70·0)
	Tuberculosis	10·1 (9·1–11·0)	20·7 (19·3–22·1)	-10·5 (-11·6–-9·7)	-51·0 (-54·0–-48·3)	50·2 (47·4–53·4)	130·8 (123·0–142·6)	-80·6 (-92·6–-73·1)	-61·6 (-65·0–-58·8)
Latin America and Caribbean	Age-related and other hearing loss	535·8 (370·8–746·2)	582·7 (400·4–814·4)	-46·9 (-74·7–-28·4)	-8·0 (-9·5–-6·4)	541·5 (375·5–751·3)	587·2 (404·2–816·8)	-45·7 (-70·1–-27·3)	-7·7 (-9·2–-6·1)
	Alzheimer's disease and other dementias	541·5 (261·5–1129·3)	429·8 (202·0–913·0)	111·7 (56·3–205·3)	26·8 (20·7–32·2)	549·5 (262·3–1165·9)	430·5 (201·6–938·8)	119·1 (60·5–226·2)	28·4 (23·8–33·3)
	Anxiety disorders	1219·6 (841·4–1658·1)	680·7 (466·2–923·5)	538·9 (360·7–739·5)	79·4 (69·2–89·5)	829·9 (573·7–1123·1)	503·6 (344·1–688·8)	326·3 (217·7–441·8)	65·0 (55·0–74·2)
	COVID-19	4472·7 (3979·7–5079·5)	8124·1 (7382·4–8942·5)	-3651·3 (-4448·3–-2821·5)	-44·9 (-51·5–-37·2)	NA	NA	NA	NA
	Chronic kidney disease	903·7 (802·4–1026·5)	1160·5 (1056·5–1277·6)	-256·8 (-360·4–-133·3)	-22·1 (-29·9–-12·1)	705·5 (664·8–750·4)	854·2 (803·9–914·8)	-148·8 (-176·9–-120·9)	-17·4 (-20·4–-14·4)
	Chronic obstructive pulmonary disease	522·1 (476·2–562·4)	744·5 (686·5–795·8)	-222·5 (-256·3–-185·7)	-29·9 (-33·3–-25·6)	677·3 (634·3–713·5)	1065·8 (1022·5–1103·8)	-388·5 (-421·1–-358·9)	-36·5 (-39·2–-33·9)
	Cirrhosis and other chronic liver diseases	330·9 (294·9–367·7)	1133·1 (1024·2–1237·6)	-802·3 (-899·6–-708·8)	-70·8 (-73·8–-67·8)	526·1 (506·3–545·5)	1638·1 (1603·3–1670·4)	-1111·9 (-1142·6–-1081·2)	-67·9 (-68·9–-66·8)
	Depressive disorders	1139·0 (783·3–1561·2)	623·5 (431·2–844·4)	515·5 (342·6–718·1)	82·7 (72·4–92·3)	965·2 (669·6–1315·4)	528·2 (364·6–723·6)	437·0 (294·9–605·9)	82·8 (72·5–91·9)
	Diabetes mellitus	1754·0 (1501·3–2093·5)	1845·0 (1601·8–2152·4)	-90·9 (-198·8–10·5)	-4·9 (-10·5–0·5)	1744·6 (1573·0–1950·9)	1502·2 (1344·7–1698·3)	242·4 (206·1–276·5)	16·2 (13·7–18·5)
	Falls	389·9 (307·1–491·1)	753·1 (620·0–908·2)	-363·2 (-426·4–-310·3)	-48·3 (-51·4–-44·9)	494·5 (388·2–620·5)	1109·5 (922·8–1329·4)	-615·0 (-724·3–-533·0)	-55·5 (-58·6–-52·4)
	HIV/AIDS	236·9 (220·7–259·1)	506·3 (488·5–528·2)	-269·4 (-294·6–-244·9)	-53·2 (-56·5–-48·9)	217·6 (176·7–283·4)	497·5 (461·4–567·5)	-279·9 (-322·0–-247·0)	-56·4 (-62·3–-47·8)
	Headache disorders	972·8 (165·9–2062·8)	533·8 (102·0–1133·8)	439·0 (58·9–972·2)	80·3 (48·3–99·0)	971·8 (166·2–2063·7)	525·4 (101·7–1129·2)	446·4 (59·0–977·6)	82·9 (47·0–102·9)
	Ischemic heart disease	1538·7 (1401·0–1663·8)	2892·9 (2675·6–3132·6)	-1354·2 (-1526·1–-1188·9)	-46·8 (-50·0–-43·5)	2663·4 (2532·2–2744·0)	4204·2 (4065·7–4290·9)	-1540·8 (-1613·0–-1447·7)	-36·7 (-38·4–-34·8)
	Low back pain	1349·2 (972·0–1814·5)	809·4 (577·0–1089·4)	539·7 (391·8–723·2)	66·7 (62·7–71·0)	1345·0 (958·9–1807·3)	783·5 (558·0–1050·3)	561·6 (400·6–752·5)	71·7 (67·5–76·4)
	Lower respiratory infections	554·8 (497·2–605·9)	828·3 (765·4–896·5)	-273·5 (-322·1–-230·3)	-33·0 (-37·7–-28·5)	817·3 (767·3–856·1)	1138·5 (1094·6–1179·6)	-321·2 (-357·0–-281·8)	-28·2 (-31·2–-24·8)

	Other musculoskeletal disorders	985·1 (706·6–1321·7)	701·3 (491·1–965·3)	283·8 (216·6–356·5)	40·9 (35·0–48·4)	944·3 (678·6–1262·3)	645·0 (452·4–894·0)	299·3 (226·7–382·1)	46·9 (40·0–54·2)
	Road injuries	389·2 (357·3–426·2)	1712·6 (1602·7–1845·0)	-1323·4 (-1424·2–-1240·0)	-77·3 (-78·3–-76·2)	686·4 (652·3–725·0)	2621·1 (2530·5–2724·8)	-1934·7 (-2001·3–-1875·4)	-73·8 (-74·4–-73·2)
	Stroke	1143·0 (1042·7–1234·0)	1437·6 (1338·0–1539·0)	-294·6 (-359·3–-237·4)	-20·5 (-24·2–-16·7)	2532·4 (2406·4–2617·3)	2905·2 (2805·5–2980·8)	-372·8 (-444·9–-297·4)	-12·8 (-15·4–-10·4)
	Tracheal, bronchus, and lung cancer	295·4 (269·1–320·8)	480·2 (445·9–518·7)	-184·8 (-213·7–-160·1)	-38·5 (-42·6–-34·8)	286·4 (275·3–294·9)	730·4 (710·3–749·5)	-444·1 (-459·6–-428·3)	-60·8 (-61·7–-59·9)
	Tuberculosis	84·3 (74·6–95·9)	195·4 (171·1–246·0)	-111·1 (-161·4–-93·4)	-56·6 (-65·6–-52·3)	426·8 (398·2–453·1)	772·3 (704·7–958·3)	-345·5 (-523·7–-275·6)	-44·5 (-55·1–-38·7)
North Africa and Middle East	Age-related and other hearing loss	553·8 (392·5–757·1)	611·5 (425·2–849·3)	-57·7 (-94·5–-32·9)	-9·4 (-11·7–-6·8)	581·3 (413·5–795·0)	643·6 (451·9–894·8)	-62·3 (-98·4–-36·5)	-9·6 (-11·8–-7·1)
	Alzheimer's disease and other dementias	659·4 (315·5–1391·8)	524·3 (245·0–1117·8)	135·1 (70·2–251·5)	26·7 (20·1–33·5)	710·7 (344·8–1522·2)	567·1 (264·4–1244·7)	143·6 (70·4–276·6)	26·2 (18·9–33·2)
	Anxiety disorders	1054·1 (698·0–1471·3)	626·4 (417·9–881·8)	427·8 (275·5–610·5)	68·7 (53·0–84·5)	872·1 (595·3–1180·9)	524·6 (362·1–719·1)	347·5 (232·4–490·5)	66·5 (53·6–80·1)
	COVID-19	3418·7 (3004·5–3943·4)	6428·2 (5683·5–7199·9)	-3009·6 (-3381·6–-2570·4)	-46·8 (-49·3–-42·3)	NA	NA	NA	NA
	Chronic kidney disease	1040·3 (907·6–1173·2)	1021·6 (871·2–1171·4)	18·7 (-122·2–150·8)	2·2 (-10·5–16·7)	880·3 (734·1–1139·4)	919·2 (724·0–1484·8)	-38·9 (-594·8–302·9)	-1·1 (-40·3–40·9)
	Chronic obstructive pulmonary disease	621·9 (551·4–695·6)	870·4 (786·6–962·2)	-248·5 (-333·8–-170·7)	-28·5 (-36·6–-20·9)	778·1 (554·8–919·9)	1199·7 (965·5–1338·6)	-421·5 (-651·0–-193·7)	-35·0 (-52·5–-19·7)
	Cirrhosis and other chronic liver diseases	548·2 (465·5–642·7)	810·8 (691·6–954·8)	-262·6 (-348·5–-178·1)	-32·3 (-39·2–-23·6)	1250·6 (1061·2–1473·2)	1511·6 (1310·6–1759·4)	-260·9 (-489·0–26·0)	-17·0 (-30·9–1·9)
	Depressive disorders	1370·2 (902·0–1884·5)	878·3 (593·3–1207·7)	491·9 (324·2–703·2)	56·1 (48·3–63·2)	1207·4 (824·9–1656·9)	756·2 (513·4–1030·9)	451·1 (305·2–626·4)	59·7 (53·6–65·2)
	Diabetes mellitus	1712·1 (1410·6–2112·9)	1625·2 (1313·8–2039·4)	87·0 (14·0–201·4)	5·4 (0·8–13·6)	1075·7 (948·9–1247·2)	918·3 (794·6–1093·5)	157·4 (47·7–238·0)	17·4 (4·7–26·4)
	Falls	316·5 (245·6–399·3)	538·3 (439·7–661·7)	-221·8 (-277·0–-180·1)	-41·3 (-47·0–-36·1)	338·8 (264·6–421·9)	591·5 (487·1–726·1)	-252·7 (-342·4–-200·6)	-42·7 (-50·5–-36·5)
	HIV/AIDS	104·0 (65·3–200·8)	91·3 (60·8–185·4)	12·6 (-36·9–52·0)	15·3 (-22·7–50·6)	12·2 (8·3–24·2)	15·3 (10·6–27·7)	-3·2 (-6·9–0·3)	-21·7 (-34·6–2·1)
	Headache disorders	1019·2 (237·0–2103·9)	627·0 (165·7–1301·4)	392·2 (70·9–853·7)	60·6 (37·4–77·9)	1012·2 (238·9–2080·4)	625·4 (164·8–1285·4)	386·7 (73·3–843·5)	60·0 (37·2–76·6)
	Ischemic heart disease	4102·1 (3547·4–4710·9)	5862·0 (5201·4–6561·6)	-1760·0 (-2260·1–-1301·9)	-30·0 (-37·0–-23·0)	5930·0 (5279·8–6750·8)	8355·2 (7634·3–9082·0)	-2425·2 (-3251·3–-1478·8)	-28·9 (-36·9–-18·4)
	Low back pain	1413·5 (1016·8–1878·8)	992·1 (709·9–1332·9)	421·4 (301·1–555·3)	42·5 (38·6–46·3)	1426·1 (1037·3–1916·2)	1047·1 (751·1–1406·1)	379·0 (274·6–516·6)	36·3 (32·7–40·7)
	Lower respiratory infections	427·7 (340·1–500·9)	513·0 (452·1–574·9)	-85·3 (-173·8–-31·0)	-16·5 (-33·8–-6·1)	663·4 (578·9–867·2)	737·1 (663·4–810·4)	-73·8 (-167·5–127·5)	-9·9 (-22·0–18·6)

	Other musculoskeletal disorders	660·5 (465·2–894·3)	400·8 (275·7–568·5)	259·6 (192·9–342·2)	65·5 (55·1–75·1)	500·3 (352·6–673·9)	269·0 (178·2–383·5)	231·3 (170·6–298·5)	87·1 (72·9–104·3)
	Road injuries	548·6 (470·9–621·3)	1834·6 (1655·1–2034·4)	-1286·0 (-1436·8–-1146·8)	-70·1 (-73·5–-67·9)	1007·7 (898·1–1123·2)	3065·1 (2849·9–3296·1)	-2057·4 (-2235·7–-1880·9)	-67·1 (-70·0–-64·3)
	Stroke	2474·4 (2151·3–2831·8)	2486·7 (2196·2–2801·8)	-12·2 (-321·4–283·1)	-0·4 (-12·0–11·4)	4213·0 (3578·2–4818·9)	4262·0 (3730·6–4669·2)	-49·0 (-734·0–518·5)	-1·0 (-16·4–12·9)
	Tracheal, bronchus, and lung cancer	208·8 (175·9–243·3)	799·1 (691·8–938·0)	-590·3 (-712·1–-493·5)	-73·8 (-77·6–-70·1)	165·0 (134·2–207·0)	1019·7 (831·6–1229·4)	-854·7 (-1055·2–-661·8)	-83·7 (-87·5–-77·7)
	Tuberculosis	183·2 (148·1–224·7)	147·4 (101·3–261·0)	35·9 (-67·9–85·7)	29·7 (-24·1–81·9)	655·8 (552·7–761·7)	592·0 (349·0–1282·5)	63·8 (-594·9–346·7)	20·6 (-46·2–97·1)
South Asia	Age-related and other hearing loss	675·4 (479·2–930·1)	717·7 (499·8–990·1)	-42·3 (-79·5–-16·4)	-5·8 (-8·6–-2·5)	684·4 (487·8–939·8)	725·7 (508·1–999·6)	-41·3 (-78·2–-15·1)	-5·6 (-8·5–-2·5)
	Alzheimer's disease and other dementias	433·0 (188·5–963·3)	325·3 (143·9–729·0)	107·7 (41·0–251·4)	33·2 (21·6–48·5)	384·4 (176·8–873·1)	295·9 (138·7–661·9)	88·5 (32·5–216·6)	30·3 (15·4–46·4)
	Anxiety disorders	606·4 (420·2–816·1)	417·2 (287·6–562·9)	189·2 (131·0–253·6)	45·5 (38·1–51·9)	504·1 (350·3–681·1)	362·5 (250·8–492·4)	141·6 (96·4–193·2)	39·2 (32·3–45·5)
	COVID-19	3232·9 (2940·0–3732·4)	6089·9 (5794·2–6519·3)	-2856·9 (-3070·5–-2632·0)	-47·0 (-49·8–-41·7)	NA	NA	NA	NA
	Chronic kidney disease	569·5 (487·7–660·7)	752·2 (617·2–889·6)	-182·7 (-322·0–-53·0)	-23·8 (-37·3–-8·0)	509·2 (433·0–590·2)	672·8 (554·9–786·1)	-163·6 (-280·3–-37·9)	-23·9 (-36·1–-6·6)
	Chronic obstructive pulmonary disease	2198·4 (1794·1–2588·7)	2926·7 (2401·9–3304·0)	-728·3 (-1292·8–7·8)	-24·4 (-41·7–0·3)	2488·7 (1671·3–3139·9)	3506·4 (2538·4–4093·7)	-1017·7 (-1976·1–-81·1)	-28·4 (-53·5–-3·0)
	Cirrhosis and other chronic liver diseases	574·7 (432·1–742·6)	1244·8 (963·1–1558·1)	-670·1 (-977·6–-444·5)	-53·5 (-66·7–-39·2)	863·7 (668·4–1100·1)	1641·2 (1335·7–2116·2)	-777·5 (-1174·7–-431·3)	-46·9 (-58·6–-30·4)
	Depressive disorders	1122·8 (784·9–1510·0)	812·8 (562·7–1104·5)	310·0 (214·0–422·1)	38·2 (34·4–41·8)	1091·7 (758·9–1472·1)	757·6 (523·9–1032·0)	334·1 (229·8–452·4)	44·1 (40·8–47·6)
	Diabetes mellitus	1385·1 (1189·3–1629·6)	1487·2 (1244·6–1767·1)	-102·1 (-279·5–52·7)	-6·6 (-17·2–3·7)	937·4 (816·6–1062·2)	1024·2 (870·6–1167·1)	-86·8 (-202·1–29·0)	-8·3 (-18·6–3·0)
	Falls	875·7 (720·3–1035·0)	889·1 (747·0–1036·0)	-13·3 (-155·4–118·2)	-1·3 (-16·2–14·2)	949·6 (775·0–1137·0)	1016·2 (840·0–1188·9)	-66·6 (-217·2–90·3)	-6·4 (-21·4–9·1)
	HIV/AIDS	153·4 (116·8–244·9)	158·2 (112·0–249·8)	-4·9 (-79·4–48·3)	-0·8 (-34·1–31·4)	1·4 (0·8–2·6)	3·0 (1·6–5·6)	-1·5 (-2·9–-0·7)	-51·2 (-55·6–-45·9)
	Headache disorders	855·9 (149·1–1841·9)	591·2 (100·7–1246·7)	264·7 (46·0–584·6)	44·9 (24·3–57·3)	857·6 (150·4–1882·9)	587·7 (104·1–1237·6)	270·0 (44·5–594·9)	45·9 (25·0–58·2)
	Ischemic heart disease	3008·7 (2676·5–3344·8)	5385·2 (4788·3–6025·8)	-2376·6 (-3117·6–-1679·8)	-43·9 (-52·8–-34·2)	3281·4 (2795·3–3739·8)	4799·0 (4295·1–5338·1)	-1517·6 (-2222·4–-830·2)	-31·4 (-43·0–-18·5)
	Low back pain	1291·5 (926·3–1736·3)	597·8 (424·0–803·8)	693·7 (497·2–925·2)	116·1 (109·5–122·6)	1426·7 (1025·4–1903·9)	711·3 (504·5–957·2)	715·3 (518·9–950·5)	100·7 (94·2–106·8)

	Lower respiratory infections	679·6 (541·4–871·2)	754·0 (635·4–884·5)	-74·5 (-242·6–107·7)	-9·4 (-30·0–14·5)	973·0 (762·4–1289·3)	992·3 (857·4–1107·6)	-19·3 (-227·0–268·5)	-1·7 (-22·3–26·6)
	Other musculoskeletal disorders	1140·7 (811·7–1537·3)	751·9 (522·2–1023·6)	388·8 (282·1–517·9)	51·9 (46·8–56·9)	926·6 (657·6–1250·5)	638·5 (450·7–865·4)	288·0 (205·0–384·1)	45·2 (40·4–50·3)
	Road injuries	341·7 (292·3–386·9)	1401·6 (1264·9–1539·6)	-1059·9 (-1197·8–-915·4)	-75·6 (-79·4–-71·6)	403·2 (355·1–447·1)	1390·8 (1255·8–1525·4)	-987·6 (-1110·8–-844·6)	-71·0 (-74·7–-66·6)
	Stroke	2000·6 (1761·3–2261·2)	2469·2 (2190·7–2772·8)	-468·6 (-819·0–-131·3)	-18·7 (-30·9–-5·6)	2776·4 (2337·4–3133·3)	3178·2 (2745·0–3547·0)	-401·7 (-985·7–127·0)	-12·3 (-29·7–4·5)
	Tracheal, bronchus, and lung cancer	123·6 (105·6–144·6)	318·9 (260·1–371·0)	-195·3 (-248·2–-143·3)	-61·0 (-67·8–-52·8)	74·5 (61·3–85·7)	313·4 (267·5–368·5)	-238·8 (-291·7–-196·0)	-76·1 (-79·9–-70·9)
	Tuberculosis	1002·5 (864·6–1162·0)	1638·9 (1373·7–2045·7)	-636·5 (-1053·4–-348·5)	-38·3 (-52·1–-24·2)	3546·6 (3090·4–4014·9)	5194·8 (4108·0–6137·7)	-1648·2 (-2671·4–-517·5)	-31·1 (-44·6–-12·1)
Southeast Asia, East Asia, and Oceania	Age-related and other hearing loss	730·3 (504·1–1010·3)	803·6 (552·6–1118·3)	-73·3 (-112·8–-46·4)	-9·1 (-11·0–-7·3)	683·7 (470·7–950·6)	748·6 (513·7–1042·1)	-64·8 (-101·3–-39·7)	-8·6 (-10·5–-6·8)
	Alzheimer's disease and other dementias	739·1 (357·8–1568·5)	536·1 (247·3–1207·6)	203·0 (96·2–387·2)	40·2 (18·6–58·2)	685·8 (311·1–1485·2)	488·5 (215·8–1111·5)	197·3 (89·9–405·6)	41·8 (25·9–57·4)
	Anxiety disorders	665·0 (462·2–912·9)	394·2 (269·0–544·4)	270·8 (187·2–370·8)	68·8 (62·2–75·2)	597·5 (415·4–816·9)	362·8 (250·8–498·2)	234·7 (162·0–324·8)	64·8 (58·3–71·6)
	COVID-19	567·0 (392·7–857·5)	1028·6 (720·4–1608·0)	-461·6 (-746·5–-307·0)	-44·7 (-48·1–-38·5)	NA	NA	NA	NA
	Chronic kidney disease	507·4 (444·7–574·3)	633·5 (544·4–721·6)	-126·1 (-215·1–-32·3)	-19·5 (-31·4–-5·7)	611·8 (539·8–690·7)	662·4 (556·1–788·0)	-50·7 (-170·7–70·8)	-7·1 (-22·2–11·7)
	Chronic obstructive pulmonary disease	1106·1 (916·1–1347·3)	1914·3 (1580·7–2222·1)	-808·2 (-1177·1–-428·3)	-41·8 (-54·5–-24·5)	3327·7 (2587·8–3841·9)	4592·1 (3995·1–5292·8)	-1264·5 (-2285·1–-434·9)	-27·2 (-44·9–-10·6)
	Cirrhosis and other chronic liver diseases	262·5 (223·7–314·9)	731·7 (625·7–859·7)	-469·3 (-601·7–-361·1)	-63·9 (-71·9–-55·5)	639·4 (511·8–779·9)	1324·5 (1112·7–1615·2)	-685·0 (-934·7–-425·8)	-51·4 (-61·5–-36·6)
	Depressive disorders	676·5 (474·5–912·9)	449·9 (313·9–614·0)	226·6 (160·3–301·5)	50·4 (47·2–53·7)	708·9 (494·6–956·0)	430·1 (301·0–584·0)	278·8 (194·4–375·5)	64·9 (60·8–68·7)
	Diabetes mellitus	882·5 (730·0–1073·2)	966·3 (784·8–1198·2)	-83·8 (-158·3–-18·9)	-8·6 (-15·3–-2·3)	760·7 (652·9–885·7)	701·2 (592·5–827·9)	59·5 (-8·0–136·5)	8·7 (-1·2–20·3)
	Falls	391·3 (305·3–490·3)	683·4 (558·5–832·6)	-292·1 (-372·9–-214·9)	-42·7 (-50·2–-35·1)	422·4 (341·4–511·7)	717·2 (595·1–882·1)	-294·8 (-437·3–-223·1)	-40·9 (-52·4–-33·4)
	HIV/AIDS	90·2 (80·1–102·8)	222·8 (195·0–263·3)	-132·7 (-163·8–-110·9)	-59·5 (-62·9–-56·4)	25·6 (22·6–28·0)	69·8 (63·1–75·7)	-44·3 (-47·7–-40·1)	-63·4 (-64·7–-62·3)
	Headache disorders	819·1 (123·1–1740·7)	530·6 (115·3–1108·4)	288·5 (6·9–694·2)	50·2 (6·0–78·5)	770·5 (121·6–1647·8)	490·5 (116·0–1016·6)	279·9 (5·2–665·8)	52·7 (4·8–81·4)
	Ischemic heart disease	1827·9 (1521·6–2133·0)	3275·7 (2782·3–3808·0)	-1447·8 (-2031·7–-961·9)	-43·9 (-55·1–-32·6)	2101·4 (1867·1–2399·4)	2767·5 (2446·7–3106·0)	-666·1 (-1088·8–-248·7)	-23·8 (-35·5–-10·0)
	Low back pain	948·0 (674·2–1268·0)	598·3 (425·7–806·9)	349·7 (246·9–471·9)	58·5 (53·8–63·1)	1129·4 (804·0–1523·8)	678·2 (479·6–921·4)	451·3 (321·2–609·2)	66·6 (61·6–71·6)
	Lower respiratory infections	274·1 (224·8–319·5)	487·5 (436·5–545·7)	-213·4 (-277·0–-155·5)	-43·7 (-54·6–-33·5)	583·6 (449·2–676·5)	848·5 (747·6–946·9)	-264·9 (-430·2–-131·0)	-31·0 (-48·4–-16·7)

	Other musculoskeletal disorders	630·7 (449·3–862·3)	452·9 (313·9–630·9)	177·8 (132·3–234·7)	39·6 (34·7–45·3)	538·1 (383·8–731·3)	376·3 (259·8–526·2)	161·8 (121·5–205·7)	43·4 (37·2–50·7)
	Road injuries	428·9 (364·6–493·0)	1488·4 (1306·9–1684·5)	-1059·5 (-1261·8–-882·6)	-71·1 (-75·9–-66·0)	696·2 (613·1–825·8)	1874·4 (1678·7–2065·9)	-1178·2 (-1392·6–-969·2)	-62·8 (-68·5–-55·6)
	Stroke	2756·9 (2368·6–3157·4)	4438·5 (3804·8–5127·7)	-1681·5 (-2459·7–-992·2)	-37·6 (-48·9–-24·9)	5169·0 (4544·5–5909·3)	6432·2 (5620·4–7269·9)	-1263·2 (-2297·7–-305·4)	-19·3 (-32·6–-5·1)
	Tracheal, bronchus, and lung cancer	610·6 (496·2–734·8)	1390·6 (1079·5–1735·0)	-780·0 (-1144·7–-472·6)	-55·5 (-66·5–-41·8)	569·6 (478·8–672·7)	1387·5 (1146·2–1651·7)	-818·0 (-1111·1–-567·1)	-58·6 (-68·1–-46·7)
	Tuberculosis	243·9 (215·0–280·6)	500·5 (434·4–606·4)	-256·6 (-360·2–-189·8)	-51·0 (-60·3–-41·5)	1043·5 (951·2–1142·3)	1656·5 (1205·6–1945·0)	-612·9 (-917·5–-186·2)	-36·4 (-46·7–-15·6)
Sub-Saharan Africa	Age-related and other hearing loss	656·7 (469·3–908·1)	689·6 (491·9–957·4)	-32·9 (-58·7–-15·6)	-4·7 (-6·6–-2·9)	648·2 (464·0–896·0)	698·5 (498·1–968·3)	-50·2 (-80·2–-28·4)	-7·1 (-8·9–-5·2)
	Alzheimer's disease and other dementias	603·7 (269·5–1345·1)	369·4 (158·9–851·5)	234·3 (105·5–496·7)	64·8 (52·3–75·6)	559·5 (258·8–1200·4)	362·2 (162·5–813·5)	197·3 (90·0–419·4)	55·9 (40·6–70·4)
	Anxiety disorders	611·1 (414·7–845·2)	447·4 (297·9–621·0)	163·6 (110·9–225·6)	36·7 (30·0–43·2)	541·4 (372·2–735·4)	392·8 (264·0–537·0)	148·7 (99·7–202·4)	38·0 (30·9–44·6)
	COVID-19	5565·1 (5065·2–6245·1)	10559·3 (9751·8–11351·7)	-4994·2 (-5378·3–-4524·2)	-47·3 (-49·2–-43·7)	NA	NA	NA	NA
	Chronic kidney disease	947·3 (837·7–1076·8)	1391·3 (1206·7–1598·5)	-444·0 (-616·2–-265·7)	-31·7 (-41·4–-21·4)	924·9 (821·5–1048·5)	1380·4 (1166·4–1641·2)	-455·5 (-727·3–-227·6)	-32·5 (-45·4–-19·0)
	Chronic obstructive pulmonary disease	718·6 (598·7–890·4)	1059·0 (903·2–1202·8)	-340·4 (-527·7–-94·5)	-31·7 (-45·9–-10·7)	819·1 (601·7–992·7)	1336·9 (1022·1–1513·9)	-517·8 (-740·3–-118·3)	-38·2 (-52·6–-12·3)
	Cirrhosis and other chronic liver diseases	776·8 (670·2–899·1)	1678·5 (1425·7–1948·9)	-901·7 (-1126·3–-684·5)	-53·6 (-59·3–-46·7)	1090·9 (914·6–1279·4)	2278·0 (1947·4–2838·8)	-1187·0 (-1690·1–-819·6)	-51·8 (-61·2–-40·2)
	Depressive disorders	1256·2 (864·2–1698·4)	923·5 (633·2–1259·0)	332·8 (226·9–445·9)	36·1 (32·8–39·6)	1217·2 (837·6–1636·1)	861·8 (595·4–1162·8)	355·4 (243·4–479·7)	41·3 (38·5–44·2)
	Diabetes mellitus	1556·7 (1383·7–1788·3)	1908·7 (1700·9–2183·4)	-352·0 (-489·1–-220·6)	-18·4 (-24·6–-12·1)	1270·4 (1131·6–1419·1)	1576·2 (1411·2–1773·9)	-305·8 (-486·8–-139·6)	-19·2 (-29·1–-9·4)
	Falls	325·9 (275·2–389·6)	544·8 (457·9–656·8)	-218·9 (-313·4–-147·1)	-39·9 (-49·7–-30·1)	335·6 (280·2–408·0)	611·3 (522·7–726·6)	-275·7 (-367·8–-187·2)	-44·9 (-54·9–-32·7)
	HIV/AIDS	4299·5 (3866·2–4809·4)	3432·8 (3113·7–3872·7)	866·7 (371·4–1333·9)	25·5 (10·4–39·9)	3965·8 (2718·7–5844·4)	2814·7 (1817·9–4238·8)	1151·2 (811·8–1718·5)	41·6 (28·8–53·5)
	Headache disorders	741·1 (172·8–1543·2)	510·5 (120·9–1057·8)	230·6 (47·4–482·3)	45·0 (26·5–52·1)	732·8 (172·0–1510·8)	508·8 (122·4–1043·8)	224·0 (47·6–469·4)	43·9 (25·6–50·5)
	Ischemic heart disease	1867·5 (1633·2–2206·5)	2825·0 (2487·2–3180·3)	-957·5 (-1344·3–-558·5)	-33·7 (-43·4–-21·2)	2088·4 (1784·5–2405·0)	2700·3 (2366·9–3061·9)	-611·9 (-1081·4–-183·6)	-22·4 (-36·7–-7·8)

	Low back pain	1150·8 (822·0–1551·6)	815·5 (582·3–1097·7)	335·3 (240·5–455·8)	41·2 (38·4–43·9)	1197·2 (856·8–1607·3)	836·2 (596·2–1124·9)	361·0 (260·3–484·5)	43·2 (40·2–46·2)
	Lower respiratory infections	1528·1 (1238·7–1777·9)	2256·3 (2016·8–2496·2)	-728·2 (-1010·4–-479·0)	-32·2 (-43·5–-22·4)	2204·2 (1777·8–2540·0)	3124·4 (2750·5–3517·3)	-920·1 (-1387·8–-449·5)	-29·3 (-42·3–-15·2)
	Other musculoskeletal disorders	490·3 (355·3–665·2)	355·1 (244·8–494·8)	135·2 (103·0–173·1)	38·6 (32·1–47·7)	437·5 (314·2–591·2)	316·3 (219·6–440·6)	121·2 (91·5–154·5)	38·8 (31·7–49·0)
	Road injuries	632·7 (542·6–734·6)	1953·4 (1725·6–2185·9)	-1320·7 (-1495·5–-1152·3)	-67·6 (-70·3–-64·1)	904·6 (802·1–997·8)	2415·4 (2180·7–2666·3)	-1510·7 (-1738·7–-1290·3)	-62·5 (-66·5–-58·0)
	Stroke	2990·6 (2626·2–3394·6)	3643·6 (3228·9–4092·8)	-653·1 (-1029·1–-212·1)	-17·8 (-27·0–-6·0)	4064·4 (3510·8–4616·4)	4830·8 (4287·4–5309·7)	-766·4 (-1488·6–-21·5)	-15·6 (-29·2–-0·5)
	Tracheal, bronchus, and lung cancer	126·3 (105·7–145·7)	342·7 (303·2–394·9)	-216·4 (-254·0–-183·6)	-63·1 (-67·5–-58·6)	106·1 (87·7–124·6)	365·9 (310·0–437·9)	-259·8 (-323·4–-198·1)	-70·8 (-76·0–-63·0)
	Tuberculosis	1693·1 (1473·2–1981·9)	3134·2 (2468·7–3844·6)	-1441·1 (-2071·9–-660·6)	-45·5 (-55·7–-25·9)	4141·0 (3605·8–4998·2)	6796·6 (4991·2–8499·7)	-2655·7 (-4296·0–-824·8)	-38·3 (-51·7–-16·5)

The health outcomes presented here reflect the top 20 causes of disease burden, measured by Disability-Adjusted Life Years (DALYs), observed across females and males for the age group of 10 years and older globally in 2021. Health conditions are ordered alphabetically for each location. The absolute differences between females and males were calculated as the DALY rate for females minus the rate for males for each specific cause and year, with a positive value indicating a higher rate for females compared to males. The relative gap was computed as a relative percent difference, with a positive value indicating higher values among females relative to males. Cell colours denote whether the absolute and relative gaps in DALY rates indicate that the cause disproportionately affects females (red) or males (blue).

DALYs = Disability-Adjusted Life Years. 95% UI = 95% uncertainty interval. COVID = Coronavirus disease. HIV = Human Immunodeficiency Virus. AIDS = Acquired Immunodeficiency Syndrome.

Table S6. Global Disability-Adjusted Life Year (DALY) rates (per 100,000 population) for females and males by age and year and the absolute and relative gaps between females and males for the top 20 global causes

Age group	Cause	2021 DALYs per 100,000 among Females (95% UI)	2021 DALYs per 100,000 among Males (95% UI)	2021 Absolute gap in DALYs per 100,000	2021 Relative % gap in DALYs per 100,000	1990 DALYs per 100,000 among Females (95% UI)	1990 DALYs per 100,000 among Males (95% UI)	1990 Absolute gap in DALYs per 100,000	1990 Relative % gap in DALYs per 100,000
10-24 years old	Age-related and other hearing loss	142·6 (89·7–209·8)	162·1 (100·7–234·6)	-19·5 (-30·2–-11·3)	-12·0 (-14·5–-9·4)	130·1 (82·6–189·9)	150·0 (94·0–218·8)	-19·8 (-30·0–-11·4)	-13·2 (-15·8–-10·4)
	Alzheimer's disease and other dementias	0·0 (0·0–0·0)	0·0 (0·0–0·0)	0·0 (0·0–0·0)	NA (NA–NA)	0·0 (0·0–0·0)	0·0 (0·0–0·0)	0·0 (0·0–0·0)	NA (NA–NA)
	Anxiety disorders	760·2 (479·6–1113·4)	468·0 (293·5–690·1)	292·2 (181·4–419·1)	62·6 (54·5–70·5)	627·0 (395·9–914·8)	388·0 (245·1–574·3)	239·0 (150·8–344·9)	61·8 (54·1–68·9)
	COVID-19	461·7 (329·5–741·7)	475·1 (381·9–691·9)	-13·4 (-55·4–55·7)	-3·7 (-14·3–9·2)	NA	NA	NA	NA
	Chronic kidney disease	100·6 (88·7–113·8)	130·7 (112·9–149·7)	-30·0 (-45·4–-10·2)	-22·7 (-32·3–-9·4)	109·3 (96·3–123·7)	126·8 (105·7–144·3)	-17·5 (-33·3–1·1)	-13·4 (-23·9–1·0)
	Chronic obstructive pulmonary disease	32·3 (27·1–37·8)	33·0 (28·6–37·3)	-0·7 (-5·5–5·4)	-1·9 (-15·4–18·0)	44·2 (34·6–51·2)	48·8 (42·3–55·8)	-4·6 (-15·7–3·0)	-9·2 (-29·0–6·3)
	Cirrhosis and other chronic liver diseases	125·8 (108·5–144·5)	125·2 (112·3–142·0)	0·5 (-22·8–22·6)	0·7 (-16·6–19·4)	157·9 (130·9–191·6)	168·7 (142·0–200·6)	-10·8 (-43·0–26·0)	-5·8 (-23·5–17·2)
	Depressive disorders	703·0 (447·0–1020·9)	439·1 (279·5–646·9)	263·8 (166·1–381·9)	60·2 (55·2–65·2)	573·8 (369·6–827·3)	338·1 (216·2–494·1)	235·7 (153·4–338·1)	69·8 (66·5–73·5)
	Diabetes mellitus	90·2 (71·6–115·1)	87·1 (67·9–112·3)	3·1 (-2·1–8·8)	3·8 (-2·4–11·1)	72·4 (61·1–88·3)	60·5 (49·8–74·0)	11·9 (6·7–19·4)	19·8 (10·6–32·2)
	Falls	130·4 (100·5–167·9)	233·6 (196·8–276·5)	-103·3 (-125·9–-84·9)	-44·3 (-51·9–-36·4)	175·0 (134·3–228·0)	353·4 (296·5–420·6)	-178·4 (-217·6–-148·1)	-50·6 (-57·6–-44·3)
	HIV/AIDS	347·5 (271·3–445·4)	195·2 (159·4–236·7)	152·4 (85·8–238·1)	78·9 (43·9–127·2)	237·9 (168·6–358·1)	70·6 (55·1–95·3)	167·4 (110·8–264·1)	235·1 (186·9–296·9)
	Headache disorders	789·9 (85·2–1864·9)	501·7 (58·3–1156·4)	288·1 (25·5–692·0)	56·4 (34·0–69·9)	782·4 (83·8–1852·9)	477·9 (58·1–1097·4)	304·4 (23·9–731·5)	61·9 (34·4–79·0)
	Ischemic heart disease	81·7 (72·8–91·2)	117·6 (108·0–128·6)	-35·9 (-48·4–-24·1)	-30·4 (-38·3–-21·7)	106·8 (91·0–124·4)	123·7 (113·8–136·1)	-16·9 (-38·3–3·5)	-13·5 (-29·7–3·0)
	Low back pain	492·9 (320·1–695·4)	295·8 (190·2–416·9)	197·1 (129·3–284·1)	66·7 (59·5–73·4)	526·0 (342·7–748·4)	311·1 (200·2–439·2)	214·8 (140·5–309·2)	69·1 (62·5–76·2)
	Lower respiratory infections	179·3 (150·6–214·4)	176·6 (159·2–192·4)	2·7 (-21·7–37·5)	1·6 (-12·5–21·8)	282·5 (229·0–319·4)	297·9 (271·1–320·6)	-15·4 (-60·6–33·7)	-5·1 (-20·6–11·8)
	Other musculoskeletal disorders	203·9 (140·3–285·6)	126·6 (82·4–187·1)	77·3 (58·0–101·7)	62·1 (52·0–74·7)	169·9 (119·1–239·4)	99·1 (62·5–149·8)	70·8 (54·3–90·3)	73·3 (57·7–92·9)
	Road injuries	334·7 (303·2–367·3)	1168·0 (1088·7–1246·9)	-833·3 (-904·6–-766·5)	-71·3 (-73·5–-69·0)	588·7 (551·3–631·4)	1828·6 (1737·8–1922·4)	-1240·0 (-1324·0–-1142·9)	-67·8 (-69·7–-64·9)
	Stroke	111·8 (98·7–124·4)	121·3 (109·2–132·3)	-9·5 (-21·3–1·7)	-7·8 (-16·8–1·5)	179·3 (158·2–200·6)	190·5 (173·6–208·2)	-11·2 (-32·2–12·8)	-5·8 (-16·2–7·3)

	Tracheal, bronchus, and lung cancer	6·2 (5·5–7·0)	8·9 (7·8–10·1)	-2·7 (-4·0–-1·3)	-30·2 (-40·8–-15·3)	8·7 (7·6–10·1)	15·5 (13·8–17·8)	-6·8 (-9·4–-4·5)	-43·4 (-53·9–-31·8)
	Tuberculosis	257·3 (228·8–290·3)	254·9 (217·2–297·8)	2·4 (-35·5–42·6)	1·3 (-12·3–18·6)	649·0 (577·6–721·3)	553·0 (419·2–657·4)	96·0 (-11·0–228·1)	18·4 (-1·9–54·1)
25-49 years old	Age-related and other hearing loss	326·1 (213·6–472·5)	367·3 (233·9–542·7)	-41·2 (-70·6–-20·6)	-11·0 (-13·9–-8·2)	295·1 (193·4–424·7)	338·0 (214·7–497·5)	-42·9 (-71·7–-21·9)	-12·5 (-15·2–-9·4)
	Alzheimer's disease and other dementias	7·7 (3·9–15·6)	6·5 (3·2–13·4)	1·3 (-0·2–3·1)	21·1 (-3·6–57·7)	6·3 (3·2–12·7)	5·3 (2·6–10·8)	1·0 (-0·2–2·5)	20·3 (-3·8–55·6)
	Anxiety disorders	848·3 (564·7–1195·9)	512·7 (340·7–730·6)	335·6 (219·4–478·5)	65·7 (55·3–76·6)	704·5 (472·3–991·7)	425·6 (282·9–605·8)	278·9 (184·1–400·5)	65·7 (56·0–75·3)
	COVID-19	1544·3 (1322·9–1964·8)	2743·1 (2561·3–3030·6)	-1198·8 (-1353·8–-1017·6)	-43·8 (-49·4–-35·0)	NA	NA	NA	NA
	Chronic kidney disease	303·3 (270·4–340·3)	384·7 (344·3–429·3)	-81·4 (-112·8–-43·8)	-21·0 (-28·2–-12·3)	289·3 (259·3–325·2)	331·2 (285·4–380·4)	-41·8 (-81·5–2·1)	-12·4 (-22·4–0·6)
	Chronic obstructive pulmonary disease	165·2 (144·5–187·0)	215·5 (188·6–237·5)	-50·4 (-75·9–-12·9)	-23·2 (-33·8–-6·6)	244·0 (190·3–282·4)	311·8 (264·0–349·5)	-67·8 (-136·4–-19·4)	-21·5 (-40·8–-7·0)
	Cirrhosis and other chronic liver diseases	312·5 (279·6–350·3)	921·3 (842·3–1037·4)	-608·8 (-720·0–-527·4)	-66·0 (-70·5–-61·7)	415·4 (366·4–466·3)	1175·4 (1062·6–1341·3)	-760·0 (-917·4–-631·4)	-64·5 (-69·1–-58·6)
	Depressive disorders	1152·9 (775·3–1590·7)	759·0 (511·5–1057·6)	393·9 (262·0–552·3)	52·0 (47·1–56·4)	1033·0 (697·1–1438·2)	656·1 (442·1–913·1)	377·0 (258·0–519·4)	57·5 (54·2–61·0)
	Diabetes mellitus	523·3 (409·2–661·9)	631·1 (497·4–797·9)	-107·7 (-138·0–-79·4)	-17·1 (-20·0–-14·2)	321·0 (268·1–384·7)	369·1 (306·5–444·8)	-48·1 (-65·7–-27·8)	-13·0 (-16·8–-7·9)
	Falls	263·8 (196·8–350·1)	587·0 (485·0–713·7)	-323·2 (-377·1–-276·6)	-55·2 (-61·0–-49·3)	314·1 (236·4–416·5)	738·7 (615·0–896·5)	-424·6 (-502·6–-366·0)	-57·6 (-62·4–-52·8)
	HIV/AIDS	1008·8 (896·2–1165·5)	892·5 (812·7–1014·6)	116·3 (11·1–236·7)	13·1 (1·2–26·7)	581·2 (392·7–842·1)	689·8 (542·8–902·5)	-108·6 (-163·9–-30·5)	-16·4 (-28·0–-3·7)
	Headache disorders	1065·8 (204·8–2220·8)	662·3 (148·8–1376·5)	403·6 (53·1–892·4)	59·2 (34·1–79·2)	1072·1 (202·1–2250·5)	641·6 (145·1–1324·0)	430·5 (55·0–960·5)	65·1 (37·0–88·5)
	Ischemic heart disease	588·9 (538·9–644·3)	1496·2 (1393·2–1608·2)	-907·3 (-1021·2–-809·7)	-60·6 (-64·9–-56·4)	710·0 (642·2–777·3)	1604·1 (1507·4–1696·7)	-894·2 (-1004·3–-769·4)	-55·7 (-60·7–-50·7)
	Low back pain	1175·7 (806·2–1625·4)	745·6 (506·5–1024·4)	430·2 (297·5–602·5)	57·8 (53·7–62·6)	1284·5 (877·8–1778·9)	837·2 (569·5–1145·4)	447·3 (308·0–623·8)	53·5 (49·3–58·3)
	Lower respiratory infections	186·6 (159·9–218·9)	307·7 (285·9–332·6)	-121·1 (-143·7–-92·0)	-39·3 (-46·7–-30·2)	267·0 (229·5–304·6)	380·2 (354·8–403·5)	-113·2 (-152·1–-71·0)	-29·7 (-39·7–-19·4)
	Other musculoskeletal disorders	922·1 (644·3–1331·5)	624·5 (416·9–922·7)	297·6 (217·7–404·0)	48·2 (42·2–54·6)	746·1 (521·7–1066·6)	492·0 (323·9–726·9)	254·0 (188·9–341·1)	52·3 (45·3–60·3)
	Road injuries	396·2 (357·8–431·5)	1698·1 (1591·4–1818·2)	-1301·8 (-1405·6–-1202·7)	-76·7 (-78·7–-74·8)	646·4 (596·9–708·9)	2298·7 (2179·4–2416·9)	-1652·4 (-1749·9–-1534·6)	-71·9 (-73·8–-69·5)
	Stroke	561·0 (510·1–613·2)	868·2 (788·5–954·5)	-307·3 (-381·1–-224·5)	-35·3 (-41·4–-27·9)	894·9 (823·9–969·3)	1133·9 (1039·6–1217·0)	-239·0 (-344·7–-129·3)	-21·0 (-28·5–-12·3)
	Tracheal, bronchus, and lung cancer	119·4 (105·2–136·2)	215·3 (185·9–248·0)	-95·9 (-129·3–-66·6)	-44·3 (-53·0–-34·6)	144·9 (130·9–162·0)	343·7 (318·0–375·8)	-198·8 (-237·1–-165·9)	-57·7 (-63·4–-51·3)

	Tuberculosis	430·6 (384·6–484·5)	795·5 (708·7–926·9)	-364·8 (-484·3–-284·5)	-45·7 (-53·6–-38·3)	1150·3 (1047·8–1268·2)	1717·5 (1348·8–1999·3)	-567·2 (-852·2–-198·1)	-32·5 (-42·8–-14·7)
50-69 years old	Age-related and other hearing loss	1117·1 (731·8–1639·5)	1249·6 (810·6–1842·2)	-132·5 (-209·6–-75·2)	-10·5 (-12·5–-8·6)	1037·1 (679·2–1529·2)	1182·0 (766·1–1750·8)	-144·9 (-236·9–-84·4)	-12·2 (-14·1–-10·1)
	Alzheimer's disease and other dementias	526·8 (242·3–1188·1)	403·8 (185·2–940·2)	122·9 (54·4–255·4)	31·4 (18·3–42·4)	510·3 (235·4–1159·9)	380·2 (171·0–913·3)	130·2 (59·3–283·1)	35·1 (25·4–45·9)
	Anxiety disorders	735·7 (498·8–1031·8)	448·8 (297·0–638·8)	286·9 (184·7–413·7)	64·5 (48·2–81·5)	657·6 (449·1–929·2)	403·9 (266·6–577·8)	253·7 (161·4–372·6)	63·3 (46·2–79·8)
	COVID-19	3964·5 (3695·7–4359·9)	7780·6 (7323·5–8367·6)	-3816·2 (-4174·2–-3477·1)	-49·0 (-51·5–-46·1)	NA	NA	NA	NA
	Chronic kidney disease	1036·9 (936·6–1149·0)	1298·0 (1162·8–1449·2)	-261·1 (-374·4–-135·3)	-20·0 (-27·2–-11·1)	909·8 (820·4–1014·1)	1114·9 (971·7–1293·6)	-205·1 (-356·7–-54·4)	-18·1 (-28·3–-5·5)
	Chronic obstructive pulmonary disease	1576·1 (1395·2–1779·5)	2412·6 (2141·6–2635·7)	-836·5 (-1122·1–-453·8)	-34·5 (-44·0–-20·6)	2687·3 (2129·9–3077·2)	4434·6 (3871·0–4910·8)	-1747·3 (-2534·2–-1147·1)	-39·2 (-53·5–-27·9)
	Cirrhosis and other chronic liver diseases	818·2 (737·2–918·9)	1974·9 (1805·2–2180·7)	-1156·7 (-1376·0–-995·7)	-58·5 (-63·7–-52·9)	1308·6 (1148·7–1475·9)	2899·5 (2616·9–3304·0)	-1590·9 (-1942·7–-1251·6)	-54·7 (-60·3–-47·1)
	Depressive disorders	1233·6 (838·4–1670·7)	820·3 (554·8–1126·3)	413·3 (278·0–561·5)	50·4 (46·2–54·3)	1130·4 (765·7–1540·2)	743·0 (501·5–1010·3)	387·5 (261·2–530·1)	52·2 (48·9–55·5)
	Diabetes mellitus	2497·0 (2114·9–3030·4)	2776·0 (2327·6–3374·8)	-278·9 (-399·1–-166·3)	-10·0 (-13·7–-6·4)	1880·2 (1662·1–2184·2)	1930·3 (1689·2–2234·9)	-50·1 (-154·5–64·3)	-2·5 (-7·9–3·5)
	Falls	728·2 (578·2–924·4)	1098·1 (897·8–1346·1)	-369·9 (-460·3–-292·2)	-33·8 (-39·6–-28·3)	806·4 (642·4–1006·2)	1292·5 (1073·1–1559·7)	-486·1 (-605·3–-394·9)	-37·7 (-44·1–-32·7)
	HIV/AIDS	388·8 (313·1–494·2)	491·2 (408·8–600·2)	-102·4 (-216·5–11·9)	-20·4 (-38·5–2·7)	182·1 (125·3–243·1)	212·7 (162·8–271·4)	-30·6 (-57·0–3·8)	-14·5 (-27·2–1·8)
	Headache disorders	846·9 (232·3–1721·9)	522·4 (169·0–1046·0)	324·5 (61·3–702·8)	60·0 (35·1–82·2)	864·9 (257·1–1723·7)	520·7 (176·3–1034·6)	344·2 (79·8–715·5)	64·5 (41·9–85·6)
	Ischemic heart disease	3574·3 (3317·8–3831·6)	7477·8 (6976·2–8025·8)	-3903·5 (-4448·3–-3421·3)	-52·2 (-56·1–-47·9)	5158·0 (4883·1–5455·0)	10085·2 (9694·5–10510·5)	-4927·2 (-5412·1–-4395·6)	-48·8 (-52·1–-45·3)
	Low back pain	2161·5 (1479·4–3004·4)	1313·3 (902·2–1838·3)	848·3 (580·2–1165·8)	64·7 (60·4–69·4)	2432·6 (1659·7–3388·1)	1506·6 (1029·5–2116·9)	926·0 (632·0–1278·4)	61·6 (56·5–66·8)
	Lower respiratory infections	600·6 (511·0–669·5)	923·3 (860·3–986·3)	-322·7 (-409·5–-251·9)	-34·9 (-43·1–-27·6)	832·2 (705·0–957·4)	1300·6 (1212·8–1383·7)	-468·4 (-597·5–-328·4)	-36·0 (-45·5–-25·4)
	Other musculoskeletal disorders	1355·8 (918·0–1892·5)	879·7 (576·8–1267·4)	476·1 (342·7–633·9)	54·6 (47·8–62·4)	1103·3 (741·7–1552·5)	673·6 (433·1–981·8)	429·8 (306·4–577·0)	64·5 (56·3–73·7)
	Road injuries	498·4 (440·6–556·7)	1343·3 (1217·6–1482·5)	-844·9 (-958·1–-744·5)	-62·9 (-66·5–-59·4)	760·8 (686·1–848·1)	1809·8 (1665·9–1974·5)	-1049·0 (-1150·8–-952·4)	-58·0 (-60·6–-54·9)

	Stroke	3638·3 (3321·7–3951·7)	5466·0 (4947·6–6048·4)	-1827·6 (-2416·9–-1276·7)	-33·3 (-40·7–-24·9)	6509·2 (6035·3–7030·0)	8489·4 (7797·6–9150·9)	-1980·2 (-2791·6–-1209·6)	-23·2 (-30·6–-15·2)
	Tracheal, bronchus, and lung cancer	1062·9 (952·8–1178·0)	2506·7 (2188·8–2856·7)	-1443·7 (-1798·4–-1135·0)	-57·4 (-63·8–-50·6)	1134·4 (1057·3–1224·4)	3984·3 (3740·5–4275·8)	-2849·8 (-3146·6–-2598·4)	-71·5 (-74·3–-68·7)
	Tuberculosis	630·9 (566·1–710·3)	1280·1 (1133·7–1510·0)	-649·1 (-870·9–-503·3)	-50·5 (-58·3–-43·1)	1890·3 (1698·5–2100·7)	3759·4 (2948·5–4358·7)	-1869·0 (-2490·2–-1087·2)	-49·3 (-58·1–-36·6)
70+ years old	Age-related and other hearing loss	2872·9 (2064·5–3887·8)	2974·3 (2115·2–4052·5)	-101·4 (-199·0–-19·8)	-3·3 (-5·5–-0·8)	2697·9 (1922·9–3700·3)	2874·2 (2044·0–3963·0)	-176·4 (-285·8–-84·6)	-6·1 (-8·3–-3·5)
	Alzheimer's disease and other dementias	7164·8 (3395·8–14885·3)	4409·5 (2050·9–9804·8)	2755·2 (1350·4–5059·0)	64·4 (50·7–74·5)	6112·3 (2884·3–13270·4)	3796·5 (1769·1–8687·0)	2315·8 (1108·2–4604·7)	62·2 (52·8–70·6)
	Anxiety disorders	599·7 (413·8–846·5)	355·2 (239·6–495·3)	244·4 (152·3–364·7)	69·1 (50·5–85·5)	577·0 (399·8–817·6)	336·5 (228·7–466·8)	240·5 (147·7–359·2)	71·8 (52·1–89·6)
	COVID-19	8847·2 (8392·2–9392·4)	15070·3 (14331·7–15987·1)	-6223·1 (-6728·2–-5753·7)	-41·3 (-43·1–-39·4)	NA	NA	NA	NA
	Chronic kidney disease	2746·9 (2434·8–3040·1)	3402·4 (3084·5–3712·5)	-655·5 (-890·6–-439·5)	-19·2 (-26·0–-13·3)	2072·4 (1860·8–2313·6)	2813·1 (2500·7–3295·4)	-740·7 (-1183·0–-446·9)	-26·1 (-36·8–-17·0)
	Chronic obstructive pulmonary disease	7453·2 (6435·3–8535·3)	11398·4 (10077·5–12530·0)	-3945·2 (-5301·9–-2092·7)	-34·4 (-44·0–-20·8)	10578·1 (8842·1–11720·8)	17630·5 (15886·7–19252·0)	-7052·4 (-9519·8–-5015·3)	-39·9 (-50·8–-30·8)
	Cirrhosis and other chronic liver diseases	991·5 (867·6–1140·5)	1674·9 (1484·9–1862·6)	-683·5 (-885·8–-516·9)	-40·7 (-48·8–-32·1)	1545·6 (1308·7–1817·0)	2421·6 (2170·3–2801·3)	-876·0 (-1126·4–-537·4)	-36·1 (-44·7–-23·7)
	Depressive disorders	1058·9 (724·4–1435·7)	768·0 (523·2–1054·1)	290·8 (202·8–394·7)	37·9 (34·6–41·5)	1023·4 (694·5–1389·9)	740·9 (501·8–1005·8)	282·5 (192·6–386·7)	38·2 (35·0–41·7)
	Diabetes mellitus	4527·4 (3905·3–5279·2)	4992·7 (4311·5–5821·6)	-465·3 (-714·7–-218·5)	-9·3 (-13·7–-4·8)	3483·5 (3133·0–3890·6)	3722·5 (3329·2–4196·3)	-239·0 (-434·7–-40·6)	-6·4 (-10·9–-1·1)
	Falls	2870·3 (2336·8–3492·0)	2525·4 (2112·9–2956·2)	344·9 (110·7–588·6)	13·6 (4·2–23·4)	2666·5 (2204·4–3209·6)	2350·8 (2001·4–2792·5)	315·7 (61·6–540·9)	13·4 (2·6–22·9)
	HIV/AIDS	61·0 (49·1–75·6)	102·7 (84·9–126·8)	-41·7 (-58·8–-26·3)	-40·3 (-50·2–-28·5)	29·6 (20·5–39·6)	56·2 (39·9–76·2)	-26·5 (-39·0–-16·2)	-47·0 (-55·5–-36·8)
	Headache disorders	429·5 (138·8–876·1)	287·2 (106·3–580·5)	142·3 (31·9–317·2)	48·2 (27·3–67·6)	439·5 (150·4–879·2)	288·6 (108·1–581·8)	150·9 (41·1–318·0)	51·6 (31·9–69·7)
	Ischemic heart disease	13892·9 (12117·7–15074·8)	18667·3 (17266·6–19945·8)	-4774·4 (-6075·0–-3604·0)	-25·6 (-31·9–-19·9)	20919·7 (19014·2–21999·5)	25255·9 (23956·6–26259·5)	-4336·3 (-5377·4–-3238·0)	-17·2 (-21·9–-13·1)
	Low back pain	2704·1 (1896·6–3613·1)	1710·9 (1188·6–2296·0)	993·2 (700·8–1317·3)	58·3 (52·2–64·3)	3048·8 (2121·8–4121·8)	1900·1 (1300·8–2579·2)	1148·6 (804·1–1535·5)	60·7 (53·1–68·4)

	Lower respiratory infections	2504·3 (2109·5–2841·9)	3461·2 (3188·7–3707·2)	-956·9 (-1266·1–-669·9)	-27·7 (-36·3–-19·7)	3400·1 (2957·7–3879·9)	5025·5 (4685·1–5285·6)	-1625·5 (-1990·6–-1157·7)	-32·3 (-39·8–-22·9)
	Other musculoskeletal disorders	1084·6 (753·2–1559·2)	846·8 (581·1–1260·8)	237·8 (166·4–326·7)	28·5 (21·8–35·4)	860·1 (578·6–1290·9)	657·6 (429·7–1020·6)	202·5 (136·5–287·7)	31·2 (24·2–38·4)
	Road injuries	457·1 (400·1–521·2)	1052·2 (948·5–1172·4)	-595·1 (-666·5–-532·2)	-56·6 (-59·9–-53·6)	728·3 (639·9–831·6)	1468·4 (1319·3–1632·7)	-740·1 (-819·7–-667·3)	-50·4 (-52·9–-47·6)
	Stroke	13130·4 (11561·7–14390·3)	16531·4 (14887·2–18087·8)	-3401·0 (-5081·1–-1920·9)	-20·5 (-29·4–-11·8)	21727·9 (19911·6–23369·5)	24151·8 (22475·5–25693·6)	-2424·0 (-4236·0–-541·5)	-10·0 (-16·9–-2·4)
	Tracheal, bronchus, and lung cancer	2036·2 (1762·8–2271·0)	4937·3 (4340·6–5524·9)	-2901·2 (-3511·2–-2341·8)	-58·6 (-64·6–-52·6)	1655·6 (1514·1–1769·6)	5801·1 (5479·9–6151·8)	-4145·5 (-4502·6–-3855·5)	-71·4 (-73·7–-69·3)
	Tuberculosis	829·5 (747·8–918·7)	1580·2 (1400·0–1883·0)	-750·6 (-1054·6–-580·3)	-47·3 (-56·0–-39·2)	2147·6 (1892·4–2434·2)	4717·5 (3623·6–5724·3)	-2569·9 (-3637·6–-1575·1)	-54·0 (-64·1–-42·5)

The health outcomes presented here reflect the top 20 causes of disease burden, measured by Disability-Adjusted Life Years (DALYs), observed across females and males for the age group of 10 years and older globally in 2021. Health conditions are ordered alphabetically for each age group. The absolute differences between females and males were calculated as the DALY rate for females minus the rate for males for each specific cause and year, with a positive value indicating a higher rate for females compared to males. The relative gap was computed as a relative percent difference, with a positive value indicating higher values among females relative to males. Cell colours denote whether the absolute and relative gaps in DALY rates indicate that the cause disproportionately affects females (red) or males (blue).

DALYs = Disability-Adjusted Life Years. 95% UI = 95% uncertainty interval. COVID = Coronavirus disease. HIV = Human Immunodeficiency Virus. AIDS = Acquired Immunodeficiency Syndrome.

Table S7. Location-specific Disability-Adjusted Life Year (DALY) rates (per 100,000 population) for females and males and the absolute and relative gaps between females and males for the top 20 global causes by year, age, and super-region

			**2021**				**1990**			
**Location**	**Age group**	**Cause**	**DALYs per 100,000 among Females (95% UI)**	**DALYs per 100,000 among Males (95% UI)**	**Absolute gap in DALYs per 100,000**	**Relative % gap in DALYs per 100,000**	**DALYs per 100,000 among Females (95% UI)**	**DALYs per 100,000 among Males (95% UI)**	**Absolute gap in DALYs per 100,000**	**Relative % gap in DALYs per 100,000**
Central Europe, Eastern Europe, and Central Asia	10-24 years old	Age-related and other hearing loss	116·2 (73·8–170·3)	131·1 (83·5–190·0)	-14·8 (-25·9–-7·7)	-11·3 (-16·0–-6·8)	116·5 (73·7–171·0)	131·4 (83·4–191·5)	-15·0 (-24·9–-7·6)	-11·3 (-15·9–-7·4)
		Alzheimer's disease and other dementias	0·0 (0·0–0·0)	0·0 (0·0–0·0)	0·0 (0·0–0·0)	NA (NA–NA)	0·0 (0·0–0·0)	0·0 (0·0–0·0)	0·0 (0·0–0·0)	NA (NA–NA)
		Anxiety disorders	716·1 (450·2–1051·4)	426·3 (267·6–632·0)	289·8 (182·4–421·5)	68·2 (59·3–76·1)	521·6 (323·8–768·6)	315·3 (195·5–464·9)	206·3 (127·8–299·1)	65·6 (57·0–74·1)
		COVID-19	521·9 (343·5–920·5)	460·7 (319·7–755·0)	61·2 (-33·0–163·7)	12·9 (-6·9–31·3)	NA	NA	NA	NA
		Chronic kidney disease	70·8 (60·0–85·2)	73·0 (60·6–88·9)	-2·2 (-7·6–4·4)	-2·9 (-9·8–6·8)	75·0 (65·8–87·2)	83·9 (73·3–98·7)	-8·9 (-12·7–-5·3)	-10·5 (-13·9–-6·6)
		Chronic obstructive pulmonary disease	17·8 (15·0–20·8)	16·8 (14·5–19·3)	1·1 (-1·2–3·4)	6·5 (-6·9–21·2)	25·9 (22·7–28·8)	27·0 (24·6–29·7)	-1·2 (-3·9–1·7)	-4·3 (-13·8–6·4)
		Cirrhosis and other chronic liver diseases	61·1 (55·0–69·4)	79·9 (73·5–88·4)	-18·8 (-25·6–-13·0)	-23·5 (-30·2–-16·8)	80·2 (76·4–84·8)	86·3 (82·4–91·0)	-6·1 (-10·0–-1·9)	-7·0 (-11·6–-2·2)
		Depressive disorders	578·0 (361·7–849·7)	437·2 (279·2–642·2)	140·8 (82·2–222·1)	32·1 (25·7–38·7)	439·6 (278·5–644·4)	324·7 (209·4–473·3)	114·9 (68·5–173·4)	35·3 (30·2–40·6)
		Diabetes mellitus	54·8 (47·1–64·0)	44·2 (37·3–52·9)	10·6 (8·2–13·2)	24·1 (18·0–30·6)	49·3 (44·5–56·2)	35·0 (30·6–41·4)	14·4 (13·2–15·7)	41·3 (35·1–47·0)
		Falls	186·4 (137·3–253·8)	332·3 (273·6–411·3)	-145·9 (-168·4–-127·1)	-44·2 (-50·5–-37·7)	256·1 (192·7–339·6)	562·8 (473·4–679·4)	-306·7 (-346·5–-276·9)	-54·7 (-59·5–-49·5)
		HIV/AIDS	60·2 (57·8–63·3)	47·8 (45·9–50·4)	12·3 (11·7–13·3)	25·8 (24·2–27·2)	18·9 (18·7–19·2)	25·2 (24·7–26·0)	-6·3 (-6·9–-6·0)	-25·0 (-26·5–-24·2)
		Headache disorders	773·9 (128·6–1785·8)	445·3 (73·3–1014·0)	328·6 (52·6–779·1)	74·4 (41·0–90·8)	780·8 (133·9–1793·3)	445·8 (76·5–1017·2)	335·0 (54·3–786·4)	75·8 (42·0–91·6)
		Ischemic heart disease	34·0 (29·9–38·4)	71·9 (65·1–78·7)	-38·0 (-44·0–-31·7)	-52·7 (-57·9–-46·3)	49·0 (46·9–51·1)	98·9 (95·1–102·6)	-50·0 (-53·6–-46·1)	-50·5 (-52·5–-47·9)
		Low back pain	703·9 (459·6–998·3)	469·0 (303·1–658·7)	234·9 (151·5–335·7)	50·2 (44·3–56·8)	742·9 (487·6–1052·4)	482·1 (315·4–679·8)	260·9 (171·5–374·2)	54·2 (47·4–61·5)
		Lower respiratory infections	109·3 (100·1–118·7)	142·9 (132·4–154·7)	-33·6 (-42·8–-24·5)	-23·5 (-28·6–-17·6)	171·7 (166·1–178·2)	198·7 (191·3–205·9)	-27·0 (-34·1–-19·0)	-13·5 (-17·0–-9·9)

		Other musculoskeletal disorders	135·4 (89·4–199·7)	86·3 (52·1–136·7)	49·1 (35·2–65·8)	58·7 (42·6–79·4)	102·4 (65·9–155·1)	42·3 (21·1–79·0)	60·1 (42·9–82·0)	152·7 (92·2–231·7)
		Road injuries	270·7 (252·6–289·0)	803·5 (746·4–850·3)	-532·8 (-577·6–-479·1)	-66·3 (-68·6–-63·5)	656·9 (635·2–680·4)	2282·9 (2242·8–2329·1)	-1626·0 (-1658·0–-1594·6)	-71·2 (-71·9–-70·6)
		Stroke	75·0 (62·8–86·6)	72·5 (64·6–80·4)	2·4 (-4·5–8·9)	3·2 (-6·5–11·8)	128·4 (115·5–142·3)	123·3 (114·7–131·6)	5·1 (-1·8–13·0)	4·0 (-1·5–10·2)
		Tracheal, bronchus, and lung cancer	6·1 (5·4–7·0)	7·7 (7·1–8·3)	-1·6 (-2·3–-0·7)	-21·1 (-29·3–-9·1)	9·7 (9·2–10·2)	18·8 (18·1–19·6)	-9·1 (-9·9–-8·4)	-48·3 (-51·0–-45·3)
		Tuberculosis	34·6 (30·0–40·3)	42·2 (36·5–49·8)	-7·5 (-14·0–-2·9)	-17·7 (-29·6–-7·0)	71·6 (63·3–82·2)	89·9 (80·8–100·9)	-18·3 (-24·2–-13·9)	-20·4 (-25·7–-15·6)
	25-49 years old	Age-related and other hearing loss	284·6 (183·8–420·4)	310·7 (196·0–464·6)	-26·1 (-47·6–-12·1)	-8·3 (-11·3–-4·9)	273·0 (175·1–398·7)	298·5 (187·6–439·4)	-25·5 (-46·7–-11·8)	-8·4 (-11·4–-5·2)
		Alzheimer's disease and other dementias	8·2 (4·2–15·9)	6·7 (3·4–13·7)	1·5 (-0·3–3·6)	23·3 (-4·4–63·0)	6·2 (3·2–12·3)	5·0 (2·5–10·1)	1·2 (-0·0–2·8)	26·2 (-1·0–64·1)
		Anxiety disorders	710·7 (473·1–1004·2)	433·1 (280·9–616·7)	277·6 (185·0–396·0)	64·3 (56·6–72·5)	566·2 (379·4–794·0)	350·6 (231·9–501·3)	215·6 (145·0–302·0)	61·7 (53·3–70·5)
		COVID-19	2158·6 (1711·9–2824·8)	3671·3 (3054·7–4307·2)	-1512·8 (-2123·1–-897·8)	-41·0 (-54·3–-25·5)	NA	NA	NA	NA
		Chronic kidney disease	173·2 (149·6–206·4)	225·9 (201·1–257·9)	-52·7 (-66·5–-35·9)	-23·4 (-29·2–-16·2)	186·7 (165·8–209·8)	241·2 (221·1–265·3)	-54·6 (-61·0–-47·1)	-22·7 (-25·8–-19·3)
		Chronic obstructive pulmonary disease	93·4 (81·7–105·5)	157·5 (145·6–169·8)	-64·2 (-76·5–-51·2)	-40·7 (-47·3–-33·5)	124·7 (113·6–136·4)	234·3 (223·3–245·4)	-109·7 (-121·6–-99·3)	-46·8 (-51·3–-42·8)
		Cirrhosis and other chronic liver diseases	749·8 (678·0–829·5)	1664·0 (1544·1–1783·7)	-914·2 (-1052·8–-779·8)	-54·9 (-59·8–-49·1)	310·3 (302·7–317·9)	678·9 (661·9–696·9)	-368·6 (-388·1–-350·4)	-54·3 (-55·8–-52·7)
		Depressive disorders	1060·0 (716·9–1496·7)	740·0 (496·4–1032·4)	320·0 (204·3–457·7)	43·2 (38·0–48·2)	894·3 (604·1–1251·5)	625·4 (419·2–869·5)	268·9 (177·5–381·5)	43·0 (37·6–48·4)
		Diabetes mellitus	347·7 (261·8–452·5)	468·1 (365·9–589·2)	-120·4 (-148·8–-98·8)	-25·8 (-29·6–-22·0)	202·1 (162·8–248·2)	270·5 (222·5–328·5)	-68·4 (-82·2–-58·3)	-25·3 (-27·8–-22·6)
		Falls	420·6 (304·6–575·7)	1109·1 (887·9–1402·8)	-688·4 (-837·1–-577·1)	-62·3 (-66·0–-57·8)	505·9 (376·0–675·4)	1487·3 (1211·0–1827·9)	-981·5 (-1178·4–-842·7)	-66·1 (-69·5–-62·6)
		HIV/AIDS	573·0 (551·2–601·7)	1227·9 (1182·5–1285·3)	-654·9 (-686·9–-630·7)	-53·3 (-54·0–-52·4)	63·5 (62·6–65·0)	277·5 (272·6–286·3)	-214·0 (-221·3–-210·0)	-77·1 (-77·3–-77·0)
		Headache disorders	1198·0 (355·4–2456·8)	655·1 (212·3–1338·8)	542·9 (144·0–1137·7)	82·4 (52·7–98·4)	1195·9 (350·6–2456·1)	645·6 (203·8–1313·4)	550·3 (139·1–1167·3)	84·9 (56·4–102·1)

		Ischemic heart disease	474·9 (424·3–528·0)	2070·9 (1901·7–2236·7)	-1596·0 (-1763·9–-1449·8)	-77·0 (-79·6–-74·5)	624·3 (602·3–640·4)	3379·6 (3276·1–3464·9)	-2755·3 (-2830·2–-2666·6)	-81·5 (-81·9–-81·1)
		Low back pain	1710·2 (1174·1–2354·5)	1196·2 (820·7–1622·0)	514·0 (357·9–722·9)	43·1 (38·3–49·0)	1729·0 (1192·7–2388·8)	1180·4 (807·3–1606·3)	548·5 (376·5–778·1)	46·6 (41·4–52·8)
		Lower respiratory infections	186·9 (167·2–211·6)	563·3 (515·0–616·0)	-376·4 (-431·3–-324·5)	-66·8 (-71·2–-62·2)	151·4 (146·4–156·2)	367·4 (359·5–376·4)	-216·0 (-225·2–-207·4)	-58·8 (-60·4–-57·3)
		Other musculoskeletal disorders	320·4 (214·9–467·7)	88·0 (45·5–155·8)	232·4 (161·7–322·6)	282·9 (169·3–444·4)	238·6 (157·2–356·8)	26·4 (14·2–52·7)	212·2 (139·9–315·0)	877·6 (432·3–1333·9)
		Road injuries	327·9 (294·9–366·3)	1362·0 (1256·5–1471·8)	-1034·1 (-1118·4–-942·5)	-75·9 (-77·9–-73·7)	642·9 (597·6–696·2)	3381·7 (3261·3–3519·1)	-2738·8 (-2824·1–-2658·5)	-81·0 (-81·7–-80·2)
		Stroke	534·2 (478·1–589·1)	1008·5 (922·9–1085·1)	-474·3 (-545·1–-408·3)	-47·0 (-52·1–-42·1)	762·2 (714·1–808·2)	1167·4 (1118·2–1213·3)	-405·2 (-432·9–-374·6)	-34·7 (-37·1–-32·1)
		Tracheal, bronchus, and lung cancer	118·2 (108·2–129·1)	307·0 (282·8–331·6)	-188·8 (-213·6–-166·5)	-61·5 (-65·4–-57·6)	143·2 (138·4–147·6)	768·9 (745·9–792·4)	-625·7 (-648·9–-602·6)	-81·4 (-82·1–-80·6)
		Tuberculosis	94·7 (84·5–105·6)	305·1 (280·7–332·8)	-210·4 (-235·7–-188·5)	-68·9 (-72·5–-65·6)	133·8 (124·9–144·0)	541·6 (518·3–567·8)	-407·8 (-427·2–-390·2)	-75·3 (-76·5–-74·1)
	50-69 years old	Age-related and other hearing loss	1033·4 (670·8–1512·4)	1074·3 (692·5–1581·6)	-40·9 (-85·8–-6·1)	-3·7 (-6·8–-0·7)	1001·0 (651·3–1468·8)	1019·4 (659·6–1512·4)	-18·4 (-60·9–19·4)	-1·7 (-5·2–2·2)
		Alzheimer's disease and other dementias	514·8 (240·7–1166·9)	382·9 (176·7–883·7)	131·9 (60·7–278·0)	35·5 (24·8–47·3)	469·1 (221·0–1080·2)	330·4 (152·8–762·6)	138·6 (66·2–300·9)	42·8 (34·2–53·0)
		Anxiety disorders	647·4 (443·1–922·6)	374·9 (244·5–538·7)	272·5 (172·1–389·9)	73·2 (57·0–89·0)	553·1 (376·4–776·7)	328·5 (214·6–475·8)	224·6 (142·4–328·6)	68·8 (50·6–84·1)
		COVID-19	8051·6 (6961·5–9181·4)	14316·8 (12056·7–16532·6)	-6265·2 (-8436·6–-4157·8)	-43·5 (-53·4–-32·3)	NA	NA	NA	NA
		Chronic kidney disease	567·3 (484·4–676·3)	677·2 (594·7–772·5)	-109·8 (-152·9–-20·6)	-16·2 (-22·8–-3·2)	499·7 (444·5–564·0)	589·5 (538·8–648·0)	-89·7 (-109·4–-68·3)	-15·3 (-19·0–-11·2)
		Chronic obstructive pulmonary disease	619·2 (564·2–682·8)	1556·2 (1452·4–1671·8)	-937·0 (-1034·5–-840·8)	-60·2 (-63·6–-56·3)	1021·6 (962·4–1087·9)	3356·3 (3262·0–3453·4)	-2334·7 (-2424·9–-2247·7)	-69·6 (-71·2–-67·8)
		Cirrhosis and other chronic liver diseases	1419·0 (1314·3–1535·6)	2973·7 (2765·1–3185·1)	-1554·7 (-1793·4–-1342·8)	-52·2 (-56·8–-47·7)	981·7 (958·1–1003·0)	2219·0 (2163·3–2270·9)	-1237·4 (-1292·0–-1190·2)	-55·8 (-57·0–-54·6)
		Depressive disorders	1248·7 (841·3–1725·0)	764·4 (517·6–1062·4)	484·3 (315·6–679·4)	63·4 (55·9–70·2)	1222·0 (825·8–1681·8)	737·1 (497·9–993·7)	484·9 (320·9–689·1)	65·8 (57·9–74·2)

		Diabetes mellitus	2106·7 (1733·8–2614·7)	2271·0 (1876·7–2792·8)	-164·3 (-271·0–-71·9)	-7·2 (-11·0–-3·2)	1256·2 (1066·1–1510·6)	1239·9 (1041·6–1511·7)	16·3 (-22·4–50·8)	1·4 (-1·7–4·3)
		Falls	714·8 (524·1–946·6)	1780·6 (1420·3–2244·1)	-1065·9 (-1315·3–-882·1)	-60·0 (-63·1–-56·7)	879·4 (660·4–1144·5)	2063·4 (1645·6–2621·8)	-1184·0 (-1476·7–-976·5)	-57·5 (-60·1–-55·0)
		HIV/AIDS	92·9 (88·4–98·6)	186·9 (180·0–195·8)	-94·0 (-100·0–-88·9)	-50·3 (-51·9–-47·9)	18·5 (18·1–19·2)	55·9 (54·0–59·0)	-37·4 (-39·7–-35·9)	-66·8 (-67·4–-66·5)
		Headache disorders	1053·2 (455·2–1905·9)	624·1 (280·6–1173·1)	429·1 (165·9–808·5)	68·4 (50·6–82·4)	1084·4 (463·4–1935·4)	634·4 (285·3–1185·4)	450·0 (175·4–847·9)	70·6 (51·3–85·3)
		Ischemic heart disease	5585·7 (5082·1–6159·8)	15286·5 (13871·5–16729·3)	-9700·8 (-11177·6–-8450·4)	-63·4 (-67·4–-59·1)	7828·9 (7623·3–7973·9)	20466·9 (20028·8–20797·6)	-12638·0 (-12888·3–-12339·3)	-61·7 (-62·4–-61·1)
		Low back pain	3113·8 (2121·4–4356·9)	2148·1 (1472·7–2995·6)	965·7 (657·2–1371·4)	45·1 (39·1–51·1)	3228·8 (2207·9–4480·1)	2138·1 (1450·4–3004·2)	1090·7 (742·5–1508·8)	51·2 (44·2–58·0)
		Lower respiratory infections	311·9 (289·6–337·1)	1097·2 (1011·7–1185·4)	-785·3 (-871·2–-705·7)	-71·5 (-74·3–-68·6)	265·0 (256·1–272·6)	827·5 (808·4–846·0)	-562·5 (-583·7–-541·8)	-68·0 (-69·2–-66·9)
		Other musculoskeletal disorders	770·3 (514·1–1097·8)	84·1 (44·4–164·1)	686·2 (468·5–959·9)	877·4 (527·8–1306·2)	589·9 (380·8–872·1)	29·7 (22·4–44·6)	560·2 (356·9–827·2)	1889·1 (1459·9–2397·7)
		Road injuries	322·4 (281·8–372·6)	939·7 (831·3–1062·5)	-617·3 (-695·9–-543·1)	-65·7 (-67·7–-63·5)	674·5 (601·9–754·7)	2190·5 (2009·9–2393·8)	-1516·0 (-1641·3–-1413·3)	-69·2 (-70·3–-68·2)
		Stroke	3643·4 (3327·1–3931·6)	6850·9 (6345·0–7379·4)	-3207·5 (-3711·4–-2746·0)	-46·8 (-51·1–-42·0)	7161·0 (6896·7–7397·8)	10289·9 (10012·5–10538·2)	-3128·9 (-3290·7–-2973·7)	-30·4 (-31·8–-29·1)
		Tracheal, bronchus, and lung cancer	1038·1 (962·3–1113·4)	4578·1 (4258·5–4869·6)	-3540·0 (-3856·6–-3223·4)	-77·3 (-79·2–-75·3)	964·5 (935·0–988·6)	8469·5 (8300·2–8633·3)	-7505·0 (-7658·9–-7342·5)	-88·6 (-88·9–-88·3)
		Tuberculosis	75·1 (67·5–83·4)	413·1 (370·4–460·4)	-338·0 (-385·3–-296·5)	-81·8 (-84·0–-79·1)	154·4 (146·4–162·7)	1070·5 (1031·7–1113·5)	-916·1 (-953·7–-880·4)	-85·6 (-86·1–-85·0)
	70+ years old	Age-related and other hearing loss	2888·4 (2078·5–3942·6)	2761·1 (1975·7–3828·9)	127·3 (28·2–239·4)	4·7 (1·0–8·8)	2874·7 (2068·1–3982·1)	2803·8 (2004·1–3907·3)	70·9 (-21·7–189·5)	2·6 (-0·7–6·6)
		Alzheimer's disease and other dementias	6122·2 (2950·6–13001·2)	4032·4 (1899·6–9041·8)	2089·8 (1028·4–4176·4)	52·7 (43·6–60·7)	5291·8 (2554·8–11632·9)	3735·2 (1788·7–8481·9)	1556·6 (753·5–3110·7)	42·4 (35·8–49·1)
		Anxiety disorders	556·3 (378·7–792·8)	283·5 (190·9–398·7)	272·8 (174·0–404·7)	96·6 (74·8–118·0)	509·0 (348·6–723·6)	262·7 (177·1–372·0)	246·3 (156·1–365·0)	94·2 (71·8–116·1)
		COVID-19	19528·1 (17387·5–21841·7)	30780·8 (27128·1–34163·5)	-11252·7 (-14596·5–-7944·7)	-36·4 (-44·7–-27·4)	NA	NA	NA	NA

		Chronic kidney disease	1476·3 (1280·7–1680·3)	1725·5 (1547·9–1919·3)	-249·2 (-337·3–-133·7)	-14·5 (-19·9–-7·9)	997·3 (831·6–1178·9)	1404·4 (1243·4–1595·6)	-407·0 (-466·6–-359·0)	-29·1 (-34·1–-24·0)
		Chronic obstructive pulmonary disease	2016·9 (1823·6–2198·5)	4783·8 (4484·4–5071·7)	-2766·8 (-3022·7–-2516·9)	-57·8 (-60·9–-54·5)	4165·8 (3908·0–4382·0)	10530·3 (10134·8–10885·5)	-6364·6 (-6653·2–-6106·3)	-60·4 (-62·2–-58·8)
		Cirrhosis and other chronic liver diseases	784·0 (715·6–847·4)	1536·8 (1440·5–1629·3)	-752·8 (-840·6–-667·0)	-49·0 (-53·0–-45·0)	808·9 (773·0–837·3)	1663·5 (1616·0–1709·4)	-854·6 (-896·6–-811·3)	-51·4 (-53·2–-49·5)
		Depressive disorders	1169·3 (801·5–1609·7)	752·9 (518·5–1030·9)	416·4 (282·2–585·7)	55·4 (49·2–62·0)	1281·2 (862·0–1780·8)	782·3 (532·2–1081·1)	498·9 (339·5–715·1)	63·8 (57·0–71·7)
		Diabetes mellitus	3939·5 (3410·3–4645·7)	3775·1 (3210·2–4469·2)	164·4 (-21·1–337·0)	4·4 (-0·6–9·5)	1971·7 (1672·5–2325·7)	2079·6 (1736·0–2471·0)	-107·9 (-205·0–-33·6)	-5·1 (-9·0–-1·8)
		Falls	1634·2 (1292·6–2051·4)	2381·4 (1897·8–2984·6)	-747·2 (-949·9–-593·3)	-31·4 (-34·1–-28·5)	2103·1 (1713·8–2598·1)	2606·0 (2099·3–3248·4)	-502·9 (-717·0–-360·6)	-19·2 (-23·1–-15·6)
		HIV/AIDS	2·2 (1·8–2·8)	5·2 (4·3–6·1)	-3·0 (-3·8–-2·2)	-57·6 (-64·0–-46·7)	0·2 (0·2–0·2)	0·8 (0·8–0·9)	-0·6 (-0·7–-0·5)	-72·0 (-73·0–-71·2)
		Headache disorders	504·7 (214·7–969·5)	353·0 (146·6–670·0)	151·7 (62·2–300·3)	43·1 (28·6–54·3)	515·9 (215·8–991·9)	349·9 (146·4–660·1)	165·9 (64·1–320·7)	47·6 (32·6–59·7)
		Ischemic heart disease	31714·0 (28375·1–34856·8)	39542·8 (36601·0–42605·6)	-7828·8 (-11291·6–-4519·3)	-19·7 (-27·7–-11·6)	42439·7 (39757·1–43627·0)	56577·2 (54640·3–57657·9)	-14137·5 (-15010·2–-13493·1)	-25·0 (-27·3–-23·8)
		Low back pain	4025·0 (2856·0–5441·5)	2856·0 (1965·5–3873·7)	1169·0 (801·4–1595·6)	41·3 (30·6–51·4)	4227·5 (2953·5–5680·6)	2960·3 (2036·1–4040·0)	1267·2 (885·2–1725·5)	43·1 (32·3–52·9)
		Lower respiratory infections	901·8 (807·6–970·4)	1844·8 (1713·7–1956·1)	-943·0 (-1026·7–-847·0)	-51·1 (-54·5–-47·6)	812·9 (755·3–850·1)	1669·4 (1596·5–1730·2)	-856·4 (-916·9–-801·2)	-51·3 (-53·9–-49·0)
		Other musculoskeletal disorders	600·6 (401·0–899·2)	77·9 (60·6–135·9)	522·7 (337·4–780·3)	677·3 (463·5–877·5)	393·0 (222·1–649·1)	31·1 (25·6–43·4)	361·9 (195·8–603·3)	1146·3 (743·7–1564·2)
		Road injuries	291·6 (249·1–344·5)	646·8 (552·3–755·4)	-355·2 (-417·8–-302·0)	-54·9 (-56·6–-53·1)	665·5 (588·5–762·6)	1447·7 (1282·7–1647·2)	-782·2 (-888·6–-696·0)	-54·0 (-55·1–-53·1)
		Stroke	18395·4 (16728·0–19695·1)	20411·8 (19137·9–21696·5)	-2016·4 (-3332·7–-721·5)	-9·9 (-16·2–-3·6)	33018·7 (31112·9–33979·5)	36552·8 (35423·7–37323·9)	-3534·1 (-4409·6–-2969·8)	-9·7 (-12·5–-8·1)
		Tracheal, bronchus, and lung cancer	1333·6 (1215·0–1423·7)	5810·5 (5469·6–6141·3)	-4476·9 (-4759·7–-4163·9)	-77·0 (-78·8–-75·3)	1137·6 (1081·1–1174·6)	7130·6 (6939·6–7300·2)	-5993·0 (-6142·9–-5833·8)	-84·0 (-84·6–-83·5)
		Tuberculosis	53·6 (48·7–58·1)	152·3 (141·1–163·8)	-98·7 (-109·0–-89·3)	-64·8 (-67·8–-61·8)	157·3 (148·4–166·7)	575·8 (549·8–601·5)	-418·4 (-441·5–-399·6)	-72·7 (-74·0–-71·6)

High-income	10-24 years old	Age-related and other hearing loss	72·3 (47·2–104·5)	78·9 (50·9–114·3)	-6·5 (-12·3–-2·3)	-8·3 (-13·4–-3·0)	72·0 (46·4–104·0)	76·8 (50·0–110·9)	-4·8 (-9·9–-1·1)	-6·3 (-11·9–-1·6)
		Alzheimer's disease and other dementias	0·0 (0·0–0·0)	0·0 (0·0–0·0)	0·0 (0·0–0·0)	NA (NA–NA)	0·0 (0·0–0·0)	0·0 (0·0–0·0)	0·0 (0·0–0·0)	NA (NA–NA)
		Anxiety disorders	1193·0 (752·1–1724·6)	669·8 (421·8–987·1)	523·2 (330·1–731·7)	78·3 (67·8–89·1)	899·6 (566·4–1312·7)	505·9 (317·6–746·7)	393·6 (252·6–564·5)	78·1 (67·6–88·5)
		COVID-19	247·0 (181·2–385·9)	469·4 (423·7–577·7)	-222·5 (-245·1–-187·3)	-47·9 (-57·2–-32·1)	NA	NA	NA	NA
		Chronic kidney disease	28·4 (22·0–36·3)	26·9 (21·9–33·4)	1·5 (-0·2–4·1)	5·5 (-0·7–13·2)	29·7 (24·1–36·7)	31·1 (26·4–37·3)	-1·4 (-3·1–0·6)	-4·7 (-10·1–1·9)
		Chronic obstructive pulmonary disease	13·1 (11·3–15·5)	13·8 (11·9–16·1)	-0·7 (-2·3–0·6)	-5·2 (-15·1–4·7)	16·0 (13·1–19·0)	18·6 (16·1–21·4)	-2·6 (-4·5–-0·7)	-14·0 (-23·9–-3·6)
		Cirrhosis and other chronic liver diseases	11·7 (11·0–12·9)	13·5 (12·6–14·5)	-1·7 (-2·3–-1·1)	-13·0 (-16·3–-8·6)	25·2 (23·7–26·9)	32·3 (30·2–34·3)	-7·2 (-9·2–-5·3)	-22·1 (-27·3–-17·2)
		Depressive disorders	1386·8 (908·6–2024·5)	706·2 (460·7–1038·2)	680·7 (452·7–978·7)	96·6 (87·0–105·1)	890·9 (583·3–1273·4)	483·0 (313·5–695·7)	407·9 (267·6–588·6)	84·6 (77·3–92·1)
		Diabetes mellitus	62·0 (44·1–87·2)	62·5 (45·6–86·0)	-0·5 (-2·3–2·2)	-0·9 (-4·4–2·9)	41·8 (33·3–53·6)	41·4 (33·2–52·1)	0·4 (-0·7–1·8)	1·0 (-1·6–4·0)
		Falls	154·4 (104·2–225·0)	218·8 (164·2–292·0)	-64·5 (-78·0–-54·6)	-29·9 (-37·2–-21·8)	183·5 (125·7–262·9)	334·9 (262·8–430·6)	-151·4 (-174·7–-132·6)	-45·6 (-52·5–-38·0)
		HIV/AIDS	9·9 (9·0–11·2)	14·8 (13·4–16·7)	-4·9 (-5·7–-4·3)	-32·9 (-35·4–-30·6)	25·6 (24·2–27·4)	73·4 (70·0–78·0)	-47·8 (-50·9–-45·7)	-65·2 (-65·9–-64·3)
		Headache disorders	970·0 (97·5–2269·7)	496·5 (58·4–1149·1)	473·5 (34·2–1122·7)	93·6 (47·7–134·6)	977·9 (94·9–2321·5)	496·8 (58·8–1153·6)	481·1 (34·8–1153·5)	95·2 (47·8–139·3)
		Ischemic heart disease	8·0 (7·6–8·4)	22·8 (22·0–23·8)	-14·8 (-15·8–-14·0)	-65·0 (-66·8–-63·1)	19·7 (18·8–20·8)	48·6 (46·7–50·7)	-28·9 (-30·8–-27·1)	-59·5 (-61·6–-57·4)
		Low back pain	755·8 (506·3–1042·0)	508·7 (340·7–702·9)	247·1 (166·4–352·3)	48·7 (44·6–53·6)	838·6 (553·4–1176·1)	554·7 (364·6–784·9)	283·8 (186·8–397·5)	51·3 (46·3–56·3)
		Lower respiratory infections	27·9 (26·6–29·3)	31·9 (30·5–33·5)	-4·1 (-5·8–-2·2)	-12·7 (-17·8–-7·2)	56·8 (53·1–59·2)	70·8 (68·2–73·3)	-14·0 (-18·5–-10·6)	-19·7 (-25·8–-15·3)
		Other musculoskeletal disorders	504·0 (348·7–694·3)	395·0 (269·0–557·5)	108·9 (75·7–146·9)	27·7 (24·0–31·5)	331·9 (218·4–486·4)	242·6 (151·1–367·2)	89·3 (64·7–124·6)	37·6 (29·4–47·1)
		Road injuries	272·5 (262·0–286·5)	686·7 (663·5–711·7)	-414·2 (-431·1–-397·8)	-60·3 (-61·6–-59·1)	786·6 (763·7–814·8)	2383·0 (2336·0–2430·6)	-1596·4 (-1625·2–-1564·9)	-67·0 (-67·6–-66·3)
		Stroke	50·5 (41·6–59·3)	47·1 (40·6–53·5)	3·3 (0·5–6·4)	6·9 (1·1–12·5)	98·3 (87·5–109·6)	104·7 (95·7–113·2)	-6·4 (-11·6–-1·7)	-6·2 (-11·7–-1·6)

		Tracheal, bronchus, and lung cancer	4·2 (3·9–4·5)	4·3 (4·1–4·6)	-0·2 (-0·4–0·1)	-3·5 (-9·4–2·5)	5·3 (5·1–5·6)	10·1 (9·7–10·5)	-4·8 (-5·3–-4·3)	-47·2 (-50·3–-43·7)
		Tuberculosis	4·1 (3·5–5·0)	3·6 (2·9–4·5)	0·5 (0·1–0·9)	14·3 (1·3–27·5)	23·0 (20·4–26·0)	21·7 (18·8–24·8)	1·3 (-1·9–4·3)	6·3 (-8·1–21·8)
	25-49 years old	Age-related and other hearing loss	176·1 (113·2–261·2)	235·6 (148·4–356·9)	-59·5 (-96·9–-34·7)	-25·1 (-28·1–-22·0)	172·1 (112·1–255·6)	227·6 (144·5–343·4)	-55·5 (-88·4–-31·8)	-24·2 (-27·4–-20·8)
		Alzheimer's disease and other dementias	9·1 (4·7–17·1)	7·3 (3·7–14·5)	1·7 (-0·1–4·1)	26·1 (-1·0–68·6)	8·1 (4·3–15·4)	6·3 (3·2–12·8)	1·8 (0·2–3·9)	30·1 (3·8–68·8)
		Anxiety disorders	1225·9 (799·2–1719·3)	664·4 (436·8–940·0)	561·5 (363·1–811·3)	84·8 (68·6–100·9)	983·3 (654·3–1396·6)	526·4 (351·7–746·0)	456·9 (298·6–658·0)	87·1 (70·9–102·8)
		COVID-19	812·7 (715·0–1016·0)	1619·3 (1562·0–1732·9)	-806·6 (-859·2–-721·1)	-49·9 (-54·4–-41·4)	NA	NA	NA	NA
		Chronic kidney disease	148·2 (123·0–174·5)	180·4 (153·7–210·5)	-32·2 (-38·4–-25·5)	-17·9 (-21·1–-14·3)	118·1 (97·3–141·8)	152·9 (128·9–178·2)	-34·8 (-40·7–-29·2)	-22·8 (-25·8–-19·4)
		Chronic obstructive pulmonary disease	83·9 (75·4–93·5)	86·6 (77·8–95·6)	-2·7 (-11·7–5·9)	-3·0 (-12·6–7·0)	80·8 (70·5–91·8)	105·6 (95·2–115·8)	-24·8 (-34·4–-16·1)	-23·4 (-31·6–-16·1)
		Cirrhosis and other chronic liver diseases	180·8 (175·3–186·2)	371·3 (360·6–381·4)	-190·5 (-201·2–-179·9)	-51·3 (-53·1–-49·5)	252·5 (246·8–257·6)	731·7 (689·1–754·9)	-479·2 (-501·4–-439·7)	-65·5 (-66·7–-63·8)
		Depressive disorders	1454·2 (992·1–2020·7)	869·3 (592·0–1235·6)	585·0 (397·1–810·5)	67·4 (61·8–73·0)	1107·8 (761·7–1520·5)	694·2 (469·0–949·6)	413·6 (282·5–563·8)	59·7 (54·8–64·7)
		Diabetes mellitus	395·2 (285·4–523·2)	535·0 (398·6–697·6)	-139·8 (-174·2–-109·9)	-26·3 (-29·1–-23·3)	224·7 (180·3–277·5)	315·1 (260·7–384·9)	-90·4 (-107·1–-77·6)	-28·7 (-30·7–-26·7)
		Falls	337·3 (229·4–472·9)	514·3 (388·9–679·2)	-177·0 (-210·8–-148·7)	-34·7 (-40·1–-29·3)	364·6 (250·3–506·8)	733·4 (571·5–944·4)	-368·9 (-446·4–-309·8)	-50·6 (-55·8–-45·3)
		HIV/AIDS	65·7 (57·2–77·3)	149·1 (131·7–173·6)	-83·5 (-98·4–-73·7)	-56·0 (-58·4–-53·1)	137·5 (133·1–143·4)	958·8 (937·5–987·2)	-821·3 (-844·4–-804·4)	-85·7 (-85·8–-85·4)
		Headache disorders	1337·3 (278·2–2749·4)	682·5 (163·5–1417·0)	654·8 (103·7–1433·3)	94·8 (46·6–136·3)	1345·3 (277·4–2789·0)	676·3 (159·8–1392·4)	669·1 (104·1–1439·0)	97·8 (48·7–143·9)
		Ischemic heart disease	160·3 (155·2–166·0)	579·6 (564·2–594·3)	-419·3 (-433·3–-405·5)	-72·3 (-73·2–-71·4)	278·4 (272·1–284·6)	1223·2 (1206·1–1240·6)	-944·8 (-959·4–-929·4)	-77·2 (-77·7–-76·8)
		Low back pain	1631·6 (1156·0–2207·7)	1208·2 (849·7–1632·3)	423·4 (304·2–578·4)	35·1 (32·6–37·8)	1707·4 (1183·8–2334·4)	1278·1 (881·3–1750·7)	429·4 (301·0–588·4)	33·7 (31·1–36·2)

		Lower respiratory infections	61·5 (59·1–64·1)	89·5 (86·6–92·2)	-27·9 (-30·7–-25·4)	-31·2 (-33·9–-28·6)	92·8 (90·8–94·5)	171·7 (168·4–175·4)	-78·9 (-82·5–-75·4)	-45·9 (-47·4–-44·6)
		Other musculoskeletal disorders	1097·2 (761·9–1533·3)	767·0 (521·3–1090·8)	330·2 (236·2–445·9)	43·4 (38·4–48·8)	891·2 (612·2–1261·1)	556·8 (355·8–836·6)	334·4 (243·3–443·5)	61·1 (49·6–77·2)
		Road injuries	273·9 (247·0–310·2)	790·0 (735·1–857·5)	-516·1 (-551·0–-485·1)	-65·4 (-66·8–-63·6)	614·0 (561·1–682·2)	1909·9 (1797·6–2055·1)	-1295·8 (-1378·0–-1233·6)	-67·9 (-69·0–-66·6)
		Stroke	233·9 (210·7–257·5)	299·5 (279·0–320·8)	-65·6 (-72·8–-57·8)	-21·9 (-25·0–-18·9)	443·2 (416·0–470·4)	582·4 (554·4–610·0)	-139·2 (-157·3–-123·6)	-23·9 (-26·9–-21·4)
		Tracheal, bronchus, and lung cancer	126·3 (122·3–130·0)	170·7 (163·5–178·6)	-44·4 (-51·6–-37·8)	-26·0 (-29·2–-23·0)	198·1 (194·0–201·5)	461·4 (450·4–473·1)	-263·3 (-274·9–-252·2)	-57·1 (-58·3–-55·8)
		Tuberculosis	7·1 (6·6–7·7)	13·3 (12·4–14·3)	-6·2 (-6·9–-5·6)	-46·8 (-49·7–-43·7)	39·7 (37·0–42·5)	91·4 (84·0–99·7)	-51·6 (-59·5–-44·3)	-56·4 (-60·7–-52·3)
	50-69 years old	Age-related and other hearing loss	671·7 (433·9–981·6)	951·0 (608·7–1405·9)	-279·3 (-437·9–-172·3)	-29·3 (-32·8–-25·3)	685·4 (444·1–1002·0)	946·7 (605·1–1411·2)	-261·3 (-410·3–-156·3)	-27·5 (-30·8–-23·6)
		Alzheimer's disease and other dementias	531·3 (255·7–1162·9)	401·9 (187·3–938·7)	129·4 (64·6–242·3)	33·6 (24·6–44·7)	532·6 (251·9–1216·9)	383·7 (178·4–900·0)	148·9 (70·1–306·3)	39·8 (31·5–50·5)
		Anxiety disorders	951·4 (639·4–1330·0)	501·2 (334·4–731·3)	450·2 (293·1–650·7)	90·6 (69·2–112·3)	816·4 (549·4–1152·4)	436·0 (288·8–640·7)	380·4 (238·7–551·2)	88·0 (63·8–109·9)
		COVID-19	1934·1 (1845·5–2096·0)	3810·7 (3725·5–3911·1)	-1876·6 (-1942·2–-1785·4)	-49·3 (-51·0–-46·1)	NA	NA	NA	NA
		Chronic kidney disease	569·4 (504·7–631·7)	815·0 (739·3–894·8)	-245·6 (-274·3–-216·5)	-30·2 (-32·8–-27·3)	461·9 (407·6–516·2)	625·0 (558·7–693·9)	-163·1 (-186·5–-142·4)	-26·1 (-28·5–-23·9)
		Chronic obstructive pulmonary disease	1032·8 (966·7–1097·6)	1256·5 (1190·8–1322·9)	-223·7 (-281·2–-161·9)	-17·8 (-22·0–-13·1)	948·2 (882·6–1013·3)	1723·0 (1652·3–1797·6)	-774·7 (-833·7–-713·3)	-45·0 (-48·1–-41·9)
		Cirrhosis and other chronic liver diseases	548·2 (525·6–563·9)	1371·0 (1336·3–1401·9)	-822·8 (-848·0–-796·8)	-60·0 (-61·2–-59·0)	790·8 (764·1–810·2)	2201·1 (2144·1–2245·6)	-1410·3 (-1455·2–-1364·1)	-64·1 (-65·1–-63·1)
		Depressive disorders	1040·6 (710·3–1435·9)	647·9 (440·3–896·0)	392·7 (266·9–548·7)	60·7 (54·3–66·7)	905·8 (620·3–1236·4)	582·6 (400·9–785·7)	323·2 (220·3–444·4)	55·5 (50·4–61·0)
		Diabetes mellitus	1655·0 (1259·2–2207·5)	2392·4 (1880·6–3084·8)	-737·4 (-885·1–-611·2)	-30·9 (-33·5–-28·4)	1299·4 (1114·6–1565·0)	1642·7 (1396·2–1986·8)	-343·4 (-415·4–-277·9)	-20·9 (-22·4–-19·2)
		Falls	911·6 (657·1–1230·4)	1115·6 (877·6–1417·4)	-204·0 (-233·2–-170·3)	-18·6 (-24·9–-12·6)	851·1 (613·5–1143·4)	1287·6 (995·3–1653·3)	-436·5 (-520·5–-374·9)	-34·1 (-38·6–-29·8)

		HIV/AIDS	49·2 (40·4–61·3)	165·6 (138·5–199·3)	-116·4 (-140·9–-97·6)	-70·3 (-73·1–-67·3)	31·1 (29·8–32·9)	214·1 (207·2–223·4)	-183·0 (-190·4–-177·3)	-85·5 (-85·7–-85·2)
		Headache disorders	933·4 (262·0–1871·7)	499·8 (171·3–994·8)	433·6 (91·8–906·0)	85·0 (43·4–125·0)	930·8 (269·6–1848·5)	498·6 (172·6–993·3)	432·2 (99·3–900·3)	85·3 (45·4–124·6)
		Ischemic heart disease	1216·5 (1159·2–1259·8)	3926·7 (3821·4–4018·7)	-2710·2 (-2775·0–-2644·7)	-69·0 (-69·9–-68·2)	3456·9 (3350·3–3531·7)	10154·8 (9996·4–10281·6)	-6697·8 (-6785·5–-6610·3)	-66·0 (-66·7–-65·4)
		Low back pain	2489·9 (1720·7–3399·0)	1813·1 (1247·9–2486·2)	676·8 (466·2–916·0)	37·4 (34·3–40·3)	2604·8 (1776·1–3589·3)	1912·0 (1306·3–2680·1)	692·7 (470·9–934·6)	36·3 (32·7–39·8)
		Lower respiratory infections	223·6 (211·7–233·7)	412·8 (398·9–427·3)	-189·2 (-201·0–-177·9)	-45·8 (-48·1–-43·5)	331·9 (320·3–339·0)	712·0 (696·9–727·5)	-380·1 (-393·6–-367·5)	-53·4 (-54·5–-52·3)
		Other musculoskeletal disorders	1608·4 (1102·7–2247·4)	1011·8 (679·5–1425·3)	596·6 (424·1–790·9)	59·5 (51·4–68·3)	1330·2 (907·6–1901·2)	714·7 (446·7–1075·9)	615·6 (447·8–815·3)	88·1 (69·1–113·5)
		Road injuries	280·7 (235·8–335·0)	703·7 (616·7–805·8)	-423·0 (-473·1–-378·5)	-60·2 (-62·0–-58·2)	631·9 (544·1–740·4)	1391·7 (1225·7–1594·0)	-759·8 (-856·5–-680·6)	-54·6 (-55·9–-53·3)
		Stroke	967·9 (885·3–1054·0)	1548·4 (1449·4–1651·4)	-580·6 (-613·9–-549·2)	-37·5 (-39·3–-35·5)	2293·8 (2178·2–2409·1)	3478·9 (3352·8–3608·6)	-1185·1 (-1252·0–-1120·0)	-34·1 (-35·8–-32·5)
		Tracheal, bronchus, and lung cancer	1456·8 (1383·4–1505·7)	2600·1 (2517·7–2668·8)	-1143·3 (-1203·2–-1085·3)	-44·0 (-45·9–-42·3)	1690·0 (1637·3–1722·6)	5640·8 (5547·8–5741·0)	-3950·8 (-4034·7–-3872·0)	-70·0 (-70·7–-69·4)
		Tuberculosis	11·8 (10·9–12·7)	33·3 (31·1–35·9)	-21·5 (-23·6–-19·7)	-64·5 (-67·1–-62·2)	73·4 (69·1–77·7)	255·0 (238·7–282·4)	-181·6 (-210·2–-165·2)	-71·2 (-73·9–-68·7)
	70+ years old	Age-related and other hearing loss	2382·0 (1713·0–3244·1)	2562·5 (1830·1–3536·4)	-180·5 (-318·7–-69·0)	-7·0 (-10·3–-3·1)	2319·3 (1646·8–3188·9)	2586·8 (1826·5–3584·1)	-267·5 (-431·1–-138·7)	-10·3 (-14·1–-6·2)
		Alzheimer's disease and other dementias	8974·9 (4281·9–17900·0)	5329·0 (2455·9–11554·2)	3645·9 (1785·4–6424·8)	70·8 (54·0–79·8)	7535·8 (3564·5–15968·3)	4615·6 (2148·9–10301·9)	2920·2 (1404·8–5732·7)	64·5 (53·4–72·3)
		Anxiety disorders	652·3 (448·6–933·6)	343·4 (230·1–490·6)	308·9 (198·4–453·6)	90·4 (69·2–111·0)	636·8 (443·3–903·8)	337·6 (226·2–475·8)	299·2 (188·0–439·2)	89·1 (64·4–112·1)
		COVID-19	4981·0 (4872·5–5103·5)	7776·9 (7638·8–7918·5)	-2795·9 (-2882·5–-2698·8)	-36·0 (-36·8–-34·9)	NA	NA	NA	NA
		Chronic kidney disease	2756·9 (2302·7–3075·9)	3298·8 (2969·0–3583·1)	-541·8 (-739·9–-361·5)	-16·5 (-24·0–-11·0)	1811·9 (1599·3–2004·1)	2396·7 (2193·1–2612·3)	-584·8 (-657·8–-517·2)	-24·4 (-28·3–-21·8)
		Chronic obstructive pulmonary disease	3832·1 (3290·6–4170·6)	5378·1 (4901·0–5697·3)	-1546·0 (-1758·3–-1350·4)	-28·8 (-33·6–-24·8)	3403·9 (3075·3–3664·1)	8167·6 (7761·5–8501·2)	-4763·7 (-4995·0–-4530·1)	-58·3 (-60·8–-56·0)

		Cirrhosis and other chronic liver diseases	548·3 (455·4–598·8)	949·1 (877·0–993·7)	-400·8 (-430·1–-368·9)	-42·3 (-48·2–-38·5)	808·1 (731·9–847·7)	1593·6 (1521·2–1639·6)	-785·5 (-820·2–-748·5)	-49·3 (-52·3–-47·4)
		Depressive disorders	795·1 (546·2–1085·4)	534·3 (367·5–729·7)	260·7 (181·7–358·8)	48·9 (42·3–54·9)	822·3 (569·2–1117·7)	559·9 (382·9–760·6)	262·4 (178·2–359·9)	47·0 (41·5–52·8)
		Diabetes mellitus	3113·5 (2522·1–3861·2)	4094·6 (3333·6–5037·7)	-981·0 (-1210·4–-784·8)	-24·0 (-26·8–-21·7)	3095·8 (2759·1–3494·2)	3322·7 (2966·3–3775·1)	-226·8 (-364·1–-109·7)	-6·8 (-11·2–-3·5)
		Falls	3394·9 (2675·4–4303·5)	2863·7 (2401·8–3480·6)	531·2 (276·1–842·0)	18·3 (11·3–24·7)	3061·6 (2436·4–3852·9)	2357·0 (1928·8–2872·5)	704·6 (470·5–975·7)	29·7 (23·5–35·7)
		HIV/AIDS	7·6 (4·9–11·3)	37·0 (26·6–50·2)	-29·4 (-39·6–-21·1)	-79·5 (-84·4–-74·5)	3·7 (3·3–4·3)	19·8 (17·2–23·3)	-16·2 (-19·0–-13·9)	-81·5 (-82·7–-80·1)
		Headache disorders	404·9 (140·6–805·3)	253·3 (100·4–494·7)	151·6 (36·1–334·9)	59·3 (28·7–90·6)	424·2 (147·7–836·9)	255·5 (99·9–494·6)	168·7 (44·5–358·7)	65·8 (33·6–98·8)
		Ischemic heart disease	6808·0 (5451·8–7550·2)	10667·2 (9650·8–11211·4)	-3859·1 (-4232·3–-3587·1)	-36·3 (-43·6–-32·4)	19384·4 (16983·4–20569·2)	29288·3 (27840·3–30047·2)	-9904·0 (-10795·8–-9356·2)	-33·9 (-38·7–-31·4)
		Low back pain	2937·8 (2097·1–3889·1)	2079·1 (1487·8–2762·0)	858·6 (608·1–1136·3)	41·4 (36·4–46·3)	2967·1 (2073·2–3953·8)	2107·4 (1460·5–2821·3)	859·7 (607·7–1149·6)	41·0 (35·3–46·7)
		Lower respiratory infections	1708·7 (1343·4–1908·2)	2547·0 (2267·5–2696·0)	-838·2 (-933·1–-755·2)	-33·0 (-40·7–-28·3)	3030·0 (2621·4–3227·9)	5028·0 (4716·1–5194·3)	-1998·0 (-2100·2–-1905·8)	-39·8 (-44·5–-37·4)
		Other musculoskeletal disorders	1211·0 (850·2–1694·6)	935·0 (635·6–1353·6)	276·0 (200·9–362·8)	30·1 (22·0–39·6)	948·3 (641·2–1413·1)	668·1 (408·8–1084·1)	280·3 (203·7–375·8)	43·5 (29·7–61·7)
		Road injuries	319·4 (265·7–383·4)	620·7 (528·4–728·0)	-301·4 (-348·8–-261·3)	-48·6 (-50·6–-46·8)	727·1 (617·3–860·9)	1348·8 (1170·7–1560·8)	-621·6 (-709·9–-547·7)	-46·1 (-47·7–-44·5)
		Stroke	5972·6 (4858·4–6628·0)	6397·4 (5831·6–6853·2)	-424·8 (-980·0–-93·2)	-6·8 (-16·7–-1·4)	13302·2 (11883·6–14138·7)	14846·0 (14048·8–15413·2)	-1543·8 (-2330·0–-1092·1)	-10·4 (-16·2–-7·3)
		Tracheal, bronchus, and lung cancer	2439·4 (2065·9–2639·0)	5405·4 (4959·8–5644·3)	-2966·0 (-3084·1–-2842·7)	-54·9 (-58·8–-52·5)	2111·8 (1911·7–2207·8)	8648·8 (8293·2–8841·8)	-6537·0 (-6674·4–-6359·2)	-75·6 (-77·0–-74·8)
		Tuberculosis	57·5 (45·2–65·2)	105·5 (94·2–115·4)	-48·0 (-55·5–-41·5)	-45·5 (-53·2–-40·0)	176·1 (157·1–191·8)	490·8 (457·3–542·6)	-314·7 (-369·2–-285·5)	-64·1 (-67·9–-61·0)
Latin America and Caribbean	10-24 years old	Age-related and other hearing loss	102·1 (65·5–148·6)	120·8 (77·7–176·3)	-18·8 (-29·0–-11·3)	-15·5 (-19·0–-11·8)	99·7 (63·4–145·8)	117·1 (74·1–174·1)	-17·4 (-27·2–-9·9)	-14·8 (-18·5–-11·3)
		Alzheimer's disease and other dementias	0·0 (0·0–0·0)	0·0 (0·0–0·0)	0·0 (0·0–0·0)	NA (NA–NA)	0·0 (0·0–0·0)	0·0 (0·0–0·0)	0·0 (0·0–0·0)	NA (NA–NA)
		Anxiety disorders	1157·4 (725·2–1669·8)	661·3 (410·5–983·1)	496·1 (313·3–712·5)	75·4 (63·6–86·3)	737·9 (464·1–1064·8)	453·6 (285·1–677·2)	284·2 (178·4–405·3)	62·9 (52·3–73·5)

		COVID-19	592·8 (425·8–923·5)	524·8 (366·3–767·9)	68·0 (-72·7–205·4)	13·8 (-12·6–41·3)	NA	NA	NA	NA
		Chronic kidney disease	117·4 (104·1–133·9)	133·2 (118·3–149·0)	-15·8 (-26·5–-2·2)	-11·8 (-19·2–-1·8)	125·4 (118·1–135·0)	125·6 (116·2–137·6)	-0·2 (-7·2–5·6)	-0·1 (-5·5–4·5)
		Chronic obstructive pulmonary disease	18·6 (16·0–21·7)	22·7 (20·1–25·4)	-4·1 (-6·3–-2·0)	-18·2 (-26·7–-8·9)	23·7 (20·8–26·8)	24·7 (22·5–27·2)	-1·0 (-3·5–1·2)	-4·0 (-14·3–5·0)
		Cirrhosis and other chronic liver diseases	40·7 (35·9–45·9)	64·1 (58·2–69·9)	-23·4 (-28·7–-18·5)	-36·5 (-42·8–-29·7)	79·0 (74·1–83·8)	114·1 (108·1–119·4)	-35·1 (-41·5–-29·1)	-30·7 (-35·4–-26·5)
		Depressive disorders	771·7 (482·6–1148·6)	380·2 (241·6–558·3)	391·5 (245·6–591·8)	103·1 (86·8–119·4)	560·9 (358·8–820·6)	265·8 (166·9–390·3)	295·1 (188·0–442·7)	111·0 (94·6–126·0)
		Diabetes mellitus	106·5 (87·7–131·5)	78·2 (63·8–98·5)	28·3 (22·7–35·3)	36·3 (30·8–43·4)	100·6 (88·2–116·7)	66·7 (57·5–79·1)	33·9 (29·6–38·9)	51·0 (45·3–57·5)
		Falls	134·1 (94·3–186·9)	270·2 (216·7–336·0)	-136·1 (-159·0–-117·7)	-50·7 (-57·3–-43·8)	204·2 (146·6–276·2)	492·1 (410·4–596·3)	-287·9 (-328·6–-257·6)	-58·7 (-64·4–-53·0)
		HIV/AIDS	86·8 (75·4–103·9)	136·9 (130·8–143·8)	-50·2 (-60·2–-36·0)	-36·7 (-43·8–-26·0)	117·5 (92·2–162·6)	151·5 (144·1–163·6)	-34·1 (-56·5–5·2)	-22·6 (-37·8–3·4)
		Headache disorders	1039·0 (84·7–2460·9)	595·0 (52·2–1378·9)	443·9 (33·6–1077·7)	73·6 (46·3–89·4)	1032·4 (82·4–2414·0)	577·6 (49·6–1341·0)	454·8 (32·0–1082·5)	77·4 (49·3–95·2)
		Ischemic heart disease	50·2 (45·3–55·7)	115·5 (106·7–124·0)	-65·3 (-72·5–-58·3)	-56·5 (-59·9–-52·8)	58·3 (55·0–62·0)	93·2 (88·5–97·9)	-35·0 (-40·7–-28·2)	-37·5 (-42·0–-31·7)
		Low back pain	628·6 (406·3–896·5)	372·2 (240·7–529·5)	256·4 (165·8–370·5)	69·0 (60·9–77·8)	600·9 (388·9–856·5)	338·0 (217·2–477·3)	262·9 (170·0–384·6)	77·9 (67·7–88·8)
		Lower respiratory infections	128·2 (115·9–142·7)	163·5 (149·4–178·9)	-35·2 (-46·2–-25·6)	-21·5 (-27·6–-15·8)	256·8 (243·9–270·6)	300·1 (287·1–313·5)	-43·3 (-57·1–-29·4)	-14·4 (-18·8–-10·1)
		Other musculoskeletal disorders	224·9 (165·7–302·1)	130·7 (84·6–191·9)	94·2 (78·0–113·2)	74·3 (53·9–99·6)	192·4 (141·7–260·5)	101·1 (64·4–153·2)	91·3 (75·3–110·1)	93·4 (69·0–125·2)
		Road injuries	371·5 (339·2–405·5)	1439·3 (1343·1–1538·5)	-1067·8 (-1145·5–-988·7)	-74·2 (-75·6–-72·9)	629·1 (604·2–655·9)	2051·3 (1998·7–2107·9)	-1422·1 (-1469·3–-1377·3)	-69·3 (-70·3–-68·3)
		Stroke	102·6 (91·3–113·8)	102·4 (93·0–112·3)	0·2 (-7·4–8·7)	0·2 (-7·0–8·4)	168·1 (158·0–179·3)	158·0 (149·8–166·3)	10·2 (0·6–20·9)	6·5 (0·4–13·3)
		Tracheal, bronchus, and lung cancer	8·0 (7·2–9·0)	10·2 (9·4–11·1)	-2·2 (-2·9–-1·5)	-21·7 (-27·5–-15·4)	7·9 (7·4–8·5)	14·3 (13·6–15·1)	-6·3 (-7·1–-5·7)	-44·4 (-47·9–-40·7)
		Tuberculosis	52·0 (44·9–61·0)	67·8 (57·6–90·3)	-15·8 (-39·8–-7·7)	-22·7 (-43·5–-12·4)	235·4 (213·3–256·6)	238·0 (208·5–300·0)	-2·6 (-57·8–29·8)	-0·5 (-19·2–13·7)
	25-49 years old	Age-related and other hearing loss	263·7 (167·1–393·6)	294·7 (184·6–443·2)	-31·0 (-53·5–-16·1)	-10·4 (-13·2–-7·7)	250·8 (158·3–372·0)	282·5 (175·3–419·5)	-31·7 (-54·4–-16·9)	-11·1 (-13·6–-8·1)
		Alzheimer's disease and	7·3 (3·7–14·8)	6·3 (3·2–13·1)	0·9 (-0·5–2·8)	16·3 (-9·0–51·4)	5·5 (2·8–11·1)	5·1 (2·5–10·2)	0·5 (-0·6–1·8)	10·7 (-12·3–43·0)

		other dementias								
		Anxiety disorders	1415·6 (935·7–1979·4)	754·6 (501·7–1078·6)	661·1 (422·3–946·1)	88·0 (72·7–104·4)	922·6 (623·9–1290·7)	538·6 (351·3–770·6)	384·0 (248·4–553·2)	71·6 (58·0–84·4)
		COVID-19	2964·5 (2557·5–3536·9)	5223·2 (4703·8–5856·9)	-2258·6 (-2845·6–-1658·8)	-43·2 (-51·2–-33·9)	NA	NA	NA	NA
		Chronic kidney disease	436·7 (376·3–515·2)	552·6 (499·3–613·3)	-115·9 (-172·5–-44·4)	-20·9 (-30·2–-8·3)	369·2 (342·7–400·8)	409·7 (381·0–445·3)	-40·5 (-55·9–-26·7)	-9·9 (-13·5–-6·7)
		Chronic obstructive pulmonary disease	90·6 (81·3–101·5)	120·4 (111·0–130·1)	-29·8 (-38·4–-20·8)	-24·7 (-31·6–-17·6)	115·9 (106·6–125·9)	146·4 (138·3–154·6)	-30·5 (-39·0–-22·6)	-20·8 (-26·2–-15·4)
		Cirrhosis and other chronic liver diseases	193·7 (171·4–215·0)	1015·6 (932·6–1103·6)	-821·9 (-901·6–-743·4)	-80·9 (-83·0–-78·8)	362·8 (351·7–374·2)	1541·0 (1515·2–1570·0)	-1178·2 (-1207·7–-1149·9)	-76·5 (-77·3–-75·6)
		Depressive disorders	1356·0 (892·6–1892·3)	693·4 (461·6–978·1)	662·7 (418·8–954·8)	95·6 (79·2–110·5)	1084·8 (728·9–1535·7)	570·0 (377·4–796·0)	514·8 (331·4–741·5)	90·4 (75·8–103·8)
		Diabetes mellitus	773·6 (620·0–960·3)	841·6 (706·4–1013·3)	-68·1 (-113·2–-17·5)	-8·2 (-13·9–-2·2)	572·5 (484·6–676·8)	617·5 (532·9–717·2)	-45·0 (-60·9–-29·9)	-7·3 (-10·3–-4·6)
		Falls	235·7 (166·0–322·0)	657·0 (527·0–813·0)	-421·3 (-503·7–-358·7)	-64·3 (-68·6–-59·8)	318·1 (229·7–427·9)	1053·5 (860·5–1281·1)	-735·5 (-877·0–-627·9)	-70·0 (-73·8–-66·2)
		HIV/AIDS	396·6 (368·8–436·4)	858·5 (826·0–902·7)	-461·9 (-508·8–-417·8)	-53·8 (-57·3–-49·4)	353·5 (279·5–468·7)	920·6 (849·1–1046·6)	-567·1 (-646·1–-509·3)	-61·7 (-67·7–-53·0)
		Headache disorders	1095·4 (195·6–2283·1)	584·3 (122·9–1226·7)	511·1 (68·8–1114·5)	85·3 (47·4–107·7)	1092·9 (184·1–2304·8)	577·7 (118·1–1237·4)	515·2 (65·3–1133·2)	86·9 (46·2–112·2)
		Ischemic heart disease	438·2 (400·1–480·7)	1116·4 (1034·5–1202·0)	-678·2 (-738·7–-614·4)	-60·7 (-63·3–-57·8)	616·5 (599·3–634·7)	1387·3 (1349·9–1419·9)	-770·8 (-804·0–-726·9)	-55·6 (-57·1–-53·6)
		Low back pain	1366·3 (938·8–1891·4)	776·7 (528·6–1065·2)	589·7 (408·7–822·6)	76·1 (69·6–83·2)	1332·6 (901·3–1844·2)	741·5 (502·5–1018·3)	591·1 (404·4–835·9)	79·8 (72·5–88·7)
		Lower respiratory infections	221·6 (199·9–243·5)	409·5 (380·5–441·7)	-187·9 (-216·9–-161·3)	-45·9 (-50·6–-41·4)	320·9 (306·9–336·2)	571·3 (552·1–592·6)	-250·4 (-270·7–-228·1)	-43·8 (-46·3–-40·8)
		Other musculoskeletal disorders	1156·8 (810·5–1666·0)	821·2 (545·7–1212·8)	335·6 (257·7–430·8)	41·6 (33·7–51·3)	1102·6 (774·5–1572·2)	762·4 (503·7–1141·2)	340·3 (259·6–433·2)	45·5 (36·2–56·6)
		Road injuries	408·6 (373·6–448·8)	2126·1 (1998·0–2273·8)	-1717·5 (-1835·9–-1612·2)	-80·8 (-81·9–-79·7)	679·7 (647·5–720·4)	3226·1 (3127·3–3343·7)	-2546·4 (-2628·2–-2469·3)	-78·9 (-79·5–-78·3)
		Stroke	477·3 (439·0–520·1)	528·2 (489·5–567·9)	-51·0 (-79·3–-21·4)	-9·6 (-14·8–-4·1)	954·2 (919·7–988·9)	1022·4 (988·3–1055·8)	-68·2 (-104·0–-26·0)	-6·7 (-10·0–-2·6)
		Tracheal, bronchus, and lung cancer	97·2 (88·2–107·4)	112·7 (104·3–122·6)	-15·5 (-23·4–-7·7)	-13·7 (-19·9–-6·9)	100·2 (96·8–103·7)	184·9 (178·5–192·4)	-84·6 (-90·9–-78·4)	-45·8 (-47·8–-43·5)

		Tuberculosis	86·0 (74·8–98·6)	202·5 (178·3–258·6)	-116·6 (-172·1–-98·8)	-57·3 (-66·8–-52·4)	427·2 (395·6–456·2)	730·5 (669·4–886·4)	-303·4 (-449·9–-238·4)	-41·3 (-51·4–-35·1)
	50-69 years old	Age-related and other hearing loss	961·5 (625·4–1433·8)	1045·5 (666·1–1558·2)	-84·0 (-145·7–-43·4)	-8·0 (-10·0–-5·7)	961·4 (622·2–1423·6)	1044·2 (668·3–1554·6)	-82·7 (-134·6–-43·3)	-7·9 (-9·8–-5·6)
		Alzheimer's disease and other dementias	513·8 (237·0–1187·3)	421·4 (190·7–997·4)	92·4 (39·8–192·9)	22·6 (15·2–31·1)	501·6 (231·2–1149·2)	411·5 (183·7–979·6)	90·2 (40·4–187·0)	22·7 (16·4–31·1)
		Anxiety disorders	1108·3 (733·9–1555·6)	661·0 (440·8–939·1)	447·4 (270·8–658·3)	68·3 (48·7–88·6)	845·7 (570·7–1193·4)	545·0 (362·8–779·8)	300·7 (182·6–446·1)	55·7 (37·5–75·2)
		COVID-19	8967·7 (8067·1–9994·6)	17117·2 (15575·0–18817·8)	-8149·5 (-9836·7–-6447·4)	-47·5 (-53·8–-40·8)	NA	NA	NA	NA
		Chronic kidney disease	1970·7 (1716·0–2304·3)	2484·4 (2222·9–2768·5)	-513·8 (-790·2–-180·2)	-20·6 (-30·6–-7·6)	1304·8 (1232·4–1392·0)	1639·0 (1535·6–1767·3)	-334·2 (-406·7–-265·2)	-20·4 (-23·9–-16·7)
		Chronic obstructive pulmonary disease	882·9 (824·6–951·7)	1193·8 (1109·5–1277·7)	-310·8 (-378·5–-238·5)	-26·0 (-30·3–-20·8)	1133·6 (1066·2–1200·9)	1822·2 (1757·6–1888·9)	-688·6 (-761·9–-619·5)	-37·8 (-41·4–-34·5)
		Cirrhosis and other chronic liver diseases	812·0 (721·9–904·9)	2749·2 (2445·3–3036·1)	-1937·2 (-2189·7–-1680·4)	-70·4 (-73·9–-67·1)	1190·7 (1141·8–1245·6)	3595·2 (3496·5–3689·7)	-2404·5 (-2493·4–-2314·4)	-66·9 (-68·1–-65·5)
		Depressive disorders	1349·4 (889·7–1859·6)	823·0 (539·3–1136·6)	526·4 (337·2–764·7)	64·2 (50·9–78·1)	1273·1 (862·6–1759·8)	727·4 (486·0–1005·5)	545·8 (348·7–773·0)	75·3 (62·4–90·5)
		Diabetes mellitus	4213·8 (3583·4–5045·4)	4573·6 (3946·7–5381·4)	-359·8 (-659·7–-79·4)	-7·9 (-14·1–-1·7)	4236·0 (3803·6–4779·8)	3696·3 (3298·2–4213·4)	539·6 (444·0–638·6)	14·6 (11·9–17·5)
		Falls	507·6 (388·8–650·3)	1198·2 (990·8–1444·6)	-690·6 (-808·2–-593·2)	-57·8 (-61·2–-54·5)	633·4 (490·0–792·2)	1617·6 (1344·8–1963·8)	-984·1 (-1169·6–-849·8)	-60·9 (-63·8–-58·2)
		HIV/AIDS	224·2 (201·9–249·1)	523·5 (493·6–561·8)	-299·3 (-335·9–-266·2)	-57·2 (-61·3–-52·2)	174·8 (131·9–238·7)	331·7 (288·6–404·2)	-156·9 (-202·9–-123·3)	-47·4 (-56·4–-36·2)
		Headache disorders	827·3 (229·2–1714·7)	445·0 (139·9–892·8)	382·2 (79·9–787·5)	83·7 (53·1–107·1)	828·4 (226·9–1687·6)	442·5 (142·0–882·4)	385·9 (80·5–812·1)	85·1 (50·2–107·7)
		Ischemic heart disease	2869·6 (2656·7–3096·9)	6298·9 (5794·4–6854·4)	-3429·3 (-3857·9–-3040·2)	-54·4 (-57·1–-51·6)	4833·3 (4690·4–4952·4)	9239·2 (9016·0–9416·8)	-4405·8 (-4584·7–-4156·5)	-47·7 (-49·0–-45·9)
		Low back pain	2089·6 (1431·0–2910·4)	1296·2 (884·6–1814·0)	793·5 (545·7–1103·1)	61·4 (55·1–68·1)	2075·5 (1416·9–2909·7)	1250·8 (853·9–1757·0)	824·7 (553·6–1167·3)	66·1 (59·6–73·3)
		Lower respiratory infections	781·2 (716·0–848·6)	1272·3 (1175·4–1394·5)	-491·1 (-586·3–-402·4)	-38·5 (-43·4–-33·4)	965·6 (916·3–1016·6)	1575·6 (1517·0–1639·7)	-609·9 (-674·7–-542·3)	-38·7 (-41·9–-35·4)

		Other musculoskeletal disorders	1664·4 (1145·1–2308·1)	1195·3 (785·0–1717·2)	469·1 (340·9–605·2)	39·8 (32·6–48·2)	1580·8 (1078·5–2215·0)	1087·5 (708·8–1581·5)	493·3 (360·0–645·6)	46·1 (37·0–56·7)
		Road injuries	383·0 (346·4–429·5)	1565·0 (1434·9–1724·9)	-1182·0 (-1313·9–-1076·5)	-75·5 (-76·7–-74·3)	749·3 (695·8–815·0)	2501·2 (2346·9–2661·9)	-1752·0 (-1856·9–-1646·3)	-70·0 (-70·8–-69·3)
		Stroke	2062·1 (1912·8–2229·2)	2848·0 (2658·4–3058·8)	-785·9 (-945·9–-640·9)	-27·6 (-31·5–-23·5)	4602·1 (4439·8–4747·7)	5979·1 (5807·7–6143·8)	-1377·1 (-1555·5–-1214·9)	-23·0 (-25·6–-20·6)
		Tracheal, bronchus, and lung cancer	810·7 (746·2–880·3)	1267·1 (1173·7–1370·5)	-456·4 (-544·3–-384·3)	-36·0 (-40·5–-31·8)	742·3 (716·3–765·2)	2026·4 (1967·4–2084·3)	-1284·0 (-1334·2–-1237·5)	-63·4 (-64·4–-62·4)
		Tuberculosis	109·5 (97·3–122·9)	317·0 (274·1–396·3)	-207·4 (-281·1–-173·8)	-65·3 (-72·0–-61·4)	585·2 (541·1–620·5)	1286·6 (1164·7–1636·1)	-701·3 (-1058·6–-581·9)	-54·2 (-64·4–-49·4)
	70+ years old	Age-related and other hearing loss	2787·6 (1999·2–3848·8)	2841·0 (2034·2–3909·0)	-53·4 (-136·9–22·8)	-1·9 (-4·4–0·8)	2723·9 (1952·6–3784·7)	2816·0 (1993·9–3915·6)	-92·1 (-162·0–-17·9)	-3·3 (-5·4–-0·7)
		Alzheimer's disease and other dementias	6053·3 (2942·2–12428·6)	4337·5 (2045·0–9310·7)	1715·9 (835·7–3210·2)	40·4 (32·9–46·6)	5199·7 (2522·0–11167·9)	3875·5 (1834·8–8597·9)	1324·2 (660·4–2621·6)	34·9 (29·3–40·4)
		Anxiety disorders	735·6 (501·1–1028·1)	431·2 (291·1–609·3)	304·3 (186·2–455·1)	70·9 (50·4–90·3)	649·2 (445·3–917·6)	398·4 (266·1–559·7)	250·8 (149·9–379·8)	63·2 (42·3–80·9)
		COVID-19	17762·1 (16142·2–19470·9)	31889·8 (29413·6–34772·2)	-14127·7 (-16724·1–-11370·5)	-44·2 (-49·6–-38·0)	NA	NA	NA	NA
		Chronic kidney disease	4067·8 (3630·4–4460·6)	5251·0 (4763·4–5744·9)	-1183·2 (-1522·3–-808·4)	-22·5 (-28·0–-16·3)	3082·0 (2848·8–3312·9)	3847·2 (3584·5–4149·4)	-765·2 (-939·9–-610·2)	-19·9 (-23·5–-16·2)
		Chronic obstructive pulmonary disease	4161·2 (3663·9–4528·2)	5855·1 (5362·4–6256·8)	-1693·9 (-1956·7–-1415·4)	-28·9 (-33·6–-24·6)	4932·6 (4555·4–5233·0)	7882·1 (7465·4–8189·2)	-2949·5 (-3182·5–-2717·4)	-37·4 (-40·3–-34·6)
		Cirrhosis and other chronic liver diseases	1056·6 (938·1–1171·8)	2063·7 (1835·8–2271·3)	-1007·1 (-1183·2–-831·8)	-48·7 (-54·0–-44·1)	1360·8 (1274·9–1435·8)	2650·2 (2521·9–2750·1)	-1289·3 (-1378·1–-1201·4)	-48·6 (-50·8–-45·8)
		Depressive disorders	1047·2 (717·7–1429·9)	780·9 (532·6–1077·9)	266·3 (171·5–386·0)	34·3 (25·9–43·4)	1080·6 (743·2–1512·7)	791·5 (538·8–1110·7)	289·1 (190·3–418·0)	36·7 (27·5–46·5)
		Diabetes mellitus	7734·0 (6855·1–8955·1)	7599·9 (6712·0–8681·9)	134·1 (-308·9–548·8)	1·8 (-4·0–7·5)	8056·0 (7383·2–8843·0)	6152·5 (5587·1–6806·3)	1903·5 (1679·1–2114·6)	31·0 (27·2–34·4)
		Falls	2062·2 (1738·3–2435·8)	2113·0 (1810·7–2479·0)	-50·8 (-142·8–41·4)	-2·4 (-7·0–1·9)	2121·9 (1791·3–2498·4)	2377·2 (2042·4–2794·7)	-255·3 (-370·1–-160·7)	-10·8 (-14·9–-6·6)
		HIV/AIDS	29·4 (24·9–35·3)	82·4 (73·9–92·9)	-53·0 (-61·5–-45·7)	-64·3 (-69·3–-58·2)	24·0 (14·9–38·3)	44·4 (32·2–64·4)	-20·4 (-32·8–-13·1)	-46·2 (-60·0–-29·8)

		Headache disorders	413·9 (140·0–839·4)	246·7 (85·3–498·8)	167·1 (51·6–354·7)	67·7 (40·1–83·4)	420·9 (144·6–859·8)	248·7 (88·0–499·3)	172·2 (50·8–367·7)	69·3 (43·3–85·1)
		Ischemic heart disease	10636·4 (9331·3–11589·8)	15076·6 (13765·6–16367·4)	-4440·3 (-5233·7–-3632·2)	-29·4 (-34·3–-25·1)	17521·6 (16203·1–18157·0)	21488·6 (20437·5–22081·4)	-3967·0 (-4443·7–-3481·0)	-18·5 (-21·2–-16·2)
		Low back pain	2419·4 (1678·4–3257·9)	1576·1 (1083·5–2129·2)	843·3 (591·4–1136·5)	53·7 (46·7–60·8)	2375·1 (1641·4–3233·6)	1497·1 (1017·6–2028·9)	878·0 (616·2–1195·2)	58·8 (51·4–65·7)
		Lower respiratory infections	3705·9 (3127·4–4098·1)	4590·2 (4082·5–4982·2)	-884·3 (-1159·6–-628·1)	-19·3 (-25·5–-13·9)	4886·5 (4446·5–5170·3)	5799·0 (5460·1–6072·8)	-912·5 (-1132·9–-649·5)	-15·7 (-19·8–-11·3)
		Other musculoskeletal disorders	1491·2 (1018·4–2106·9)	1157·6 (780·3–1690·5)	333·6 (237·8–454·9)	29·3 (22·2–37·2)	1354·5 (925·6–1948·0)	978·1 (635·7–1473·7)	376·4 (266·2–515·7)	39·3 (30·0–51·8)
		Road injuries	404·3 (360·8–458·7)	1164·1 (1043·6–1310·1)	-759·8 (-855·0–-679·0)	-65·3 (-66·7–-63·8)	792·8 (727·6–867·8)	1983·3 (1836·2–2157·1)	-1190·5 (-1295·5–-1096·2)	-60·0 (-60·9–-59·1)
		Stroke	6925·0 (6084·4–7515·8)	8202·2 (7520·1–8798·4)	-1277·2 (-1653·6–-942·5)	-15·6 (-20·8–-11·6)	14442·4 (13301·9–15048·2)	15450·5 (14652·4–15936·8)	-1008·1 (-1505·1–-561·9)	-6·5 (-10·0–-3·6)
		Tracheal, bronchus, and lung cancer	1254·9 (1107·4–1371·8)	2408·0 (2218·3–2595·0)	-1153·1 (-1290·0–-1038·2)	-47·9 (-51·9–-44·5)	1191·3 (1114·5–1242·0)	3165·9 (3012·8–3267·4)	-1974·6 (-2051·0–-1895·1)	-62·4 (-63·6–-61·1)
		Tuberculosis	152·3 (133·4–172·0)	381·0 (331·3–460·2)	-228·7 (-301·1–-189·3)	-59·9 (-66·6–-55·7)	767·6 (702·7–822·3)	1693·8 (1533·7–2115·4)	-926·2 (-1343·7–-759·1)	-54·4 (-63·8–-49·1)
North Africa and Middle East	10-24 years old	Age-related and other hearing loss	126·8 (80·6–186·0)	144·7 (92·0–211·6)	-17·9 (-30·4–-7·5)	-12·3 (-17·4–-6·6)	129·5 (82·8–192·0)	147·3 (93·6–217·8)	-17·8 (-31·0–-6·5)	-12·0 (-17·4–-6·2)
		Alzheimer's disease and other dementias	0·0 (0·0–0·0)	0·0 (0·0–0·0)	0·0 (0·0–0·0)	NA (NA–NA)	0·0 (0·0–0·0)	0·0 (0·0–0·0)	0·0 (0·0–0·0)	NA (NA–NA)
		Anxiety disorders	1233·3 (758·3–1824·6)	781·8 (478·8–1177·4)	451·6 (266·8–700·4)	58·3 (41·9–74·2)	983·1 (618·9–1412·3)	639·0 (399·0–951·2)	344·1 (212·2–518·4)	54·3 (40·1–68·8)
		COVID-19	447·8 (309·8–708·4)	447·7 (339·5–656·5)	0·1 (-42·2–67·8)	-0·8 (-11·2–11·6)	NA	NA	NA	NA
		Chronic kidney disease	126·2 (103·4–155·9)	102·8 (84·8–127·4)	23·5 (-8·9–48·2)	23·8 (-7·3–52·5)	136·6 (111·7–178·8)	108·3 (84·3–152·2)	28·3 (-14·2–63·7)	28·1 (-9·3–77·4)
		Chronic obstructive pulmonary disease	38·4 (32·5–45·1)	35·6 (31·1–41·3)	2·9 (-3·3–9·0)	8·4 (-8·8–28·4)	44·7 (31·4–54·4)	48·3 (38·7–56·9)	-3·6 (-18·5–6·1)	-7·1 (-35·7–14·1)
		Cirrhosis and other chronic liver diseases	83·2 (66·6–103·1)	70·2 (57·1–89·7)	13·0 (-8·4–29·7)	19·5 (-9·8–45·3)	154·1 (120·1–193·5)	135·1 (107·5–170·3)	19·0 (-17·3–68·2)	15·3 (-12·1–60·8)

		Depressive disorders	1077·0 (634·8–1648·0)	672·7 (404·2–1029·3)	404·3 (237·0–621·5)	60·2 (50·8–70·6)	865·5 (541·1–1283·4)	520·1 (318·1–783·0)	345·4 (215·5–512·9)	66·6 (59·2–74·4)
		Diabetes mellitus	99·2 (77·4–127·3)	74·0 (54·9–100·9)	25·2 (17·0–35·5)	34·7 (21·2–51·4)	74·5 (61·1–100·1)	46·1 (37·2–57·9)	28·4 (17·0–48·5)	62·3 (36·6–103·3)
		Falls	128·3 (99·7–168·6)	235·2 (191·8–285·7)	-106·9 (-146·2–-81·2)	-45·5 (-55·5–-35·0)	164·9 (124·5–216·4)	311·5 (260·3–388·8)	-146·6 (-204·1–-117·1)	-47·0 (-56·5–-38·5)
		HIV/AIDS	36·1 (20·4–79·4)	17·8 (12·0–28·4)	18·3 (6·3–52·3)	100·1 (35·1–233·5)	5·1 (3·2–11·9)	2·7 (2·0–4·3)	2·4 (0·9–7·5)	85·5 (34·0–213·9)
		Headache disorders	917·1 (133·5–2122·8)	640·0 (86·0–1496·6)	277·1 (43·1–641·9)	44·5 (28·0–64·5)	889·6 (128·1–2069·4)	625·8 (83·2–1435·6)	263·9 (39·4–617·8)	43·3 (27·6–60·8)
		Ischemic heart disease	154·1 (121·2–190·4)	233·7 (191·8–279·4)	-79·6 (-121·3–-40·5)	-33·8 (-47·0–-19·0)	259·7 (208·9–314·4)	284·4 (245·7–324·6)	-24·7 (-98·4–48·0)	-8·1 (-31·8–17·2)
		Low back pain	765·5 (502·0–1074·1)	430·2 (281·0–610·8)	335·4 (219·0–479·2)	78·3 (65·4–91·3)	764·8 (504·4–1073·9)	430·2 (283·2–602·9)	334·6 (221·4–476·1)	78·0 (65·6–90·4)
		Lower respiratory infections	156·8 (129·2–201·6)	143·3 (120·3–169·9)	13·5 (-13·6–55·8)	9·7 (-8·3–37·9)	331·8 (283·0–442·0)	299·9 (264·7–337·0)	31·9 (-19·7–141·1)	10·9 (-6·2–47·9)
		Other musculoskeletal disorders	134·3 (92·0–191·0)	60·5 (34·9–96·5)	73·8 (53·0–97·8)	126·7 (87·9–176·3)	92·2 (63·3–131·4)	32·3 (19·1–52·7)	60·0 (42·7–82·5)	193·5 (129·7–282·5)
		Road injuries	384·1 (335·1–438·4)	1452·1 (1295·5–1629·4)	-1068·0 (-1206·2–-937·6)	-73·5 (-76·0–-70·9)	841·9 (750·8–930·5)	2710·5 (2498·3–2925·8)	-1868·6 (-2060·5–-1687·7)	-68·9 (-72·2–-66·1)
		Stroke	158·4 (130·2–188·5)	181·0 (152·3–208·4)	-22·6 (-49·6–3·4)	-12·3 (-25·9–1·9)	316·1 (269·0–370·5)	328·2 (282·7–374·0)	-12·1 (-74·3–45·6)	-3·4 (-20·8–15·3)
		Tracheal, bronchus, and lung cancer	8·6 (6·8–10·6)	12·9 (10·7–15·8)	-4·3 (-7·1–-1·8)	-33·0 (-49·0–-15·3)	7·2 (5·4–10·2)	17·5 (13·6–22·9)	-10·3 (-16·1–-5·1)	-58·0 (-72·6–-35·6)
		Tuberculosis	94·8 (78·0–115·2)	41·9 (26·3–88·7)	52·9 (6·5–77·4)	142·2 (7·4–270·5)	265·5 (221·7–308·8)	127·3 (73·9–272·8)	138·1 (3·1–199·9)	125·6 (1·1–261·0)
	25-49 years old	Age-related and other hearing loss	263·5 (173·0–379·5)	298·4 (191·2–435·7)	-34·9 (-61·6–-17·1)	-11·5 (-15·0–-7·6)	264·6 (177·1–376·7)	300·1 (196·4–436·6)	-35·5 (-59·3–-17·7)	-11·7 (-15·4–-7·6)
		Alzheimer's disease and other dementias	7·2 (3·7–15·1)	6·1 (3·1–12·3)	1·1 (-0·4–3·2)	19·9 (-7·8–58·6)	6·2 (3·2–12·7)	5·1 (2·6–10·2)	1·1 (-0·3–3·0)	23·6 (-5·4–64·0)
		Anxiety disorders	1113·2 (704·4–1575·5)	626·8 (390·0–916·7)	486·3 (292·5–737·8)	78·3 (53·8–105·1)	929·6 (611·2–1334·2)	517·2 (338·4–752·1)	412·3 (256·5–608·4)	80·1 (60·9–102·5)
		COVID-19	1883·3 (1575·8–2320·5)	3408·3 (3005·5–3865·6)	-1525·1 (-1774·0–-1257·8)	-44·8 (-49·4–-36·7)	NA	NA	NA	NA
		Chronic kidney disease	374·5 (313·9–441·2)	354·8 (288·7–417·2)	19·7 (-37·2–84·0)	6·0 (-10·3–28·6)	332·5 (277·8–425·9)	317·1 (250·5–447·1)	15·4 (-98·8–96·6)	6·3 (-21·9–40·1)
		Chronic obstructive	155·1 (133·0–178·0)	172·0 (149·7–194·0)	-16·9 (-40·1–3·5)	-9·7 (-21·8–2·0)	188·2 (138·1–224·9)	232·4 (194·6–263·9)	-44·3 (-104·8–-2·0)	-18·8 (-41·5–-1·0)

		pulmonary disease								
		Cirrhosis and other chronic liver diseases	173·9 (136·7–217·7)	306·8 (261·6–371·9)	-133·0 (-184·4–-92·5)	-43·2 (-53·3–-31·5)	362·3 (315·1–409·7)	635·2 (530·5–735·6)	-272·9 (-384·5–-162·0)	-42·6 (-52·5–-29·1)
		Depressive disorders	1594·0 (1039·9–2297·2)	1043·1 (684·1–1477·6)	550·9 (335·8–819·8)	52·9 (41·3–65·0)	1403·5 (942·9–2014·7)	880·3 (579·3–1260·8)	523·1 (348·1–766·5)	59·5 (50·9–68·0)
		Diabetes mellitus	649·6 (491·0–855·6)	636·8 (474·4–832·2)	12·8 (-19·1–52·5)	2·1 (-2·8–9·3)	328·1 (269·5–399·0)	297·1 (239·3–361·8)	31·0 (3·6–59·3)	10·6 (1·0–20·8)
		Falls	230·1 (163·8–312·8)	542·5 (434·3–685·3)	-312·4 (-385·4–-257·7)	-57·8 (-62·8–-52·5)	245·7 (182·7–329·6)	552·7 (446·8–701·7)	-307·0 (-397·7–-254·4)	-55·7 (-61·6–-49·6)
		HIV/AIDS	182·8 (114·8–352·2)	152·2 (104·1–299·1)	30·6 (-31·9–110·7)	21·1 (-15·0–58·0)	21·4 (14·1–43·5)	26·6 (18·0–49·3)	-5·2 (-11·6–0·5)	-20·6 (-32·9–1·3)
		Headache disorders	1247·3 (295·3–2585·7)	709·3 (211·9–1445·4)	538·0 (81·9–1211·0)	72·7 (38·7–100·9)	1238·5 (291·8–2562·8)	710·2 (200·2–1446·9)	528·3 (85·5–1178·6)	71·5 (38·4–98·0)
		Ischemic heart disease	996·5 (791·2–1235·8)	2044·4 (1755·6–2395·1)	-1048·0 (-1265·6–-863·4)	-51·3 (-58·7–-43·6)	1590·0 (1346·6–1891·0)	2829·5 (2570·7–3111·2)	-1239·4 (-1597·8–-892·8)	-43·7 (-53·3–-32·9)
		Low back pain	1375·5 (944·8–1902·5)	970·0 (654·9–1329·6)	405·6 (280·1–565·8)	42·0 (36·2–48·4)	1344·6 (928·1–1851·5)	996·6 (683·7–1360·8)	348·1 (241·0–485·2)	35·0 (29·6–40·7)
		Lower respiratory infections	172·1 (134·9–213·7)	196·9 (170·1–227·2)	-24·8 (-57·7–0·8)	-12·5 (-28·2–0·4)	292·7 (247·3–373·7)	303·1 (272·7–340·3)	-10·3 (-59·0–62·4)	-3·2 (-18·8–20·5)
		Other musculoskeletal disorders	789·1 (540·8–1146·2)	484·5 (306·4–725·0)	304·6 (218·3–411·9)	64·0 (51·4–78·2)	590·7 (400·9–850·5)	328·6 (207·9–510·7)	262·1 (186·2–348·6)	81·4 (64·3–102·6)
		Road injuries	583·6 (485·8–675·9)	2119·4 (1882·3–2389·1)	-1535·8 (-1764·0–-1351·2)	-72·5 (-76·9–-70·0)	1031·9 (902·4–1162·0)	3341·4 (3093·6–3589·6)	-2309·5 (-2513·9–-2110·6)	-69·1 (-72·1–-66·0)
		Stroke	662·1 (548·3–794·5)	669·6 (576·2–787·7)	-7·5 (-104·7–98·2)	-1·0 (-15·1–14·8)	1155·8 (966·1–1343·5)	1118·9 (974·7–1249·3)	36·9 (-172·4–231·5)	3·5 (-15·0–22·0)
		Tracheal, bronchus, and lung cancer	79·9 (65·9–95·4)	174·2 (148·7–203·4)	-94·3 (-119·0–-71·3)	-54·0 (-61·3–-46·4)	72·5 (58·3–96·4)	271·6 (210·3–341·7)	-199·0 (-269·6–-136·1)	-72·9 (-80·1–-61·2)
		Tuberculosis	166·5 (130·5–216·0)	122·3 (86·1–193·0)	44·1 (-9·9–87·5)	39·7 (-6·2–85·8)	527·5 (445·3–617·8)	417·1 (254·2–789·9)	110·4 (-225·9–308·4)	34·2 (-28·8–118·4)
	50-69 years old	Age-related and other hearing loss	904·8 (589·9–1314·9)	1041·6 (670·2–1520·7)	-136·8 (-223·5–-77·5)	-13·1 (-15·5–-10·2)	957·5 (630·6–1402·9)	1120·9 (724·4–1627·2)	-163·4 (-258·2–-98·2)	-14·6 (-17·4–-11·5)
		Alzheimer's disease and other dementias	505·3 (237·4–1151·8)	408·0 (188·4–956·6)	97·4 (43·1–200·1)	24·8 (16·7–34·2)	555·5 (256·2–1243·7)	452·8 (205·9–1030·1)	102·7 (43·6–216·8)	23·1 (14·7–32·9)
		Anxiety disorders	814·2 (526·4–1196·5)	498·4 (310·3–742·3)	315·8 (178·3–477·3)	64·1 (39·9–87·8)	714·6 (476·2–1042·6)	448·9 (297·1–651·4)	265·7 (157·7–407·1)	59·8 (38·9–82·1)

		COVID-19	6176·5 (5461·2–7060·5)	12551·8 (10964·8–14041·4)	-6375·4 (-7139·5–-5461·2)	-50·8 (-52·6–-47·2)	NA	NA	NA	NA
		Chronic kidney disease	1876·7 (1611·4–2185·8)	1822·2 (1499·6–2134·2)	54·5 (-227·0–340·8)	3·4 (-12·1–20·4)	1626·7 (1348·7–2118·1)	1686·8 (1330·5–2699·9)	-60·1 (-1019·6–597·4)	-0·8 (-38·2–43·3)
		Chronic obstructive pulmonary disease	929·6 (811·0–1071·0)	1434·9 (1267·7–1610·9)	-505·3 (-642·3–-361·7)	-35·1 (-43·0–-26·9)	1340·9 (969·2–1600·1)	2301·6 (1888·8–2590·8)	-960·7 (-1402·0–-588·7)	-41·6 (-57·0–-29·0)
		Cirrhosis and other chronic liver diseases	1188·3 (974·4–1434·9)	2036·1 (1657·2–2439·6)	-847·9 (-1129·0–-575·9)	-41·5 (-49·3–-32·7)	2282·1 (1909·0–2689·2)	3379·3 (2841·2–3920·3)	-1097·2 (-1600·7–-454·5)	-32·2 (-45·1–-15·9)
		Depressive disorders	1496·5 (971·2–2134·9)	918·6 (597·6–1292·4)	577·9 (357·4–857·8)	63·0 (51·2–74·6)	1351·4 (892·6–1868·9)	841·2 (551·5–1177·6)	510·2 (329·0–736·9)	60·8 (50·6–70·9)
		Diabetes mellitus	3951·6 (3181·7–4969·1)	3815·4 (3058·9–4795·1)	136·2 (-68·7–419·3)	3·6 (-1·7–11·8)	2453·9 (2129·7–2881·6)	2147·2 (1841·6–2571·1)	306·6 (14·6–519·3)	14·5 (0·6–25·7)
		Falls	367·0 (273·4–475·5)	697·4 (552·9–884·3)	-330·4 (-411·2–-267·7)	-47·5 (-51·9–-42·6)	394·0 (303·8–502·4)	745·6 (603·7–914·6)	-351·6 (-445·1–-283·2)	-47·2 (-53·8–-41·3)
		HIV/AIDS	69·4 (43·5–129·5)	79·3 (53·2–141·7)	-10·0 (-48·1–21·5)	-11·5 (-44·6–26·9)	7·8 (5·4–15·0)	13·4 (10·1–20·7)	-5·5 (-8·9–-3·2)	-42·3 (-54·9–-20·6)
		Headache disorders	918·1 (275·0–1825·0)	548·0 (191·8–1053·5)	370·1 (82·2–771·8)	65·6 (33·8–94·4)	919·6 (275·8–1807·6)	551·6 (199·1–1055·7)	368·0 (81·2–776·4)	64·8 (34·4–92·6)
		Ischemic heart disease	6974·7 (5830·8–8202·3)	12257·7 (10718·7–13979·9)	-5283·1 (-6417·5–-4304·9)	-43·1 (-49·2–-37·1)	10943·5 (9703·0–12495·8)	19013·8 (17413·3–20777·0)	-8070·3 (-9864·6–-6040·0)	-42·4 (-49·0–-33·8)
		Low back pain	1983·5 (1346·9–2761·7)	1436·5 (978·1–2003·7)	547·0 (358·7–760·2)	38·2 (32·1–44·1)	1996·1 (1384·3–2776·1)	1561·9 (1059·3–2187·0)	434·2 (297·4–610·4)	27·9 (22·8–33·7)
		Lower respiratory infections	521·9 (402·0–623·2)	705·6 (604·3–821·8)	-183·7 (-313·0–-92·4)	-25·9 (-42·9–-14·5)	828·5 (700·4–1067·0)	1044·5 (913·7–1187·1)	-216·0 (-376·3–21·5)	-20·5 (-33·8–2·1)
		Other musculoskeletal disorders	1138·6 (759·0–1615·0)	694·5 (439·3–1024·4)	444·1 (317·7–612·6)	65·1 (50·0–81·8)	842·3 (558·8–1214·9)	451·8 (268·8–700·3)	390·5 (274·6–526·4)	88·7 (67·8–116·7)
		Road injuries	701·1 (599·4–807·7)	1867·5 (1670·4–2106·2)	-1166·4 (-1342·2–-1013·3)	-62·4 (-67·1–-59·1)	1205·7 (1068·6–1363·7)	3147·1 (2857·4–3495·2)	-1941·4 (-2198·1–-1714·8)	-61·7 (-65·8–-57·9)
		Stroke	3887·8 (3292·8–4581·0)	4233·1 (3605·5–4889·2)	-345·3 (-946·5–203·6)	-8·0 (-20·5–5·0)	7220·3 (6035·3–8340·4)	8039·7 (6947·2–8995·2)	-819·4 (-2009·1–349·6)	-10·0 (-24·4–4·6)
		Tracheal, bronchus, and lung cancer	506·5 (424·1–594·5)	2214·2 (1892·2–2609·9)	-1707·8 (-2066·8–-1413·4)	-77·1 (-80·8–-73·3)	425·2 (344·9–541·9)	3057·5 (2465·4–3701·1)	-2632·3 (-3276·3–-2022·6)	-85·9 (-89·5–-80·4)

		Tuberculosis	265·2 (206·3–334·8)	229·6 (152·0–416·7)	35·5 (-126·0–122·9)	20·8 (-30·3–70·7)	1062·4 (862·3–1279·2)	1070·0 (630·7–2244·3)	-7·6 (-1095·9–519·7)	7·6 (-49·5–82·8)
	70+ years old	Age-related and other hearing loss	2853·4 (2054·6–3931·5)	2970·5 (2136·7–4053·3)	-117·2 (-233·4–-12·3)	-3·9 (-7·2–-0·4)	2981·4 (2150·7–4046·7)	3057·8 (2197·4–4184·7)	-76·4 (-197·1–36·5)	-2·5 (-5·8–1·4)
		Alzheimer's disease and other dementias	6392·0 (3128·1–13786·9)	5016·7 (2382·2–11119·4)	1375·3 (730·0–2557·0)	28·5 (21·0–36·0)	6674·4 (3249·9–14839·9)	4845·3 (2307·0–11044·3)	1829·1 (905·9–3673·8)	38·7 (30·3–47·3)
		Anxiety disorders	624·2 (390·4–926·7)	330·2 (208·9–485·0)	294·0 (168·9–474·8)	89·4 (60·4–118·8)	575·9 (377·8–859·6)	313·8 (206·6–457·8)	262·1 (146·4–415·9)	83·9 (55·5–111·9)
		COVID-19	15687·9 (13852·6–17445·0)	29727·6 (25937·8–33048·0)	-14039·8 (-15543·3–-12214·2)	-47·2 (-48·3–-45·6)	NA	NA	NA	NA
		Chronic kidney disease	5905·7 (5147·5–6593·0)	6002·8 (5120·1–6974·3)	-97·1 (-984·2–821·9)	-1·2 (-14·4–15·6)	4603·3 (3789·0–6251·9)	4999·4 (3763·3–8794·1)	-396·1 (-4214·3–1524·5)	-3·6 (-48·4–39·0)
		Chronic obstructive pulmonary disease	4439·7 (3815·0–5026·4)	6256·4 (5596·3–6995·3)	-1816·7 (-2500·4–-1176·9)	-28·9 (-37·8–-20·2)	5118·3 (3563·4–6100·1)	7671·8 (6041·2–8810·3)	-2553·5 (-4188·7–-1141·6)	-33·0 (-52·0–-15·8)
		Cirrhosis and other chronic liver diseases	2787·4 (2406·9–3204·1)	3330·4 (2837·1–3913·8)	-543·0 (-975·6–-98·2)	-16·0 (-26·9–-3·4)	7507·5 (6058·8–9280·4)	6541·2 (5512·0–7842·8)	966·3 (-564·9–2752·6)	15·1 (-8·2–45·8)
		Depressive disorders	1037·3 (698·8–1464·7)	731·7 (491·7–1016·9)	305·5 (196·7–447·6)	41·8 (32·4–50·4)	1034·3 (704·4–1426·1)	734·1 (486·0–1019·4)	300·2 (196·3–426·7)	41·1 (33·1–49·3)
		Diabetes mellitus	8106·2 (6990·2–9739·7)	7501·2 (6284·0–9115·5)	605·0 (111·7–1239·7)	8·2 (1·4–17·5)	5347·5 (4685·6–6067·4)	4418·1 (3830·3–5239·5)	929·4 (343·9–1463·2)	21·3 (7·2–35·7)
		Falls	1288·8 (1052·8–1573·7)	1284·7 (1063·4–1579·2)	4·1 (-237·9–149·3)	0·6 (-16·0–12·0)	1232·0 (1000·2–1524·0)	1342·8 (1072·8–1779·6)	-110·7 (-507·3–73·0)	-7·5 (-29·8–5·8)
		HIV/AIDS	44·1 (13·1–111·0)	94·0 (18·0–339·1)	-49·9 (-245·4–6·7)	-39·8 (-80·2–21·9)	2·5 (1·2–6·8)	6·1 (2·6–17·4)	-3·6 (-11·5–-1·2)	-57·1 (-74·7–-36·6)
		Headache disorders	482·4 (169·4–960·3)	342·7 (126·5–671·1)	139·7 (37·6–289·5)	40·4 (23·3–57·0)	482·2 (171·1–956·6)	349·3 (128·5–700·6)	132·9 (37·0–276·3)	37·8 (21·0–53·8)
		Ischemic heart disease	27741·6 (23944·3–31165·0)	31343·0 (27910·5–34561·5)	-3601·5 (-6397·3–-543·7)	-11·4 (-20·2–-1·8)	36686·2 (32706·4–41821·7)	39547·6 (35246·5–43245·7)	-2861·4 (-7359·0–2401·0)	-7·1 (-17·5–6·4)
		Low back pain	2824·6 (1958·4–3825·8)	2275·8 (1550·7–3092·7)	548·8 (374·4–768·9)	24·2 (18·4–30·1)	2893·1 (1985·6–3919·5)	2333·4 (1592·9–3165·3)	559·7 (363·1–789·4)	24·1 (18·3–30·4)
		Lower respiratory infections	2476·8 (1928·0–2882·6)	3014·0 (2632·2–3390·4)	-537·2 (-1084·9–-140·5)	-17·7 (-35·9–-5·0)	3341·5 (2807·4–4572·8)	3646·3 (3155·2–4039·8)	-304·8 (-858·2–965·5)	-8·2 (-22·0–28·0)
		Other musculoskeletal disorders	906·3 (616·4–1353·6)	602·8 (383·0–951·3)	303·5 (206·6–421·4)	51·5 (38·6–67·5)	700·8 (454·4–1066·1)	382·4 (222·1–633·0)	318·4 (215·2–445·1)	86·4 (59·0–119·2)

		Road injuries	638·0 (549·6–736·9)	1742·5 (1567·1–1966·3)	-1104·5 (-1265·8–-965·0)	-63·4 (-67·8–-60·4)	1008·2 (867·2–1156·7)	2714·3 (2455·4–2992·4)	-1706·1 (-1941·9–-1492·1)	-62·8 (-67·3–-58·2)
		Stroke	17146·3 (14740·0–19555·5)	16449·2 (14595·9–18490·4)	697·1 (-1374·2–2902·2)	4·4 (-7·9–18·4)	26999·9 (22845·1–30920·7)	25548·4 (22348·9–28298·9)	1451·5 (-2580·3–4971·6)	5·8 (-9·5–20·8)
		Tracheal, bronchus, and lung cancer	943·1 (790·7–1093·1)	3688·2 (3175·6–4333·2)	-2745·1 (-3287·5–-2264·4)	-74·3 (-78·5–-70·1)	586·5 (452·8–730·4)	3792·7 (3095·8–4589·5)	-3206·2 (-3965·3–-2540·6)	-84·4 (-88·4–-79·3)
		Tuberculosis	411·8 (344·6–490·1)	482·6 (318·1–950·0)	-70·7 (-507·8–106·6)	-9·2 (-56·3–32·5)	1755·4 (1448·8–2070·9)	2014·4 (1043·0–5275·8)	-259·1 (-3515·5–787·5)	-0·2 (-66·5–71·2)
South Asia	10-24 years old	Age-related and other hearing loss	158·8 (98·3–236·6)	175·8 (109·3–256·4)	-17·0 (-33·4–-5·0)	-9·6 (-15·2–-3·1)	148·7 (92·5–220·6)	165·8 (101·1–247·1)	-17·1 (-32·3–-5·1)	-10·2 (-16·5–-3·7)
		Alzheimer's disease and other dementias	0·0 (0·0–0·0)	0·0 (0·0–0·0)	0·0 (0·0–0·0)	NA (NA–NA)	0·0 (0·0–0·0)	0·0 (0·0–0·0)	0·0 (0·0–0·0)	NA (NA–NA)
		Anxiety disorders	487·2 (312·6–702·1)	306·5 (194·0–443·0)	180·7 (115·5–261·7)	59·2 (50·2–69·2)	378·6 (244·7–544·5)	244·4 (154·9–359·7)	134·2 (87·0–195·0)	55·2 (45·6–66·0)
		COVID-19	548·7 (366·6–946·0)	566·3 (434·3–874·1)	-17·5 (-73·9–83·2)	-4·3 (-16·6–10·6)	NA	NA	NA	NA
		Chronic kidney disease	77·6 (66·1–92·9)	83·5 (68·8–104·3)	-6·0 (-22·3–9·1)	-6·5 (-22·7–12·6)	78·1 (65·7–94·3)	92·4 (75·0–110·2)	-14·2 (-32·1–2·2)	-14·9 (-30·9–2·7)
		Chronic obstructive pulmonary disease	38·7 (30·6–46·6)	39·7 (31·6–47·5)	-1·0 (-9·1–8·6)	-1·8 (-20·5–24·3)	43·4 (31·0–56·2)	46·4 (34·1–57·8)	-3·0 (-18·6–11·2)	-5·5 (-34·0–27·1)
		Cirrhosis and other chronic liver diseases	237·4 (185·0–297·8)	158·8 (123·2–201·5)	78·6 (10·5–140·1)	51·3 (5·5–97·3)	324·3 (256·1–404·1)	270·2 (216·0–335·2)	54·0 (-29·8–136·0)	21·3 (-10·1–57·2)
		Depressive disorders	609·6 (382·9–888·3)	411·7 (259·6–599·6)	197·9 (125·2–287·4)	48·2 (42·8–53·6)	508·3 (325·2–742·1)	329·5 (209·4–484·6)	178·8 (115·0–261·9)	54·4 (48·1–60·2)
		Diabetes mellitus	96·5 (75·0–122·7)	83·9 (62·6–114·0)	12·6 (3·7–21·8)	15·6 (4·0–30·0)	82·7 (69·1–100·9)	63·6 (51·7–79·4)	19·2 (10·1–31·0)	30·6 (14·9–52·0)
		Falls	166·9 (129·7–212·2)	233·9 (189·8–276·0)	-67·0 (-95·8–-35·2)	-28·6 (-39·5–-16·3)	227·3 (172·2–287·5)	340·4 (262·3–405·4)	-113·1 (-155·9–-54·7)	-33·1 (-43·2–-18·7)
		HIV/AIDS	49·3 (28·7–80·5)	37·8 (21·4–69·8)	11·5 (-7·3–27·7)	33·9 (-15·2–86·8)	0·7 (0·4–1·3)	1·1 (0·6–2·0)	-0·4 (-0·8–-0·2)	-35·4 (-47·0–-21·9)
		Headache disorders	795·6 (77·7–1883·1)	531·5 (51·5–1222·5)	264·1 (22·5–629·0)	49·9 (26·6–67·0)	787·2 (75·4–1848·1)	517·5 (51·6–1207·7)	269·7 (21·5–634·3)	52·0 (26·5–71·1)
		Ischemic heart disease	128·8 (110·5–153·5)	145·0 (124·3–167·1)	-16·1 (-44·6–15·3)	-10·6 (-28·0–11·9)	178·0 (131·5–228·0)	139·9 (113·6–169·4)	38·1 (-20·9–95·0)	28·9 (-12·7–75·3)
		Low back pain	456·1 (289·3–649·5)	225·4 (144·0–317·1)	230·7 (146·3–336·2)	102·6 (85·9–119·2)	493·7 (318·1–707·2)	252·0 (161·3–358·6)	241·7 (156·8–353·2)	96·1 (82·1–110·0)

		Lower respiratory infections	171·5 (136·4–217·2)	137·8 (115·8–160·4)	33·7 (-2·7–81·8)	25·3 (-1·7–63·2)	347·0 (280·4–448·2)	292·3 (247·3–341·0)	54·7 (-4·1–167·2)	19·4 (-1·3–62·4)
		Other musculoskeletal disorders	229·5 (149·3–336·3)	126·5 (80·2–187·2)	103·0 (69·1–150·5)	82·0 (70·6–94·8)	192·6 (127·6–277·3)	107·4 (68·2–158·9)	85·2 (56·5–120·2)	80·1 (68·1–93·6)
		Road injuries	209·2 (174·6–242·1)	1015·5 (903·3–1141·7)	-806·3 (-934·1–-688·8)	-79·3 (-82·9–-75·2)	274·9 (233·6–310·7)	1155·4 (1015·1–1294·7)	-880·6 (-1009·6–-744·2)	-76·2 (-79·6–-72·0)
		Stroke	94·8 (80·8–112·1)	80·8 (68·0–93·0)	14·0 (-3·9–34·0)	17·9 (-4·3–45·2)	138·2 (105·8–175·9)	113·3 (87·8–138·1)	24·8 (-16·6–67·9)	23·7 (-12·9–72·8)
		Tracheal, bronchus, and lung cancer	4·2 (3·0–6·3)	5·8 (4·6–7·6)	-1·6 (-3·3–1·1)	-25·3 (-50·6–22·4)	2·6 (2·1–3·2)	5·8 (4·7–7·3)	-3·2 (-4·7–-2·0)	-54·6 (-65·0–-40·5)
		Tuberculosis	424·6 (365·7–488·2)	329·8 (275·0–393·7)	94·8 (16·8–170·0)	29·7 (4·5–60·9)	1492·8 (1296·3–1703·8)	1064·6 (802·2–1283·6)	428·2 (184·0–731·6)	41·9 (14·5–89·9)
	25-49 years old	Age-related and other hearing loss	377·5 (246·0–549·1)	407·6 (255·5–609·5)	-30·2 (-69·2–-4·7)	-7·1 (-12·1–-1·6)	366·7 (239·7–523·5)	397·3 (253·2–588·6)	-30·6 (-66·2–-5·1)	-7·4 (-12·5–-1·7)
		Alzheimer's disease and other dementias	5·8 (2·7–12·4)	4·6 (2·1–10·1)	1·2 (-0·1–3·0)	27·5 (-3·0–64·8)	4·9 (2·4–10·4)	4·0 (1·8–8·5)	0·8 (-0·2–2·1)	22·1 (-5·5–58·4)
		Anxiety disorders	718·3 (492·9–992·0)	494·2 (334·8–690·8)	224·1 (149·6–314·0)	45·6 (37·7–54·3)	588·3 (396·3–822·7)	427·1 (282·4–602·5)	161·1 (106·1–231·2)	38·0 (30·4–45·6)
		COVID-19	1859·4 (1530·4–2500·6)	3270·6 (3046·3–3683·5)	-1411·2 (-1593·7–-1127·2)	-43·3 (-50·2–-31·5)	NA	NA	NA	NA
		Chronic kidney disease	317·1 (265·1–380·1)	396·4 (323·0–483·5)	-79·3 (-147·0–-8·7)	-19·6 (-33·2–-2·4)	292·2 (247·5–340·5)	356·6 (297·2–430·5)	-64·3 (-127·5–-1·9)	-17·6 (-31·7–-0·6)
		Chronic obstructive pulmonary disease	255·1 (208·6–304·4)	343·1 (264·5–394·4)	-88·0 (-164·1–9·3)	-25·0 (-42·2–3·4)	326·9 (220·0–420·8)	425·1 (279·1–515·0)	-98·2 (-216·4–22·3)	-22·1 (-47·4–5·7)
		Cirrhosis and other chronic liver diseases	443·7 (332·8–570·5)	1284·2 (1038·6–1568·9)	-840·5 (-1131·7–-621·2)	-65·3 (-74·8–-54·4)	630·2 (515·3–766·2)	1700·7 (1423·5–2120·9)	-1070·5 (-1468·3–-786·3)	-62·6 (-71·0–-53·4)
		Depressive disorders	1263·7 (851·6–1779·1)	905·9 (609·2–1276·2)	357·8 (232·2–510·4)	39·6 (34·5–44·5)	1241·3 (829·6–1742·8)	849·6 (572·1–1195·2)	391·7 (256·6–552·3)	46·2 (40·8–51·2)
		Diabetes mellitus	592·4 (465·0–745·4)	685·7 (526·9–879·1)	-93·3 (-151·5–-37·7)	-13·4 (-20·3–-6·1)	374·8 (314·7–447·4)	416·6 (340·8–501·3)	-41·8 (-76·6–-2·3)	-9·9 (-17·1–-0·6)
		Falls	320·6 (246·3–411·4)	603·5 (500·9–717·4)	-282·9 (-355·9–-218·8)	-46·9 (-55·5–-37·8)	387·6 (295·0–499·4)	700·8 (568·3–826·2)	-313·2 (-391·8–-236·8)	-44·8 (-52·9–-36·1)
		HIV/AIDS	263·8 (188·4–451·8)	255·3 (162·5–442·4)	8·4 (-134·4–115·4)	7·1 (-34·1–50·9)	2·1 (1·0–4·3)	5·0 (2·3–10·0)	-2·9 (-5·7–-1·3)	-57·2 (-61·3–-52·7)

		Headache disorders	1006·7 (165·1–2197·0)	707·4 (115·7–1516·2)	299·2 (45·9–670·2)	42·6 (22·9–55·5)	1005·7 (160·4–2193·4)	702·6 (117·6–1483·2)	303·1 (43·4–686·0)	43·1 (23·4–55·9)
		Ischemic heart disease	1021·9 (868·0–1175·5)	2311·7 (2032·4–2583·2)	-1289·8 (-1627·7–-973·6)	-55·6 (-63·5–-46·1)	1226·0 (999·7–1432·4)	2203·4 (1942·9–2487·7)	-977·3 (-1328·5–-617·2)	-44·1 (-56·3–-30·9)
		Low back pain	1183·7 (807·5–1635·7)	491·1 (332·7–688·3)	692·5 (474·9–957·9)	141·4 (129·4–154·5)	1307·9 (891·3–1816·9)	623·4 (421·3–857·0)	684·6 (470·2–947·1)	110·0 (99·8–121·5)
		Lower respiratory infections	182·0 (148·5–250·8)	211·8 (178·3–247·7)	-29·8 (-76·0–33·9)	-13·6 (-32·6–16·3)	318·1 (264·7–428·2)	337·8 (302·4–369·8)	-19·7 (-75·9–75·2)	-5·7 (-21·7–22·2)
		Other musculoskeletal disorders	1324·1 (907·8–1920·4)	849·9 (571·0–1267·0)	474·3 (333·2–665·9)	56·2 (49·7–62·4)	1068·1 (742·7–1553·1)	725·9 (496·2–1070·6)	342·2 (243·6–485·9)	47·4 (42·1–52·7)
		Road injuries	331·1 (282·6–381·7)	1740·4 (1562·2–1905·8)	-1409·3 (-1568·0–-1229·6)	-80·9 (-84·1–-77·6)	408·9 (361·3–454·5)	1671·8 (1513·8–1819·9)	-1262·8 (-1395·6–-1099·7)	-75·5 (-78·6–-71·9)
		Stroke	511·1 (435·1–592·3)	679·9 (587·8–769·4)	-168·8 (-283·0–-60·6)	-24·5 (-38·1–-9·6)	726·5 (619·9–840·1)	889·6 (756·0–1009·3)	-163·0 (-328·9–23·0)	-17·9 (-33·9–2·9)
		Tracheal, bronchus, and lung cancer	54·2 (45·6–63·4)	107·4 (87·2–128·5)	-53·2 (-74·7–-31·1)	-49·0 (-59·9–-34·2)	32·5 (27·4–37·9)	100·2 (85·4–120·1)	-67·7 (-85·9–-53·1)	-67·4 (-73·0–-60·0)
		Tuberculosis	854·0 (733·0–998·0)	1455·0 (1208·9–1780·3)	-601·0 (-932·2–-337·6)	-40·8 (-54·6–-26·1)	2990·0 (2653·4–3343·3)	4224·3 (3346·8–5049·0)	-1234·3 (-2123·5–-253·2)	-28·6 (-42·4–-7·4)
	50-69 years old	Age-related and other hearing loss	1218·3 (807·7–1755·4)	1269·8 (831·5–1852·8)	-51·5 (-119·0–-3·2)	-3·9 (-7·2–-0·3)	1222·4 (816·6–1759·1)	1277·1 (852·3–1857·1)	-54·7 (-123·3–-7·1)	-4·1 (-7·6–-0·7)
		Alzheimer's disease and other dementias	427·1 (179·3–1049·6)	292·5 (128·9–696·5)	134·5 (45·1–348·5)	45·3 (28·6–64·5)	372·4 (161·5–873·5)	261·4 (118·1–624·5)	111·1 (38·1–273·5)	41·9 (26·0–59·0)
		Anxiety disorders	626·4 (430·2–877·6)	464·1 (305·6–664·4)	162·3 (95·7–242·0)	35·4 (21·3–48·5)	548·7 (370·5–764·0)	422·8 (282·4–597·0)	125·9 (67·0–199·9)	30·2 (16·4–42·6)
		COVID-19	5629·7 (5252·9–6262·1)	11805·7 (11299·6–12437·5)	-6176·1 (-6574·9–-5826·7)	-52·3 (-54·0–-49·2)	NA	NA	NA	NA
		Chronic kidney disease	1145·5 (970·0–1345·0)	1526·8 (1232·2–1851·0)	-381·2 (-687·2–-80·4)	-24·3 (-39·8–-5·9)	996·5 (861·1–1169·8)	1391·1 (1125·6–1625·9)	-394·6 (-649·9–-93·9)	-27·8 (-41·1–-8·4)
		Chronic obstructive pulmonary disease	3671·3 (2930·9–4376·1)	5278·3 (4271·1–6080·3)	-1607·0 (-2770·6–-241·7)	-29·9 (-47·6–-5·4)	4712·3 (3055·3–5902·6)	7068·4 (4840·6–8204·9)	-2356·2 (-4389·8–-451·0)	-32·7 (-57·6–-9·0)
		Cirrhosis and other chronic liver diseases	1085·0 (790·6–1404·4)	2383·4 (1781·6–2997·8)	-1298·4 (-1938·3–-834·8)	-54·1 (-67·7–-38·6)	1692·1 (1299·2–2189·8)	3267·6 (2621·7–4321·6)	-1575·5 (-2453·1–-835·6)	-47·7 (-59·4–-30·0)

		Depressive disorders	1547·1 (1027·9–2092·9)	1135·5 (749·2–1560·9)	411·6 (269·6–572·4)	36·4 (30·6–41·7)	1549·4 (1031·7–2125·7)	1085·2 (733·3–1503·8)	464·2 (301·0–650·6)	42·8 (37·8–48·2)
		Diabetes mellitus	3188·9 (2727·8–3796·9)	3259·9 (2685·8–3888·1)	-71·0 (-476·4–330·1)	-1·9 (-13·4–10·7)	2141·9 (1867·7–2427·8)	2269·9 (1936·7–2569·5)	-128·0 (-400·1–129·8)	-5·4 (-17·2–6·7)
		Falls	1275·2 (1033·0–1505·3)	1283·2 (1064·4–1539·7)	-8·0 (-242·3–189·1)	-0·3 (-17·2–16·2)	1415·9 (1122·7–1703·4)	1505·0 (1251·2–1773·4)	-89·1 (-359·0–171·3)	-5·7 (-24·1–12·2)
		HIV/AIDS	134·3 (88·6–204·5)	186·9 (103·1–322·5)	-52·5 (-179·2–29·7)	-23·6 (-57·8–25·7)	1·8 (1·1–2·7)	2·9 (1·8–4·5)	-1·1 (-2·0–-0·5)	-37·6 (-49·6–-25·7)
		Headache disorders	809·7 (208·3–1682·1)	554·8 (145·3–1154·3)	254·9 (63·2–543·2)	46·3 (29·0–59·2)	816·9 (210·8–1670·5)	556·5 (144·7–1145·3)	260·3 (62·4–553·2)	47·1 (28·8–62·7)
		Ischemic heart disease	6500·1 (5777·4–7349·1)	12223·3 (10776·6–13818·9)	-5723·2 (-7516·2–-3990·6)	-46·6 (-55·2–-35·9)	7295·5 (6304·7–8231·2)	11632·3 (10446·0–12984·2)	-4336·8 (-5929·0–-2838·9)	-37·1 (-47·6–-26·8)
		Low back pain	2348·5 (1605·9–3284·3)	1097·0 (736·6–1536·5)	1251·5 (852·2–1739·5)	114·4 (104·5–125·0)	2433·0 (1671·1–3408·8)	1246·9 (849·6–1739·6)	1186·2 (813·3–1650·6)	95·4 (85·9–105·7)
		Lower respiratory infections	1106·9 (880·7–1352·0)	1175·5 (982·0–1412·2)	-68·6 (-360·8–208·5)	-5·3 (-27·6–19·2)	1626·2 (1275·3–2088·9)	1651·2 (1456·0–1834·6)	-25·0 (-370·8–444·6)	-1·2 (-22·0–27·8)
		Other musculoskeletal disorders	2034·3 (1398·7–2831·8)	1312·4 (878·8–1872·8)	721·9 (512·9–965·7)	55·4 (48·2–63·2)	1617·0 (1099·8–2261·1)	1098·3 (735·9–1561·0)	518·6 (352·5–696·7)	47·5 (41·6–53·7)
		Road injuries	528·9 (454·2–607·0)	1436·4 (1269·0–1617·8)	-907·5 (-1091·4–-732·9)	-63·1 (-69·1–-56·5)	560·5 (490·5–627·7)	1339·0 (1188·1–1475·1)	-778·5 (-910·0–-635·7)	-58·1 (-63·8–-52·4)
		Stroke	4412·0 (3848·5–5007·5)	5501·1 (4816·9–6273·9)	-1089·2 (-2014·8–-192·6)	-19·5 (-33·3–-3·9)	6187·1 (5209·5–6942·9)	7433·5 (6437·3–8321·3)	-1246·4 (-2470·1–11·3)	-16·4 (-30·6–0·2)
		Tracheal, bronchus, and lung cancer	342·5 (288·5–408·2)	948·1 (773·4–1109·7)	-605·6 (-763·4–-453·6)	-63·7 (-70·1–-55·9)	207·6 (174·2–237·7)	937·3 (801·1–1111·5)	-729·7 (-895·9–-598·8)	-77·7 (-81·6–-73·1)
		Tuberculosis	1597·5 (1347·9–1864·6)	3020·3 (2485·8–3823·2)	-1422·8 (-2240·1–-854·4)	-46·5 (-60·2–-32·6)	5876·7 (5068·2–6705·3)	10404·2 (8345·9–12121·9)	-4527·5 (-6479·5–-2418·6)	-43·1 (-54·2–-28·3)
	70+ years old	Age-related and other hearing loss	2936·9 (2129·7–3991·2)	3049·4 (2184·4–4145·9)	-112·4 (-226·4–-10·4)	-3·6 (-6·6–-0·4)	2965·5 (2152·2–4018·5)	3115·7 (2246·4–4243·2)	-150·2 (-278·9–-40·1)	-4·8 (-7·7–-1·5)
		Alzheimer's disease and other dementias	3935·8 (1744·7–8861·5)	2779·2 (1255·9–6442·2)	1156·6 (446·9–2641·8)	41·7 (28·8–58·3)	3168·9 (1496·3–7279·0)	2455·8 (1167·5–5661·2)	713·0 (273·4–1730·1)	29·6 (14·9–46·5)
		Anxiety disorders	452·3 (308·1–634·3)	348·6 (236·0–491·4)	103·7 (51·6–166·4)	30·0 (16·6–43·0)	426·4 (293·9–601·6)	326·3 (224·1–455·1)	100·0 (51·4–162·9)	30·9 (15·3–44·5)

		COVID-19	14655·9 (14021·4–15319·6)	27125·3 (26077·6–28443·1)	-12469·4 (-13175·2–-11952·4)	-46·0 (-46·5–-45·2)	NA	NA	NA	NA
		Chronic kidney disease	2352·4 (2026·9–2761·3)	3138·2 (2564·9–3732·0)	-785·8 (-1374·0–-227·9)	-24·5 (-39·4–-7·7)	1956·2 (1630·1–2294·7)	2603·8 (2125·9–3101·0)	-647·6 (-1136·8–-109·3)	-24·4 (-37·3–-5·3)
		Chronic obstructive pulmonary disease	17307·6 (13733·8–20447·9)	21712·3 (18260·6–24572·4)	-4404·7 (-9007·9–909·9)	-19·8 (-38·7–4·9)	17433·7 (11776·7–22160·4)	23959·5 (17803·1–28142·5)	-6525·8 (-13254·6–130·9)	-26·5 (-52·2–0·7)
		Cirrhosis and other chronic liver diseases	1350·4 (929·8–1796·2)	2363·1 (1699·1–2989·2)	-1012·7 (-1711·9–-497·1)	-42·5 (-60·7–-22·9)	2039·3 (1345·8–2930·8)	2387·1 (1824·4–3324·2)	-347·8 (-1014·5–528·9)	-14·0 (-40·7–25·7)
		Depressive disorders	1404·7 (965·8–1912·9)	1132·1 (764·3–1563·4)	272·7 (186·0–374·3)	24·2 (19·4–28·8)	1361·2 (932·0–1852·0)	1076·6 (735·5–1484·5)	284·6 (194·6–385·4)	26·5 (22·0–31·6)
		Diabetes mellitus	6138·5 (5359·9–7011·3)	6674·1 (5734·9–7647·9)	-535·6 (-1504·4–335·3)	-7·7 (-20·7–5·5)	4028·3 (3458·2–4557·8)	4675·1 (3994·2–5374·4)	-646·8 (-1297·6–51·8)	-13·5 (-26·5–1·2)
		Falls	5472·9 (4545·4–6439·9)	3738·0 (3179·5–4302·7)	1734·9 (818·7–2613·6)	46·9 (20·4–73·9)	5230·1 (4213·9–6310·7)	3797·8 (3035·4–4576·9)	1432·4 (496·4–2403·3)	38·6 (12·7–73·9)
		HIV/AIDS	24·7 (14·6–41·4)	39·9 (21·7–74·6)	-15·3 (-48·8–5·3)	-32·8 (-68·9–19·2)	0·2 (0·1–0·4)	0·5 (0·3–0·9)	-0·3 (-0·7–-0·1)	-56·3 (-80·5–-18·9)
		Headache disorders	430·1 (136·2–884·0)	324·0 (105·4–680·0)	106·1 (29·2–240·6)	32·8 (16·4–46·9)	433·8 (139·3–889·5)	322·0 (104·9–661·8)	111·8 (29·6–232·1)	34·9 (15·8–47·8)
		Ischemic heart disease	16120·9 (14311·2–17776·7)	24170·9 (21685·5–26968·1)	-8050·1 (-11180·7–-5021·8)	-33·1 (-43·0–-22·3)	15648·8 (12858·0–17901·7)	18717·3 (16122·7–21187·9)	-3068·4 (-6667·3–299·6)	-16·0 (-32·7–1·9)
		Low back pain	2513·4 (1738·5–3417·9)	1372·0 (925·8–1871·0)	1141·4 (795·2–1527·8)	83·5 (74·3–93·2)	3032·4 (2089·7–4105·8)	1549·2 (1049·3–2121·9)	1483·2 (1047·9–1986·4)	96·2 (83·5–108·5)
		Lower respiratory infections	4221·7 (3276·2–5437·4)	4699·2 (3906·4–5523·8)	-477·4 (-1654·4–688·2)	-9·6 (-33·0–15·0)	5091·0 (3850·2–6915·7)	5233·4 (4312·1–6006·0)	-142·3 (-1429·3–1712·5)	-2·3 (-26·0–34·6)
		Other musculoskel etal disorders	1581·1 (1111·4–2237·0)	1323·5 (933·4–1866·1)	257·6 (70·8–446·4)	19·6 (5·2–33·1)	1259·6 (854·4–1819·8)	1061·6 (741·7–1508·9)	198·0 (26·0–374·7)	18·7 (2·9–33·2)
		Road injuries	466·0 (402·6–527·5)	1156·5 (1032·1–1285·5)	-690·5 (-818·5–-566·7)	-59·6 (-66·2–-53·6)	489·0 (407·9–559·6)	964·8 (833·7–1081·2)	-475·8 (-588·5–-345·4)	-49·2 (-57·2–-39·8)
		Stroke	11422·8 (10015·2–12918·1)	13521·3 (12060·1–15119·5)	-2098·4 (-3989·2–-330·3)	-15·3 (-27·4–-2·5)	14892·0 (12398·4–17178·0)	16200·8 (13726·8–18174·6)	-1308·9 (-4789·4–1721·6)	-7·6 (-27·7–11·9)
		Tracheal, bronchus, and lung cancer	426·1 (362·2–494·8)	1136·7 (935·3–1310·0)	-710·5 (-884·5–-526·2)	-62·3 (-68·8–-54·6)	244·4 (190·3–289·9)	1086·0 (902·9–1284·5)	-841·6 (-1029·3–-658·2)	-77·4 (-81·7–-72·5)

		Tuberculosis	2647·2 (2271·2–3085·2)	4266·3 (3515·6–5465·0)	-1619·1 (-2824·6–-840·2)	-37·2 (-52·7–-22·6)	8764·8 (7100·2–10540·0)	13126·2 (9726·7–16078·1)	-4361·5 (-7820·1–-940·5)	-32·3 (-50·1–-9·5)
Southeast Asia, East Asia, and Oceania	10-24 years old	Age-related and other hearing loss	156·8 (100·6–228·6)	202·2 (128·0–291·4)	-45·4 (-66·4–-27·5)	-22·4 (-25·1–-19·2)	141·2 (90·9–205·8)	176·1 (111·7–255·2)	-34·9 (-52·8–-20·6)	-19·7 (-23·7–-15·6)
		Alzheimer's disease and other dementias	0·0 (0·0–0·0)	0·0 (0·0–0·0)	0·0 (0·0–0·0)	NA (NA–NA)	0·0 (0·0–0·0)	0·0 (0·0–0·0)	0·0 (0·0–0·0)	NA (NA–NA)
		Anxiety disorders	709·7 (451·3–1051·5)	425·3 (270·9–628·2)	284·4 (178·5–413·9)	67·0 (59·2–75·2)	624·0 (403·9–907·6)	385·8 (246·7–565·8)	238·1 (152·7–347·9)	61·8 (54·0–69·7)
		COVID-19	199·3 (121·7–339·5)	193·3 (127·9–302·9)	6·0 (-15·6–39·3)	2·6 (-8·6–17·0)	NA	NA	NA	NA
		Chronic kidney disease	106·2 (90·5–122·9)	119·7 (92·4–139·5)	-13·5 (-30·9–11·5)	-10·7 (-24·0–10·9)	140·9 (120·5–161·3)	145·7 (107·6–171·0)	-4·8 (-30·6–25·3)	-2·4 (-19·4–21·2)
		Chronic obstructive pulmonary disease	34·6 (28·6–42·3)	35·6 (31·2–40·3)	-1·0 (-6·3–5·4)	-2·8 (-17·0–15·1)	65·4 (52·0–75·9)	73·4 (62·9–85·6)	-8·0 (-26·5–7·0)	-10·4 (-33·1–10·5)
		Cirrhosis and other chronic liver diseases	66·0 (53·3–87·8)	99·3 (79·3–120·4)	-33·3 (-57·2–-9·0)	-33·0 (-48·8–-11·0)	126·1 (97·2–159·3)	153·9 (115·3–196·6)	-27·8 (-58·6–15·4)	-17·1 (-34·2–13·2)
		Depressive disorders	372·7 (236·4–540·9)	253·9 (161·7–374·1)	118·8 (75·3–171·0)	47·0 (41·2–53·5)	447·9 (286·1–647·1)	246·4 (157·6–359·2)	201·5 (130·1–288·9)	81·9 (75·2–88·9)
		Diabetes mellitus	83·0 (62·5–112·4)	85·8 (61·8–116·7)	-2·7 (-9·9–4·0)	-2·8 (-9·8–6·1)	68·4 (55·1–86·5)	55·5 (42·3–71·7)	12·9 (6·5–20·3)	23·9 (10·1–40·5)
		Falls	109·0 (84·5–139·7)	251·6 (211·0–298·2)	-142·6 (-180·8–-115·6)	-56·7 (-64·7–-49·2)	145·0 (114·4–186·4)	347·4 (291·2–439·8)	-202·5 (-278·5–-161·0)	-58·2 (-66·7–-51·2)
		HIV/AIDS	41·9 (36·9–48·2)	69·2 (62·8–77·2)	-27·4 (-35·2–-20·6)	-39·4 (-47·3–-30·6)	13·9 (13·0–14·8)	23·2 (22·2–24·5)	-9·4 (-9·9–-8·9)	-40·2 (-41·8–-38·8)
		Headache disorders	713·3 (67·1–1696·6)	451·6 (55·3–1042·2)	261·7 (8·6–657·4)	54·6 (14·6–81·4)	670·8 (68·4–1611·4)	412·4 (57·4–945·6)	258·4 (8·3–665·7)	59·0 (13·0–86·9)
		Ischemic heart disease	73·7 (63·0–86·9)	135·9 (117·2–158·9)	-62·2 (-85·8–-41·0)	-45·5 (-56·5–-33·5)	102·6 (84·7–120·5)	140·3 (123·0–159·4)	-37·7 (-64·5–-13·0)	-26·6 (-41·8–-10·4)
		Low back pain	342·0 (217·1–492·9)	211·1 (134·6–301·3)	130·9 (83·6–193·8)	62·1 (53·3–71·9)	363·9 (230·7–528·4)	206·6 (131·0–294·8)	157·3 (100·0–231·9)	76·2 (64·5–90·2)
		Lower respiratory infections	78·9 (67·4–98·8)	111·1 (99·9–124·2)	-32·2 (-47·9–-12·1)	-28·9 (-40·2–-11·7)	209·8 (147·5–239·7)	280·2 (244·7–314·3)	-70·3 (-136·7–-26·9)	-24·9 (-47·8–-9·8)
		Other musculoskeletal disorders	152·1 (109·6–208·1)	92·4 (58·7–138·0)	59·7 (46·8–73·0)	66·8 (46·8–97·1)	139·5 (103·6–187·0)	75·7 (48·4–115·1)	63·9 (51·4–78·5)	87·8 (60·5–132·3)
		Road injuries	337·8 (293·8–384·6)	1300·3 (1158·4–1471·1)	-962·5 (-1121·3–-820·4)	-74·0 (-77·7–-69·4)	595·7 (528·2–696·9)	1742·4 (1562·3–1925·0)	-1146·7 (-1348·2–-931·3)	-65·7 (-70·5–-59·1)

		Stroke	127·3 (107·6–146·8)	171·0 (148·7–193·3)	-43·7 (-65·5–-21·9)	-25·4 (-35·7–-13·5)	214·9 (186·2–245·3)	257·5 (227·5–289·8)	-42·5 (-81·8–-1·8)	-16·3 (-29·5–-0·7)
		Tracheal, bronchus, and lung cancer	11·8 (9·6–14·2)	19·2 (15·3–23·3)	-7·4 (-11·2–-3·9)	-38·2 (-51·1–-24·6)	16·3 (13·3–20·2)	26·9 (22·3–32·7)	-10·6 (-17·0–-4·9)	-38·8 (-53·9–-21·2)
		Tuberculosis	155·4 (132·0–186·3)	203·5 (171·6–247·2)	-48·2 (-85·8–-10·8)	-23·3 (-36·6–-5·5)	506·5 (446·5–560·7)	464·2 (352·0–535·5)	42·3 (-45·4–155·1)	10·0 (-8·8–44·0)
	25-49 years old	Age-related and other hearing loss	382·2 (249·8–557·1)	438·2 (278·0–644·5)	-55·9 (-91·4–-29·5)	-12·7 (-15·4–-10·0)	336·5 (218·3–485·8)	387·5 (246·4–564·3)	-51·0 (-81·4–-26·4)	-13·0 (-15·7–-9·8)
		Alzheimer's disease and other dementias	9·8 (4·9–19·6)	8·2 (3·9–16·9)	1·6 (-0·7–4·3)	21·9 (-8·6–62·4)	6·6 (3·3–13·3)	5·7 (2·8–11·9)	0·8 (-0·5–2·4)	16·0 (-9·3–50·2)
		Anxiety disorders	653·7 (441·2–927·4)	375·8 (248·6–537·4)	277·8 (183·4–396·3)	74·1 (64·8–83·2)	581·1 (390·8–820·6)	341·6 (225·3–484·7)	239·5 (161·9–336·1)	70·2 (62·8–78·3)
		COVID-19	430·4 (293·7–648·7)	778·7 (544·6–1203·1)	-348·2 (-566·3–-226·5)	-44·5 (-49·8–-35·3)	NA	NA	NA	NA
		Chronic kidney disease	294·0 (254·4–334·7)	384·7 (317·1–443·0)	-90·6 (-148·3–-33·7)	-23·2 (-35·5–-9·9)	357·6 (307·9–409·5)	380·0 (297·8–442·5)	-22·4 (-82·3–48·8)	-5·3 (-20·7–13·7)
		Chronic obstructive pulmonary disease	166·6 (138·5–199·5)	215·4 (187·4–244·7)	-48·9 (-85·0–-8·1)	-22·4 (-36·3–-3·9)	375·3 (280·6–450·3)	441·6 (378·7–514·6)	-66·3 (-195·4–24·9)	-14·5 (-40·8–6·3)
		Cirrhosis and other chronic liver diseases	182·3 (153·6–218·1)	759·7 (648·6–893·9)	-577·4 (-724·2–-467·7)	-75·9 (-81·1–-70·0)	384·1 (311·2–464·1)	1180·6 (1020·6–1418·0)	-796·5 (-1004·3–-597·5)	-67·3 (-73·9–-57·4)
		Depressive disorders	687·4 (469·9–943·2)	449·6 (303·4–627·2)	237·8 (161·0–329·6)	53·0 (48·4–57·9)	782·0 (527·1–1079·7)	461·4 (310·8–639·5)	320·6 (215·3–445·5)	69·6 (64·3–75·1)
		Diabetes mellitus	478·6 (364·8–621·3)	599·5 (455·0–776·2)	-120·9 (-168·4–-82·6)	-20·1 (-23·9–-15·4)	304·1 (245·3–377·3)	330·2 (260·9–415·4)	-26·1 (-54·7–3·9)	-7·7 (-15·0–1·2)
		Falls	234·2 (172·6–309·5)	606·5 (498·1–739·6)	-372·3 (-449·3–-290·3)	-61·4 (-68·1–-54·2)	246·7 (191·6–319·5)	632·0 (519·5–788·2)	-385·2 (-516·8–-317·8)	-60·9 (-67·7–-54·9)
		HIV/AIDS	143·2 (126·2–164·4)	368·4 (324·4–439·9)	-225·2 (-279·3–-188·9)	-61·0 (-65·8–-57·4)	49·9 (45·3–54·3)	145·5 (134·6–156·0)	-95·6 (-102·1–-88·8)	-65·7 (-67·0–-64·5)
		Headache disorders	987·7 (152·8–2120·7)	646·2 (147·4–1352·0)	341·4 (4·4–823·0)	48·8 (3·3–77·9)	930·6 (138·3–1981·1)	595·4 (136·4–1243·4)	335·2 (3·0–798·5)	52·0 (1·9–82·1)
		Ischemic heart disease	487·9 (418·5–565·1)	1328·2 (1148·0–1529·5)	-840·2 (-1047·6–-658·6)	-63·1 (-69·8–-56·3)	646·9 (552·0–739·8)	1056·8 (936·0–1192·8)	-409·8 (-577·6–-255·2)	-38·6 (-49·6–-26·3)
		Low back pain	820·0 (558·7–1128·6)	609·4 (415·2–844·4)	210·6 (142·5–300·4)	34·6 (30·0–39·6)	944·5 (633·8–1307·1)	643·9 (436·2–886·0)	300·5 (203·8–430·8)	46·8 (41·2–52·9)

		Lower respiratory infections	85·8 (72·3–104·4)	166·5 (148·4–187·3)	-80·7 (-104·7–-60·0)	-48·4 (-58·3–-38·3)	186·3 (139·3–218·9)	279·9 (246·1–313·5)	-93·5 (-147·7–-49·5)	-33·2 (-51·7–-19·3)
		Other musculoskeletal disorders	768·3 (529·4–1111·3)	562·5 (373·3–830·4)	205·8 (151·8–278·5)	37·1 (31·7–44·1)	626·3 (434·4–898·1)	440·6 (288·0–656·6)	185·8 (139·6–246·8)	42·9 (35·3–51·9)
		Road injuries	421·2 (348·6–498·0)	1715·2 (1498·9–1951·8)	-1294·0 (-1542·5–-1082·8)	-75·3 (-80·1–-70·0)	687·6 (596·8–823·9)	2196·9 (1968·1–2432·7)	-1509·3 (-1760·8–-1259·4)	-68·6 (-73·7–-61·9)
		Stroke	740·9 (637·7–847·3)	1365·3 (1172·2–1585·3)	-624·5 (-832·5–-432·2)	-45·5 (-54·7–-35·5)	1220·7 (1071·5–1385·7)	1574·8 (1379·2–1771·0)	-354·1 (-614·7–-114·4)	-22·2 (-35·4–-7·5)
		Tracheal, bronchus, and lung cancer	225·6 (178·9–280·4)	395·1 (307·0–493·6)	-169·5 (-277·2–-82·6)	-42·2 (-57·0–-24·6)	223·5 (181·6–274·2)	405·3 (333·7–488·9)	-181·8 (-283·0–-93·6)	-44·3 (-58·9–-27·0)
		Tuberculosis	205·9 (179·8–241·0)	406·8 (344·6–488·6)	-200·9 (-285·9–-141·6)	-49·1 (-59·3–-39·1)	849·5 (760·0–941·3)	1149·6 (854·9–1315·3)	-300·1 (-483·8–-7·8)	-25·4 (-37·5–-0·9)
	50-69 years old	Age-related and other hearing loss	1387·3 (893·6–2046·7)	1510·0 (980·2–2208·8)	-122·7 (-197·2–-64·9)	-8·1 (-10·9–-5·5)	1281·9 (820·9–1927·8)	1394·7 (897·4–2081·2)	-112·8 (-183·6–-60·9)	-8·1 (-10·8–-5·6)
		Alzheimer's disease and other dementias	591·8 (275·6–1317·2)	472·1 (216·7–1106·3)	119·7 (27·8–265·8)	27·0 (4·5–48·4)	577·8 (262·8–1306·3)	449·8 (195·9–1077·0)	128·0 (40·2–292·2)	29·9 (11·4–48·4)
		Anxiety disorders	636·2 (426·1–895·0)	388·9 (253·5–554·9)	247·3 (155·8–358·2)	64·1 (46·7–81·1)	601·7 (408·5–847·2)	373·5 (247·6–530·2)	228·2 (143·2–337·8)	61·5 (45·3–78·5)
		COVID-19	929·7 (645·7–1469·2)	1930·9 (1344·7–3092·1)	-1001·1 (-1623·0–-684·0)	-51·8 (-53·0–-49·6)	NA	NA	NA	NA
		Chronic kidney disease	891·8 (772·2–1021·9)	1052·1 (888·7–1222·5)	-160·3 (-329·4–14·2)	-14·8 (-28·9–1·6)	1116·2 (967·3–1283·7)	1096·8 (904·1–1326·1)	19·4 (-190·1–242·5)	2·5 (-15·4–25·3)
		Chronic obstructive pulmonary disease	1322·2 (1101·8–1614·1)	2213·0 (1845·1–2601·4)	-890·8 (-1354·5–-405·0)	-39·8 (-53·1–-22·2)	4720·5 (3373·7–5625·7)	6651·3 (5572·0–7917·5)	-1930·8 (-3827·5–-531·9)	-28·4 (-50·3–-8·9)
		Cirrhosis and other chronic liver diseases	514·0 (426·6–624·6)	1443·5 (1217·8–1705·2)	-929·6 (-1221·5–-697·8)	-64·1 (-72·8–-55·2)	1431·2 (1143·1–1727·9)	2811·1 (2322·1–3460·9)	-1379·9 (-1976·2–-780·7)	-48·6 (-60·0–-31·9)
		Depressive disorders	1030·9 (712·7–1409·4)	672·9 (466·6–925·7)	358·0 (245·2–493·3)	53·2 (47·8–58·9)	919·2 (633·5–1237·1)	589·5 (408·2–795·1)	329·7 (225·0–453·0)	56·0 (50·3–61·8)
		Diabetes mellitus	1940·8 (1608·2–2348·6)	2061·6 (1675·3–2532·5)	-120·8 (-292·0–44·4)	-5·8 (-13·0–2·3)	1830·7 (1562·0–2145·7)	1549·3 (1316·1–1842·5)	281·4 (103·3–481·8)	18·4 (6·6–32·8)
		Falls	520·4 (399·1–677·5)	969·6 (775·3–1218·4)	-449·2 (-567·0–-327·0)	-46·3 (-53·6–-38·9)	586·3 (467·7–725·1)	994·6 (807·1–1232·7)	-408·3 (-608·9–-304·6)	-40·9 (-52·1–-33·1)

		HIV/AIDS	82·4 (66·2–106·6)	218·6 (174·8–269·6)	-136·2 (-173·4–-105·5)	-62·3 (-66·2–-57·7)	8·2 (4·7–10·7)	20·3 (10·7–27·0)	-12·1 (-16·4–-6·1)	-59·1 (-61·5–-56·1)
		Headache disorders	785·6 (173·9–1676·8)	512·7 (158·0–1039·8)	272·9 (4·4–672·2)	48·8 (2·3–80·3)	750·0 (173·4–1599·8)	490·4 (157·5–984·8)	259·6 (6·8–627·5)	48·6 (3·6–79·6)
		Ischemic heart disease	2632·3 (2231·2–3083·1)	5271·1 (4457·5–6238·6)	-2638·9 (-3660·6–-1779·0)	-49·7 (-60·2–-38·1)	3800·0 (3344·0–4374·9)	5120·2 (4445·1–5835·4)	-1320·2 (-2252·6–-425·0)	-25·4 (-39·3–-9·4)
		Low back pain	1755·2 (1198·9–2460·4)	976·3 (665·8–1378·8)	778·9 (531·5–1083·7)	79·9 (73·1–87·7)	2090·3 (1425·5–2939·4)	1150·1 (779·5–1617·2)	940·3 (639·6–1328·9)	81·9 (73·7–90·4)
		Lower respiratory infections	270·0 (217·0–313·3)	526·1 (465·5–599·8)	-256·1 (-336·0–-186·9)	-48·5 (-59·5–-39·2)	623·1 (462·8–724·8)	962·0 (821·6–1113·4)	-338·9 (-551·7–-169·0)	-34·9 (-53·5–-20·3)
		Other musculoskeletal disorders	1067·4 (702·6–1506·6)	747·7 (479·5–1094·3)	319·6 (224·2–438·8)	43·2 (36·5–50·3)	897·7 (591·4–1275·7)	620·4 (396·0–910·4)	277·3 (192·5–374·7)	45·2 (38·7–53·6)
		Road injuries	554·5 (462·6–651·1)	1412·0 (1187·3–1645·2)	-857·5 (-1098·6–-638·1)	-60·5 (-68·1–-52·6)	862·6 (746·1–1019·5)	1748·4 (1524·4–1970·6)	-885·8 (-1117·6–-627·5)	-50·5 (-58·5–-41·7)
		Stroke	4661·2 (4007·4–5385·8)	7625·0 (6418·6–8953·4)	-2963·9 (-4442·5–-1655·4)	-38·4 (-50·8–-25·4)	10087·9 (8829·4–11623·0)	12464·6 (10652·0–14374·7)	-2376·7 (-4882·1–-267·2)	-18·6 (-33·3–-2·5)
		Tracheal, bronchus, and lung cancer	1483·9 (1203·0–1808·8)	3421·3 (2615·2–4328·9)	-1937·4 (-2889·4–-1155·4)	-56·0 (-67·5–-42·3)	1506·3 (1263·4–1787·3)	3793·6 (3092·0–4579·0)	-2287·4 (-3144·2–-1563·8)	-59·9 (-69·9–-47·8)
		Tuberculosis	322·5 (281·2–369·7)	805·2 (680·7–994·2)	-482·7 (-673·8–-360·2)	-59·7 (-67·7–-51·2)	1610·1 (1429·6–1793·5)	2910·8 (2120·7–3509·4)	-1300·7 (-1917·0–-535·2)	-44·0 (-54·6–-25·6)
	70+ years old	Age-related and other hearing loss	3373·6 (2412·4–4540·3)	3410·5 (2421·5–4615·9)	-36·9 (-160·3–76·3)	-1·0 (-4·2–2·7)	3069·9 (2134·8–4200·3)	3104·2 (2160·5–4323·3)	-34·3 (-156·3–71·6)	-1·0 (-4·4–2·6)
		Alzheimer's disease and other dementias	7651·8 (3704·4–16161·3)	4534·8 (2138·4–10372·6)	3117·0 (1546·9–5871·5)	71·4 (44·9–94·5)	5779·6 (2643·6–12798·5)	3506·2 (1606·4–8126·6)	2273·4 (1012·4–4857·5)	66·2 (46·3–86·0)
		Anxiety disorders	610·6 (413·2–859·4)	383·7 (259·3–540·9)	226·9 (138·2–338·7)	59·4 (41·3–76·2)	593·1 (410·6–839·4)	373·7 (257·6–518·6)	219·5 (129·6–333·8)	58·9 (41·0–74·0)
		COVID-19	1898·2 (1355·4–2983·4)	3252·8 (2282·1–5191·3)	-1354·7 (-2208·1–-923·8)	-41·5 (-42·6–-40·3)	NA	NA	NA	NA
		Chronic kidney disease	2350·0 (2018·0–2682·9)	2743·6 (2296·8–3198·4)	-393·5 (-845·7–52·1)	-13·9 (-28·2–2·1)	2364·7 (2057·6–2747·1)	2613·7 (2231·1–3183·1)	-249·0 (-815·9–236·6)	-8·9 (-26·5–10·0)
		Chronic obstructive pulmonary disease	9854·5 (7881·4–12147·7)	15785·7 (12811·9–18546·0)	-5931·2 (-9231·1–-2446·6)	-37·1 (-52·3–-18·0)	26715·5 (20902·9–30696·8)	34732·7 (30231·2–39947·6)	-8017·2 (-15298·0–-1743·3)	-22·7 (-41·2–-5·6)

		Cirrhosis and other chronic liver diseases	900·9 (746·6–1103·2)	1488·1 (1235·7–1760·5)	-587·2 (-914·1–-330·4)	-39·0 (-53·0–-25·0)	1963·0 (1538·8–2482·7)	2560·2 (2130·1–3250·5)	-597·2 (-1102·6–41·3)	-22·7 (-39·2–1·8)
		Depressive disorders	1032·0 (710·8–1395·5)	717·0 (491·6–983·0)	315·0 (215·7–430·9)	44·0 (39·2–49·0)	887·7 (612·0–1209·8)	618·4 (425·2–832·1)	269·3 (185·5–371·8)	43·6 (37·6–49·5)
		Diabetes mellitus	3801·1 (3209·3–4444·8)	3789·5 (3188·2–4569·3)	11·6 (-371·2–345·1)	0·5 (-9·4–10·0)	3227·5 (2802·5–3700·9)	2916·2 (2507·0–3386·1)	311·3 (-15·0–657·0)	10·9 (-0·5–24·5)
		Falls	2019·6 (1507·4–2487·7)	1938·4 (1449·5–2373·2)	81·3 (-169·8–337·0)	4·4 (-9·1–19·2)	1756·8 (1466·0–2084·6)	1625·5 (1349·8–2001·7)	131·3 (-271·9–345·1)	8·6 (-14·4–22·6)
		HIV/AIDS	22·8 (15·2–32·8)	95·5 (65·6–137·3)	-72·7 (-104·9–-48·4)	-76·1 (-79·3–-68·8)	2·7 (1·6–3·6)	12·0 (6·5–18·3)	-9·4 (-15·7–-4·4)	-76·8 (-87·9–-65·3)
		Headache disorders	430·6 (102·9–926·8)	286·4 (99·6–580·9)	144·3 (4·3–376·6)	46·4 (5·0–77·5)	421·3 (108·5–903·4)	287·3 (98·6–580·1)	134·0 (4·9–337·8)	43·3 (4·3–72·0)
		Ischemic heart disease	13922·3 (11369·0–16425·1)	18787·4 (15911·4–21891·4)	-4865·1 (-8407·5–-1684·4)	-25·5 (-41·0–-9·7)	12138·8 (10719·9–13851·6)	13478·5 (11771·4–15136·8)	-1339·6 (-3417·5–976·5)	-9·7 (-22·7–7·7)
		Low back pain	2215·5 (1543·2–2988·0)	1223·7 (832·0–1654·0)	991·8 (692·1–1347·6)	81·3 (73·6–89·2)	2677·2 (1827·3–3666·1)	1451·9 (966·6–2018·7)	1225·3 (860·0–1671·0)	84·8 (74·0–94·4)
		Lower respiratory infections	2003·1 (1588·4–2368·7)	2919·6 (2564·5–3284·3)	-916·5 (-1346·4–-476·7)	-31·2 (-44·4–-17·5)	3426·7 (2661·3–4051·5)	4297·3 (3730·8–4841·8)	-870·6 (-1748·1–-8·9)	-20·0 (-39·6–-0·2)
		Other musculoskeletal disorders	824·3 (544·8–1261·8)	658·8 (425·7–1052·1)	165·5 (107·8–244·7)	25·6 (17·3–35·1)	763·0 (492·2–1180·2)	615·2 (397·2–955·2)	147·7 (88·3–224·6)	24·3 (16·7–32·5)
		Road injuries	552·1 (469·6–637·3)	1209·6 (1042·2–1395·4)	-657·5 (-820·0–-517·5)	-54·2 (-61·8–-47·6)	721·8 (626·0–841·7)	1339·8 (1191·9–1521·7)	-618·0 (-760·7–-453·7)	-46·0 (-52·6–-36·2)
		Stroke	19719·8 (16368·8–22837·4)	28315·3 (24226·7–32847·4)	-8595·5 (-13896·9–-3791·1)	-30·0 (-43·0–-14·9)	31982·8 (27864·1–36933·3)	37995·2 (33246·7–42699·9)	-6012·4 (-12230·7–-39·2)	-15·6 (-29·7–-0·1)
		Tracheal, bronchus, and lung cancer	3068·9 (2484·6–3676·6)	7403·1 (5718·2–9146·3)	-4334·2 (-6151·4–-2689·8)	-58·0 (-68·1–-45·4)	2242·9 (1913·5–2664·3)	6021·3 (5028·6–7096·7)	-3778·4 (-4943·2–-2801·5)	-62·5 (-70·5–-53·4)
		Tuberculosis	618·8 (546·7–698·1)	1415·1 (1220·2–1750·4)	-796·4 (-1125·7–-607·0)	-56·0 (-64·6–-48·9)	2759·8 (2413·4–3122·1)	5496·0 (3990·2–6667·4)	-2736·1 (-4081·5–-1481·8)	-49·3 (-60·6–-35·5)
Sub-Saharan Africa	10-24 years old	Age-related and other hearing loss	167·3 (107·6–244·7)	168·9 (107·7–248·5)	-1·6 (-9·4–5·8)	-0·9 (-5·0–3·1)	162·0 (105·3–235·7)	163·5 (104·8–239·7)	-1·5 (-9·0–5·2)	-0·8 (-4·5–3·4)
		Alzheimer's disease and other dementias	0·0 (0·0–0·0)	0·0 (0·0–0·0)	0·0 (0·0–0·0)	NA (NA–NA)	0·0 (0·0–0·0)	0·0 (0·0–0·0)	0·0 (0·0–0·0)	NA (NA–NA)
		Anxiety disorders	629·8 (390·5–933·0)	439·2 (272·1–653·3)	190·6 (114·3–282·2)	43·6 (33·8–52·4)	536·8 (334·8–792·0)	370·8 (230·6–548·4)	165·9 (102·6–246·5)	44·9 (36·0–52·8)

		COVID-19	671·6 (499·2–1029·3)	674·3 (543·0–961·4)	-2·8 (-61·5–84·6)	-1·1 (-10·6–10·2)	NA	NA	NA	NA
		Chronic kidney disease	150·0 (125·8–179·9)	291·0 (235·8–357·6)	-141·0 (-198·8–-82·8)	-48·0 (-57·6–-35·8)	159·5 (133·8–184·2)	310·8 (245·7–357·2)	-151·3 (-196·8–-85·1)	-48·4 (-57·0–-35·0)
		Chronic obstructive pulmonary disease	36·2 (28·1–47·8)	36·6 (30·2–43·9)	-0·4 (-10·1–11·4)	-0·2 (-25·0–32·7)	39·2 (29·8–48·6)	43·2 (34·6–51·1)	-4·0 (-14·1–7·1)	-8·8 (-30·0–20·9)
		Cirrhosis and other chronic liver diseases	156·3 (113·2–202·7)	224·0 (171·3–276·3)	-67·7 (-102·6–-31·9)	-30·2 (-42·6–-14·6)	200·0 (162·8–247·1)	297·2 (243·8–368·2)	-97·2 (-162·4–-38·4)	-32·3 (-46·2–-13·9)
		Depressive disorders	682·3 (417·5–996·6)	475·9 (292·7–700·9)	206·4 (122·6–303·6)	43·5 (35·8–51·4)	610·4 (379·6–894·2)	414·2 (260·0–604·6)	196·2 (120·6–289·6)	47·4 (41·1–53·4)
		Diabetes mellitus	99·9 (80·2–120·6)	124·5 (103·0–150·5)	-24·5 (-38·2–-11·4)	-19·6 (-28·9–-9·7)	92·6 (74·4–108·8)	119·1 (102·0–136·8)	-26·5 (-41·8–-10·2)	-22·1 (-34·3–-9·3)
		Falls	80·8 (65·8–100·8)	184·6 (152·0–222·6)	-103·8 (-137·2–-78·6)	-56·0 (-64·8–-47·6)	90·6 (71·3–113·5)	212·9 (173·5–252·1)	-122·4 (-156·7–-89·6)	-57·4 (-64·9–-48·2)
		HIV/AIDS	1519·4 (1171·1–1973·0)	804·6 (619·1–1010·7)	714·9 (397·9–1124·2)	90·4 (47·5–150·6)	2082·8 (1443·7–3202·5)	401·5 (253·2–645·4)	1681·3 (1164·3–2591·9)	425·9 (335·3–530·3)
		Headache disorders	630·3 (85·5–1466·4)	432·3 (60·5–991·0)	198·0 (25·9–466·2)	45·9 (27·2–53·8)	619·2 (84·4–1439·1)	422·6 (60·1–962·8)	196·6 (25·1–464·1)	46·6 (28·5–55·1)
		Ischemic heart disease	53·8 (43·4–67·6)	63·4 (52·5–74·3)	-9·6 (-22·9–5·0)	-14·7 (-32·9–8·2)	56·0 (45·2–69·5)	55·4 (45·5–66·4)	0·6 (-13·9–14·8)	1·8 (-21·9–29·3)
		Low back pain	367·8 (237·9–521·6)	260·8 (167·0–367·9)	107·1 (69·3–153·8)	41·1 (34·5–48·2)	375·2 (246·1–530·0)	259·4 (168·1–361·0)	115·8 (75·3–169·5)	44·7 (37·6–51·5)
		Lower respiratory infections	412·0 (319·7–493·5)	414·9 (354·4–479·2)	-3·0 (-66·1–75·0)	-0·6 (-16·4–19·2)	719·9 (546·8–839·7)	741·1 (620·8–854·9)	-21·2 (-163·8–104·9)	-2·6 (-21·9–16·4)
		Other musculoskeletal disorders	110·3 (77·3–156·1)	62·0 (36·2–99·6)	48·3 (35·1–64·7)	81·8 (52·0–126·8)	93·5 (65·6–136·0)	50·2 (29·0–80·2)	43·3 (32·6–56·8)	90·3 (59·0–140·9)
		Road injuries	508·7 (414·3–625·0)	1327·5 (1124·9–1531·2)	-818·8 (-970·1–-678·0)	-61·7 (-66·1–-55·6)	712·6 (597·6–801·9)	1784·5 (1550·7–2041·6)	-1071·9 (-1279·6–-889·5)	-60·0 (-64·8–-54·2)
		Stroke	139·7 (117·8–164·8)	151·6 (130·6–174·1)	-12·0 (-32·7–12·1)	-7·7 (-20·3–8·4)	196·8 (163·1–233·6)	218·0 (190·1–246·2)	-21·2 (-53·1–15·6)	-9·6 (-23·8–7·5)
		Tracheal, bronchus, and lung cancer	2·4 (1·7–3·2)	1·8 (1·5–2·3)	0·6 (0·1–1·2)	32·3 (5·5–64·8)	1·9 (1·6–2·4)	2·2 (1·8–2·8)	-0·3 (-0·8–0·4)	-10·5 (-30·6–19·0)
		Tuberculosis	450·9 (375·3–548·7)	552·4 (413·5–698·1)	-101·5 (-235·0–55·3)	-17·4 (-35·7–15·3)	1106·1 (938·9–1336·2)	1288·3 (932·1–1681·0)	-182·3 (-561·2–214·6)	-12·8 (-33·7–22·0)
	25-49 years old	Age-related and other hearing loss	356·5 (235·7–505·3)	377·1 (246·9–539·9)	-20·6 (-40·8–-7·9)	-5·3 (-7·9–-2·8)	349·1 (231·1–494·0)	368·4 (241·4–524·6)	-19·3 (-39·3–-7·1)	-5·1 (-7·7–-2·5)
		Alzheimer's disease and	5·5 (2·8–11·5)	5·2 (2·4–11·3)	0·3 (-0·8–1·6)	8·7 (-13·5–44·7)	5·0 (2·6–10·1)	4·9 (2·4–10·2)	0·1 (-1·1–1·4)	4·6 (-18·7–39·2)

		other dementias								
		Anxiety disorders	664·1 (437·2–964·9)	522·6 (343·4–764·4)	141·6 (88·5–210·3)	27·3 (20·0–34·9)	591·2 (388·1–845·5)	457·2 (300·0–660·1)	134·0 (82·9–197·6)	29·5 (21·3–37·5)
		COVID-19	2885·5 (2480·5–3569·5)	5445·1 (4989·6–5998·3)	-2559·7 (-2835·1–-2183·6)	-47·1 (-51·7–-38·8)	NA	NA	NA	NA
		Chronic kidney disease	388·4 (336·2–462·7)	584·9 (477·2–725·5)	-196·6 (-320·8–-113·0)	-33·1 (-46·3–-22·0)	438·5 (380·4–501·3)	554·5 (459·2–679·4)	-116·0 (-233·0–-24·8)	-20·4 (-35·9–-4·8)
		Chronic obstructive pulmonary disease	161·6 (131·0–204·6)	216·8 (176·2–259·6)	-55·1 (-105·3–0·9)	-24·8 (-42·6–0·4)	191·1 (145·9–234·4)	253·1 (197·8–294·5)	-62·0 (-116·1–-1·3)	-23·8 (-41·6–-0·6)
		Cirrhosis and other chronic liver diseases	474·6 (384·5–567·3)	1259·7 (1040·2–1498·9)	-785·1 (-979·8–-619·5)	-62·2 (-67·5–-56·3)	660·6 (564·3–790·1)	1576·7 (1342·6–1953·6)	-916·1 (-1305·8–-666·2)	-57·8 (-66·7—46·7)
		Depressive disorders	1323·3 (880·4–1855·7)	961·9 (632·7–1376·6)	361·5 (238·9–507·4)	37·7 (32·5–43·5)	1272·4 (858·7–1787·7)	890·2 (585·7–1257·3)	382·2 (259·1–539·9)	43·0 (38·4–48·3)
		Diabetes mellitus	459·7 (381·6–561·1)	639·8 (553·1–762·5)	-180·2 (-233·0–-139·5)	-28·2 (-34·1–-23·0)	404·2 (347·7–468·7)	507·5 (445·2–580·6)	-103·2 (-166·6–-50·3)	-20·2 (-30·1–-10·5)
		Falls	111·4 (88·0–142·9)	313·4 (255·4–382·7)	-202·1 (-254·8–-157·7)	-64·4 (-70·8–-58·2)	124·7 (98·1–157·4)	337·4 (283·7–400·3)	-212·6 (-259·4–-169·5)	-63·0 (-69·0–-55·9)
		HIV/AIDS	6712·2 (5835·0–7886·7)	4987·4 (4311·9–5972·6)	1724·8 (918·8–2613·7)	34·9 (17·3–55·0)	6659·7 (4261·1–9935·3)	5200·2 (3215·6–8077·5)	1459·5 (791·7–2355·6)	28·6 (15·5–40·3)
		Headache disorders	873·4 (188·8–1829·9)	602·4 (135·2–1279·0)	271·0 (53·3–580·4)	45·0 (25·0–52·8)	862·2 (183·0–1805·4)	602·1 (134·0–1255·3)	260·1 (50·5–558·3)	43·1 (23·7–50·4)
		Ischemic heart disease	317·1 (265·6–389·2)	777·2 (665·4–904·1)	-460·2 (-576·9–-352·1)	-59·1 (-66·0–-50·3)	410·3 (342·0–497·8)	770·4 (668·0–872·3)	-360·1 (-486·5–-258·7)	-46·6 (-57·0–-35·8)
		Low back pain	1002·6 (681·7–1385·7)	700·0 (466·9–967·7)	302·6 (211·6–419·8)	43·3 (38·8–47·9)	1019·4 (695·1–1411·8)	709·6 (477·4–974·0)	309·8 (212·4–433·6)	43·8 (38·8–48·9)
		Lower respiratory infections	555·5 (441·7–651·0)	1038·9 (907·7–1190·7)	-483·4 (-591·9–-362·9)	-46·5 (-56·6–-38·4)	1008·2 (806·1–1154·9)	1432·8 (1257·0–1608·2)	-424·6 (-656·6–-214·4)	-29·5 (-43·9–-16·3)
		Other musculoskeletal disorders	510·0 (351·5–730·5)	373·6 (242·0–557·7)	136·4 (102·8–176·9)	37·3 (29·2–47·1)	457·4 (318·7–652·1)	338·9 (221·9–502·2)	118·5 (89·0–149·8)	35·7 (27·6–46·3)
		Road injuries	496·6 (421·5–574·0)	2158·6 (1900·1–2432·6)	-1661·9 (-1892·3–-1456·6)	-77·0 (-79·4–-74·6)	825·2 (723·4–913·8)	2593·3 (2356·8–2837·4)	-1768·2 (-1995·8–-1537·7)	-68·1 (-72·0–-64·2)
		Stroke	588·0 (506·5–695·2)	941·8 (818·7–1089·0)	-353·8 (-469·7–-253·3)	-37·5 (-44·9–-28·9)	963·2 (823·2–1183·4)	1279·4 (1119·9–1424·6)	-316·2 (-503·0–-81·2)	-24·5 (-37·2–-6·6)
		Tracheal, bronchus,	31·5 (25·1–39·0)	82·0 (69·7–98·1)	-50·5 (-63·3–-41·1)	-61·5 (-67·3–-55·8)	33·2 (28·1–40·2)	106·3 (89·7–128·3)	-73·1 (-94·4–-53·5)	-68·5 (-74·5–-58·4)

		and lung cancer								
		Tuberculosis	1144·9 (974·0–1363·6)	2488·0 (1883·5–3074·7)	-1343·2 (-1848·9–-673·7)	-53·5 (-62·1–-36·4)	3161·0 (2738·1–3718·4)	5296·5 (3979·3–6536·5)	-2135·5 (-3300·9–-817·9)	-39·6 (-51·6–-18·9)
	50-69 years old	Age-related and other hearing loss	1141·6 (768·5–1661·5)	1202·2 (801·0–1749·8)	-60·6 (-112·0–-22·1)	-5·0 (-7·2–-2·5)	1163·0 (784·9–1682·2)	1240·9 (823·7–1811·1)	-77·9 (-137·0–-34·2)	-6·2 (-8·4–-3·8)
		Alzheimer's disease and other dementias	474·4 (209·4–1147·8)	333·3 (144·2–810·6)	141·1 (61·2–327·1)	43·1 (32·2–55·1)	479·8 (217·6–1125·7)	339·2 (149·7–823·1)	140·6 (58·5–316·8)	42·4 (27·7–57·4)
		Anxiety disorders	541·0 (360·8–793·1)	380·8 (245·7–549·2)	160·2 (98·6–241·0)	42·5 (27·7–55·3)	498·2 (335·4–728·7)	346·9 (227·8–506·6)	151·3 (94·9–227·1)	44·0 (30·3–57·9)
		COVID-19	9776·4 (8975·1–10747·7)	20793·5 (19209·1–22403·2)	-11017·1 (-11820·6–-10097·2)	-53·0 (-54·2–-50·7)	NA	NA	NA	NA
		Chronic kidney disease	1664·5 (1456·7–1916·7)	2361·0 (2008·4–2797·8)	-696·5 (-1062·4–-377·5)	-29·1 (-40·6–-16·3)	1780·4 (1572·2–2048·4)	2511·5 (2140·1–2973·3)	-731·1 (-1191·6–-286·8)	-28·6 (-41·8–-12·6)
		Chronic obstructive pulmonary disease	1326·2 (1081·6–1659·9)	1918·9 (1608·7–2222·9)	-592·7 (-963·4–-111·6)	-30·4 (-45·8–-7·0)	1626·3 (1135·8–2019·0)	2539·1 (1931·9–2892·4)	-912·8 (-1389·9–-193·2)	-35·4 (-52·9–-10·3)
		Cirrhosis and other chronic liver diseases	1567·8 (1358·5–1827·3)	3836·7 (3225·7–4482·2)	-2268·9 (-2834·1–-1733·0)	-59·0 (-64·2–-52·1)	2296·0 (1956·3–2654·4)	5499·7 (4622·8–6887·9)	-3203·7 (-4491·3–-2326·4)	-57·9 (-66·5–-48·1)
		Depressive disorders	1689·7 (1122·4–2296·0)	1253·6 (823·0–1700·3)	436·1 (291·1–601·0)	34·9 (30·9–39·1)	1690·0 (1120·9–2315·6)	1200·5 (796·0–1665·0)	489·5 (316·6–689·5)	40·8 (36·5–45·1)
		Diabetes mellitus	3381·1 (2948·4–3887·8)	4214·9 (3703·8–4893·1)	-833·8 (-1169·6–-506·8)	-19·7 (-26·5–-12·6)	2932·9 (2601·4–3320·8)	3668·4 (3242·2–4171·9)	-735·5 (-1211·9–-321·7)	-19·8 (-30·4–-9·2)
		Falls	412·1 (348·3–488·2)	832·6 (679·0–1020·4)	-420·5 (-595·1–-296·2)	-50·2 (-60·3–-40·8)	481·0 (398·6–574·0)	998·1 (839·9–1196·0)	-517·1 (-697·4–-355·9)	-51·5 (-61·6–-40·3)
		HIV/AIDS	4591·0 (3489·4–6173·0)	4773·4 (3694·6–6363·3)	-182·4 (-1905·7–1470·6)	-2·2 (-33·4–35·3)	2885·4 (1909·6–3970·9)	2204·8 (1387·5–3176·5)	680·6 (204·9–1277·2)	32·5 (8·3–65·3)
		Headache disorders	753·0 (245·9–1482·0)	510·7 (174·4–989·3)	242·4 (67·9–488·8)	47·1 (32·4–57·8)	737·8 (245·6–1455·7)	509·3 (174·5–986·9)	228·6 (67·5–474·3)	44·6 (29·5–54·0)
		Ischemic heart disease	3368·4 (2858·4–4050·5)	5873·8 (5094·2–6740·3)	-2505·4 (-3282·6–-1721·7)	-42·5 (-51·3–-30·1)	4068·8 (3386·2–4860·6)	6120·8 (5308·3–7051·2)	-2051·9 (-3102·2–-1122·7)	-33·3 (-46·1–-20·1)
		Low back pain	2052·4 (1405·9–2882·3)	1423·7 (969·9–1990·8)	628·6 (424·7–862·7)	44·3 (38·7–49·3)	2172·6 (1493·5–3047·0)	1478·9 (1002·1–2065·7)	693·8 (474·9–955·8)	47·1 (42·1–52·0)

		Lower respiratory infections	2350·7 (1896·2–2776·4)	3796·2 (3335·2–4300·3)	-1445·4 (-1914·4–-1040·9)	-38·0 (-49·7–-29·1)	3553·8 (2832·5–4187·3)	5450·5 (4705·9–6230·3)	-1896·6 (-2761·2–-1093·4)	-34·6 (-46·9–-21·2)
		Other musculoskeletal disorders	836·1 (558·6–1195·8)	612·6 (391·8–891·5)	223·6 (163·8–299·0)	37·1 (30·7–45·9)	741·2 (497·3–1057·1)	541·7 (345·0–802·9)	199·5 (141·7–267·9)	37·4 (30·6–47·1)
		Road injuries	915·3 (799·9–1057·4)	2302·5 (2039·3–2603·2)	-1387·2 (-1619·8–-1174·2)	-60·2 (-64·3–-55·7)	1242·0 (1089·4–1385·8)	2835·9 (2548·5–3173·1)	-1593·9 (-1896·4–-1304·5)	-56·1 (-61·7–-50·5)
		Stroke	5461·3 (4687·0–6335·2)	7591·3 (6660·4–8659·7)	-2130·0 (-2932·7–-1247·7)	-27·9 (-36·8–-17·3)	8314·2 (7079·8–9756·2)	10794·4 (9470·1–11994·2)	-2480·3 (-4166·3–-769·5)	-22·8 (-36·1–-7·5)
		Tracheal, bronchus, and lung cancer	341·3 (284·6–395·0)	970·3 (843·4–1131·2)	-629·1 (-752·4–-520·3)	-64·8 (-69·1–-60·3)	301·2 (249·3–355·2)	1067·6 (905·2–1298·0)	-766·4 (-977·7–-585·2)	-71·6 (-77·0–-63·5)
		Tuberculosis	3090·9 (2632·2–3620·8)	5906·1 (4486·0–7314·7)	-2815·2 (-4101·2–-1226·1)	-47·1 (-57·1–-27·8)	7949·5 (6786·7–9471·5)	13427·7 (9824·9–16778·7)	-5478·2 (-8544·4–-1561·6)	-40·0 (-53·3–-16·3)
	70+ years old	Age-related and other hearing loss	2814·9 (2014·1–3834·2)	2899·3 (2060·4–3973·4)	-84·4 (-200·5–-7·8)	-2·9 (-5·9–-0·3)	2707·8 (1922·8–3714·7)	2983·4 (2118·1–4093·0)	-275·6 (-429·5–-155·4)	-9·2 (-12·3–-6·3)
		Alzheimer's disease and other dementias	5470·2 (2451·4–12482·1)	2972·7 (1316·7–7237·0)	2497·6 (1106·1–5321·4)	85·5 (71·3–97·7)	4809·9 (2216·2–10831·5)	2724·6 (1256·0–6266·0)	2085·3 (952·6–4455·1)	78·0 (61·2–94·2)
		Anxiety disorders	451·5 (301·1–660·0)	255·3 (169·1–365·8)	196·2 (116·8–304·3)	77·2 (54·1–97·6)	420·1 (281·4–606·8)	247·0 (164·5–356·8)	173·1 (104·5–261·2)	70·5 (49·2–90·2)
		COVID-19	25588·8 (23706·5–27417·0)	45397·7 (41991·3–48652·9)	-19809·0 (-21212·3–-18198·3)	-43·6 (-44·1–-42·8)	NA	NA	NA	NA
		Chronic kidney disease	4804·5 (4194·3–5501·6)	6930·7 (6180·5–7816·3)	-2126·2 (-2836·5–-1286·8)	-30·4 (-39·5–-20·0)	3965·1 (3438·5–4555·5)	6603·4 (5530·0–8045·1)	-2638·3 (-4039·5–-1530·1)	-39·5 (-51·7–-26·0)
		Chronic obstructive pulmonary disease	4642·2 (3904·0–6021·0)	6867·2 (5935·3–7760·1)	-2224·9 (-3406·2–-672·1)	-32·0 (-45·8–-11·1)	5016·5 (3723·4–6201·6)	8597·0 (6490·2–9784·6)	-3580·5 (-4992·2–-846·0)	-41·1 (-54·6–-13·5)
		Cirrhosis and other chronic liver diseases	2644·0 (2320·9–3051·6)	3562·5 (3118·5–4019·0)	-918·5 (-1350·9–-445·0)	-25·6 (-35·4–-13·7)	3562·1 (2826·5–4423·2)	4938·1 (4192·2–6096·0)	-1376·0 (-2404·1–-252·5)	-27·4 (-44·7–-5·5)
		Depressive disorders	1939·9 (1296·2–2691·7)	1533·1 (1038·8–2130·9)	406·8 (263·6–561·6)	26·6 (23·0–30·1)	1945·5 (1307·5–2688·0)	1492·7 (1000·7–2050·1)	452·8 (301·6–637·4)	30·4 (27·0–34·3)
		Diabetes mellitus	8091·3 (7256·2–9018·7)	9161·8 (8142·7–10387·7)	-1070·6 (-1874·4–-353·2)	-11·6 (-19·2–-4·3)	6014·9 (5296·9–6653·4)	7248·4 (6505·7–8209·7)	-1233·5 (-2151·8–-415·2)	-16·8 (-27·3–-6·1)

		Falls	1990·3 (1708·8–2407·2)	2143·7 (1852·0–2573·4)	-153·4 (-587·9–205·8)	-6·8 (-24·8–10·2)	1839·8 (1552·4–2237·1)	2288·7 (1955·6–2767·4)	-448·9 (-911·4–54·1)	-19·1 (-35·3–2·4)
		HIV/AIDS	1128·9 (891·7–1408·2)	1105·4 (887·7–1388·4)	23·4 (-285·2–339·4)	3·1 (-22·7–33·2)	634·6 (425·9–867·3)	825·2 (526·2–1188·6)	-190·6 (-399·4–-12·1)	-22·1 (-37·4–-1·7)
		Headache disorders	413·9 (157·0–814·2)	307·9 (117·0–613·7)	106·0 (33·1–217·5)	34·4 (16·7–43·9)	419·9 (160·1–832·4)	308·9 (118·1–605·5)	111·0 (33·1–234·8)	35·8 (17·0–47·0)
		Ischemic heart disease	12708·2 (11156·3–14770·0)	15280·9 (13587·3–17092·9)	-2572·7 (-4815·7–-66·4)	-16·6 (-29·3–-0·5)	13275·0 (11319·4–15454·6)	13557·4 (11832·7–15372·7)	-282·4 (-3101·8–2429·3)	-1·7 (-21·3–19·4)
		Low back pain	2557·1 (1765·1–3480·8)	1934·0 (1317·8–2637·8)	623·0 (434·0–841·6)	32·4 (28·2–37·4)	2680·8 (1842·2–3653·5)	2009·2 (1370·2–2727·7)	671·6 (473·4–921·9)	33·6 (28·6–38·7)
		Lower respiratory infections	8420·6 (6923·0–9822·1)	10829·1 (9851·5–11880·9)	-2408·5 (-3920·3–-1049·4)	-22·2 (-34·3–-9·9)	10444·3 (8625·2–12698·5)	14233·1 (12364·2–16112·3)	-3788·7 (-6043·6–-1173·1)	-26·5 (-40·1–-8·1)
		Other musculoskeletal disorders	943·5 (656·7–1358·5)	717·4 (473·8–1085·2)	226·1 (157·9–304·0)	32·3 (23·2–43·8)	844·4 (578·1–1228·3)	632·9 (417·7–950·6)	211·5 (146·8–295·2)	34·2 (24·0–47·9)
		Road injuries	1105·2 (985·1–1252·5)	2307·8 (2086·1–2544·3)	-1202·6 (-1396·3–-1023·6)	-52·1 (-56·8–-47·1)	1275·6 (1116·2–1425·4)	2710·0 (2437·8–2974·0)	-1434·3 (-1691·2–-1152·2)	-52·8 (-58·6–-46·4)
		Stroke	19897·4 (17536·9–22470·7)	20144·9 (18019·7–22395·5)	-247·6 (-2718·1–2555·0)	-1·0 (-12·9–13·7)	24153·3 (20983·9–27182·0)	25328·1 (22587·5–27976·2)	-1174·8 (-5289·3–2769·0)	-4·4 (-19·1–11·5)
		Tracheal, bronchus, and lung cancer	555·7 (469·3–632·8)	1422·7 (1290·6–1594·6)	-867·0 (-1008·5–-744·5)	-60·9 (-66·0–-55·9)	395·7 (320·9–475·5)	1398·1 (1184·5–1646·1)	-1002·4 (-1214·5–-780·3)	-71·6 (-77·1–-64·2)
		Tuberculosis	5693·4 (5036·2–6555·1)	8904·7 (7142·1–10801·6)	-3211·2 (-4961·8–-1219·2)	-35·5 (-47·9–-16·6)	11283·0 (9602·1–14530·0)	18362·2 (13319·3–23554·5)	-7079·2 (-12187·8–-2201·5)	-37·6 (-53·6–-16·3)

The health outcomes presented here reflect the top 20 causes of disease burden, measured by Disability-Adjusted Life Years (DALYs), observed across females and males for the age group of 10 years and older globally in 2021. Health conditions are ordered alphabetically for each location and age group. The absolute differences between females and males were calculated as the DALY rate for females minus the rate for males for each specific cause and year, with a positive value indicating a higher rate for females compared to males. The relative gap was computed as a relative percent difference, with a positive value indicating higher values among females relative to males. Cell colours denote whether the absolute and relative gaps in DALY rates indicate that the cause disproportionately affects females (red) or males (blue) in the super-region and age group.

DALYs = Disability-Adjusted Life Years. 95% UI = 95% uncertainty interval. COVID = Coronavirus disease. HIV = Human Immunodeficiency Virus. AIDS = Acquired Immunodeficiency Syndrome.

Section S3. Supplementary Figures

The following pages include a number of supplementary figures presenting different components of our results.

Figure S1. Global and regional relative gaps in Disability−Adjusted Life Year (DALY) rates (per 100,000 population) between females and males, age−standardised (10 years and older), 2021

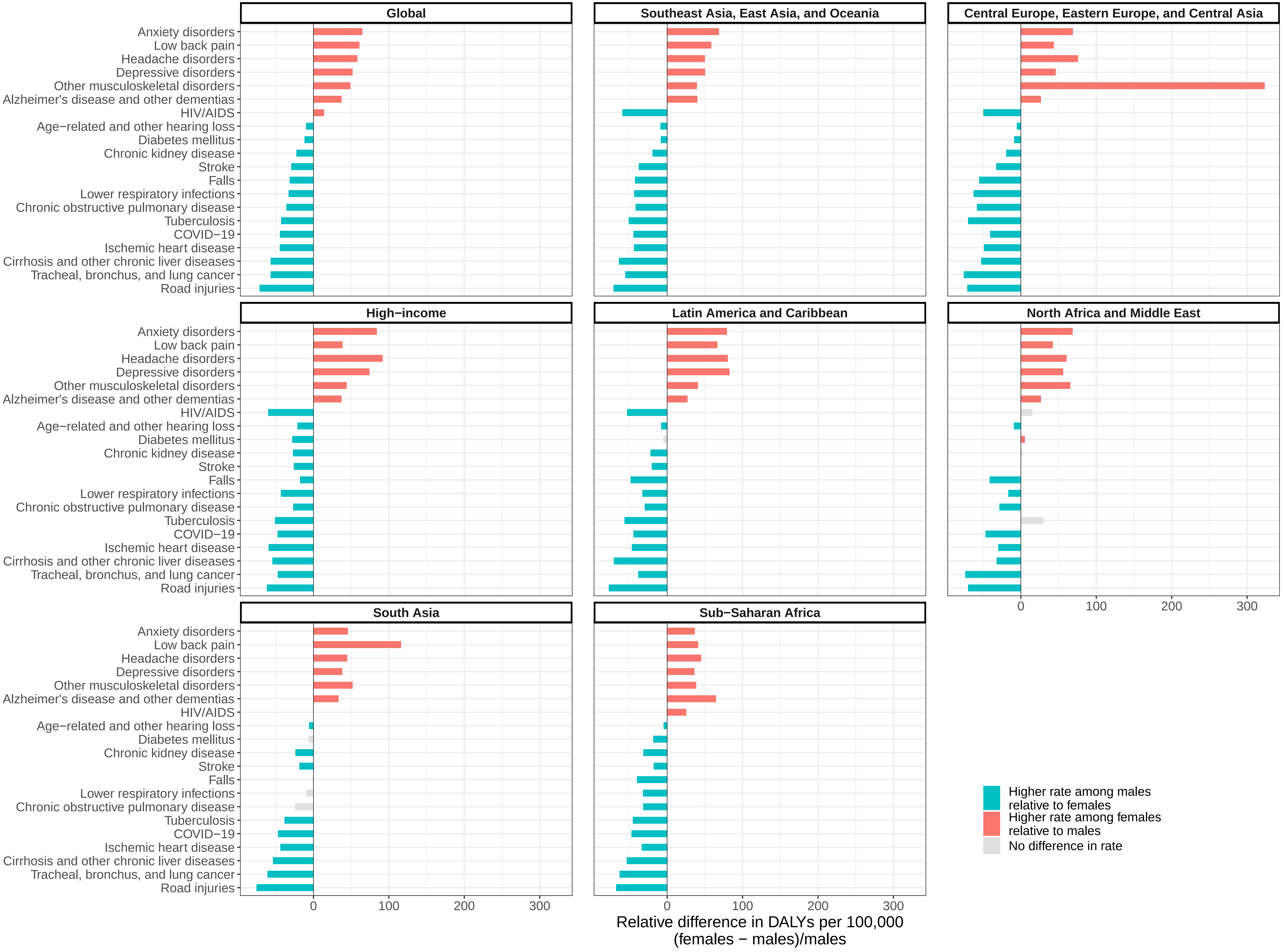

Figure S2. Global and regional absolute difference in Disability−Adjusted Life Year (DALY) rates (per 100,000 population) between females and males, 10−24 years old, 2021

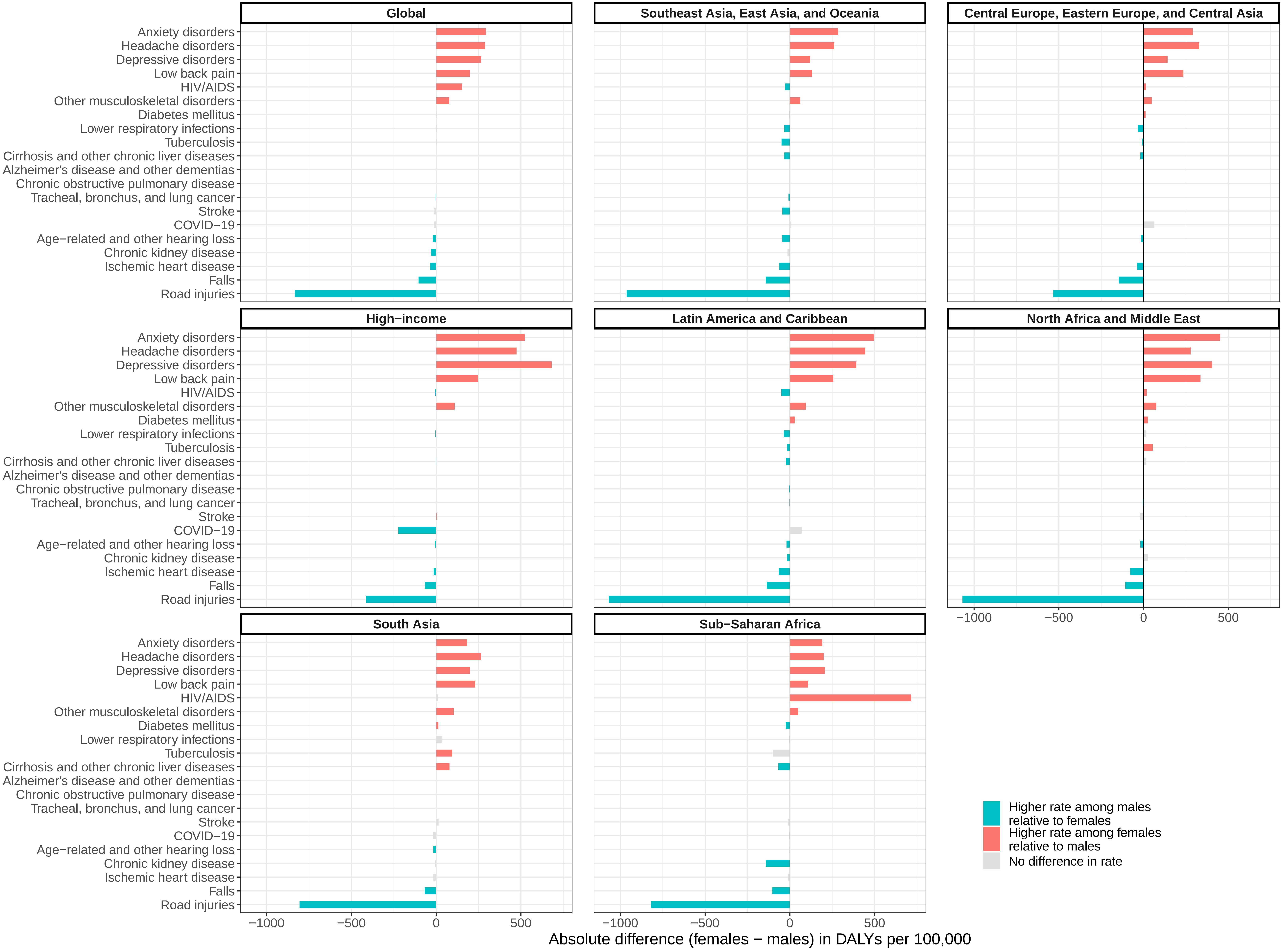

Figure S3. Global and regional absolute difference in Disability−Adjusted Life Year (DALY) rates (per 100,000 population) between females and males, 25−49 years old, 2021

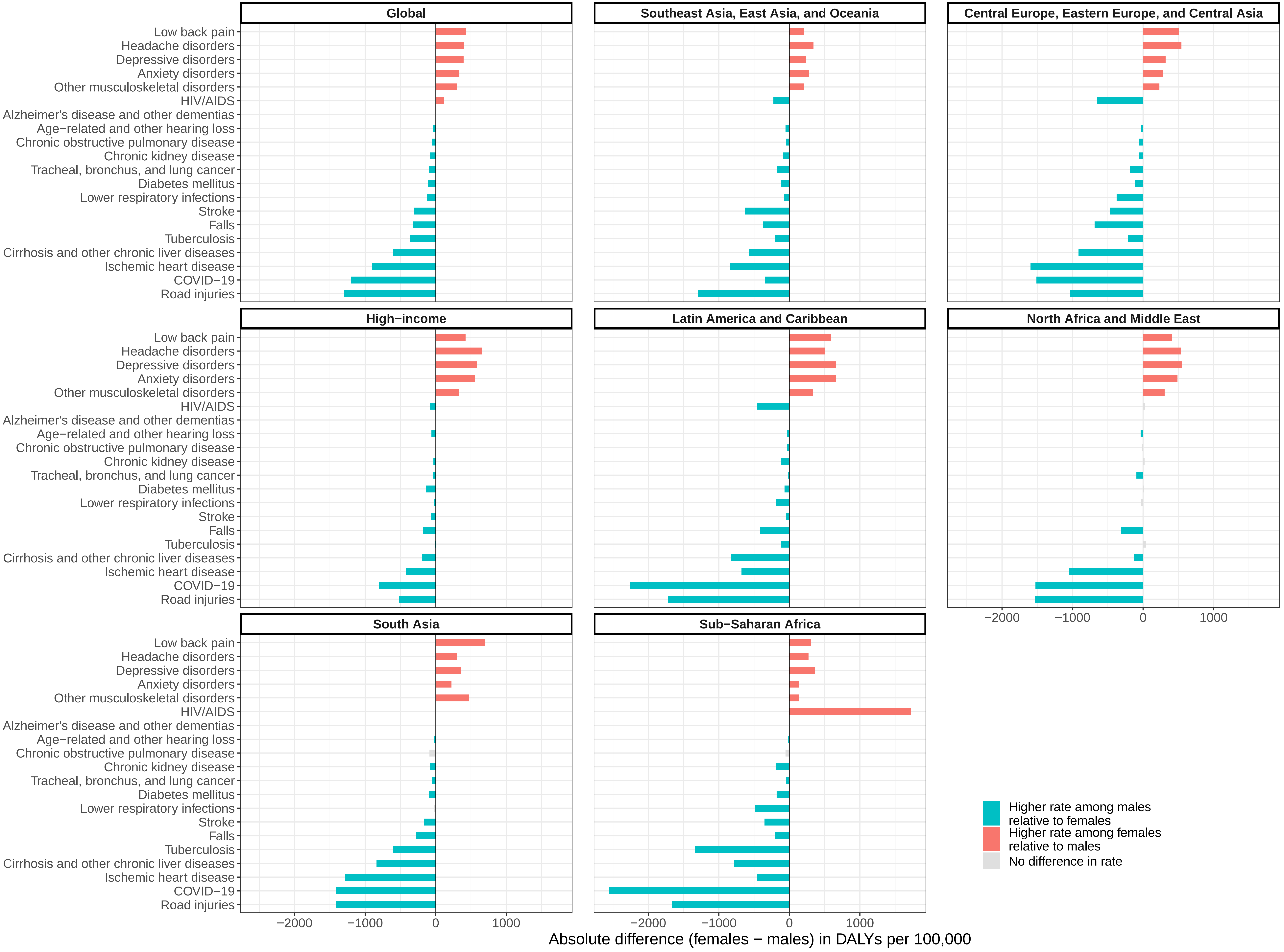

Figure S4. Global and regional absolute difference in Disability−Adjusted Life Year (DALY) rates (per 100,000 population) between females and males, 50−69 years old, 2021

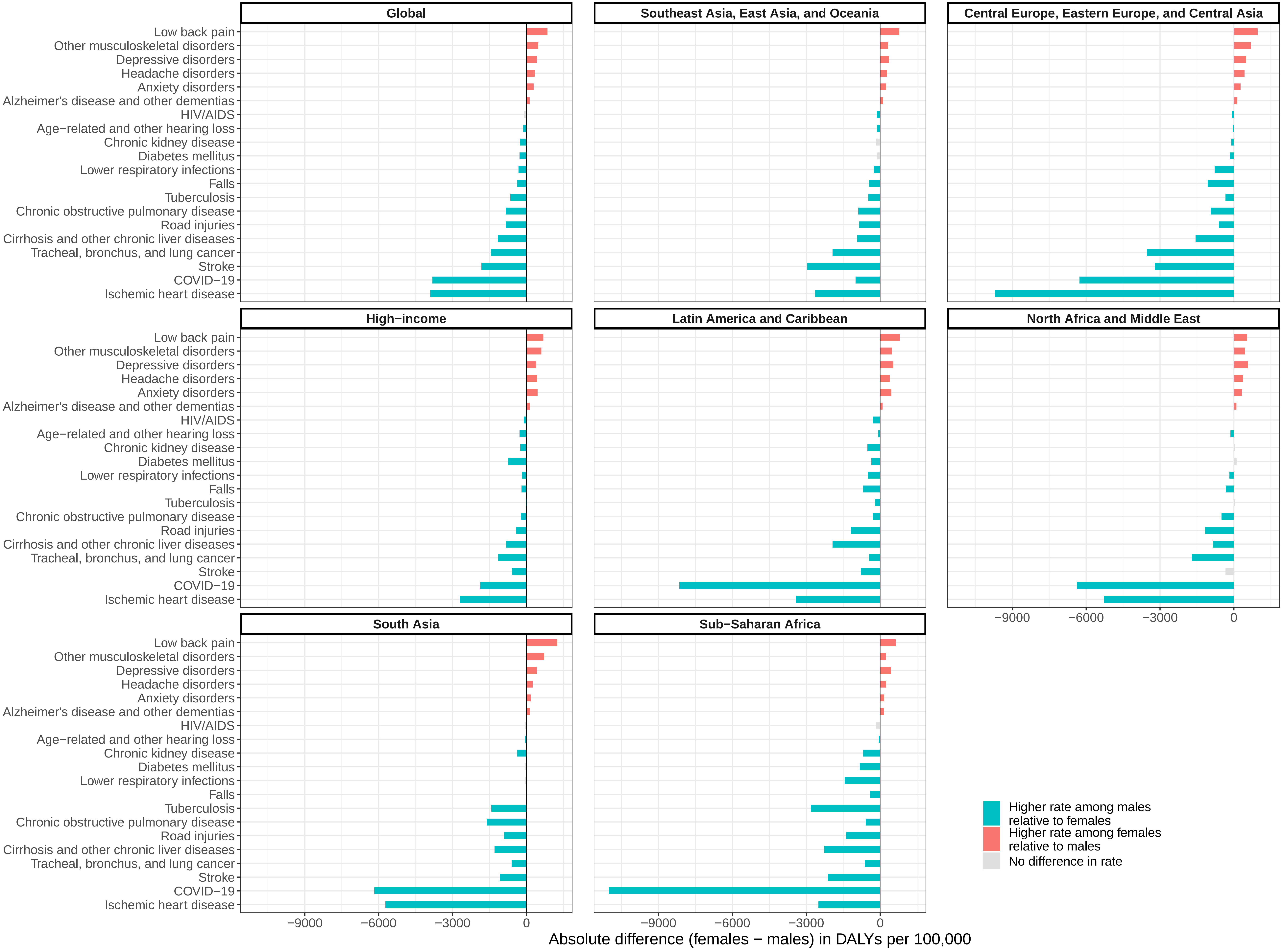

Figure S5. Global and regional absolute difference in Disability−Adjusted Life Year (DALY) rates (per 100,000 population) between females and males, 70+ years old, 2021

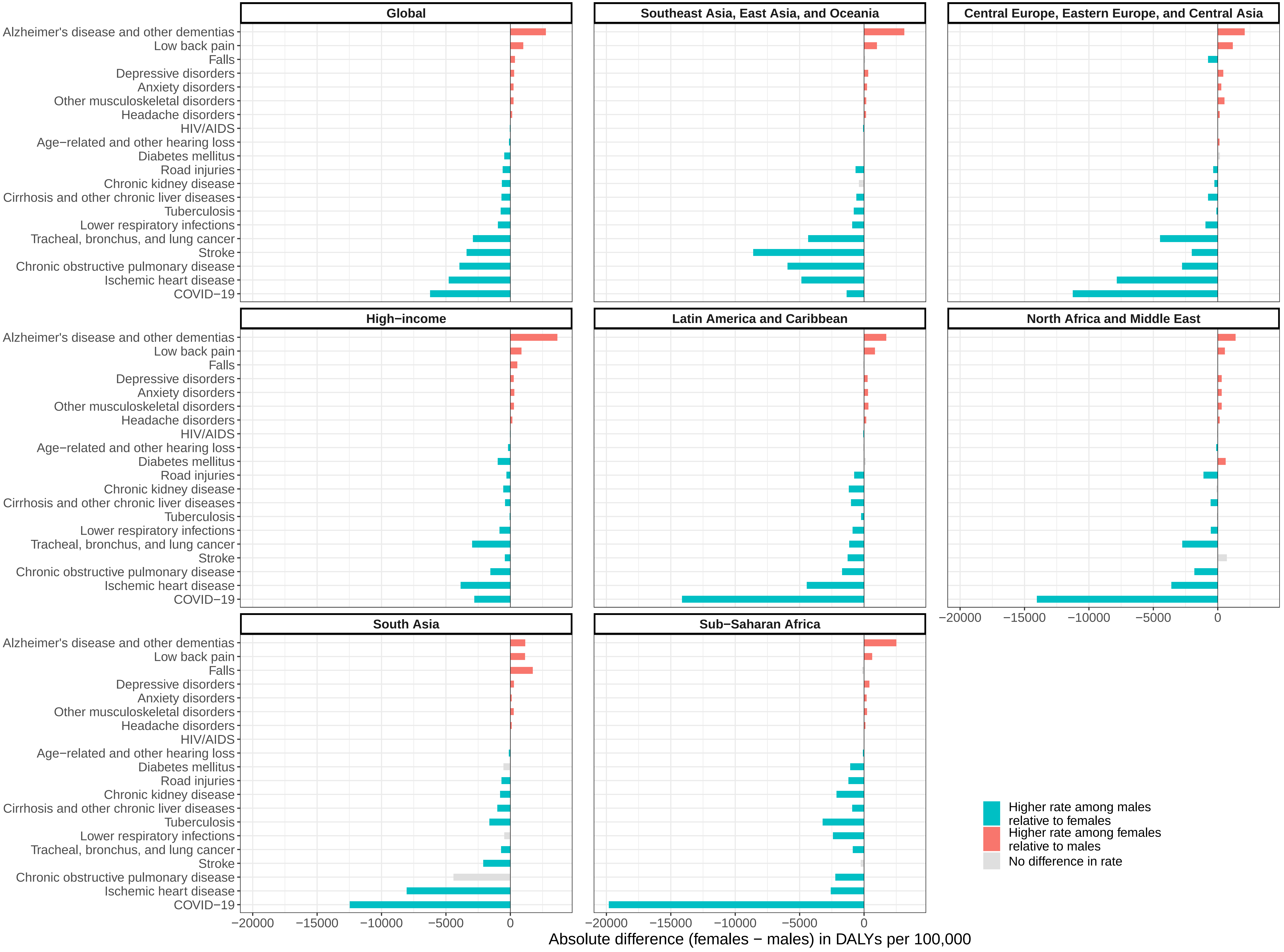

Figure S6. Global and regional relative gaps in Disability−Adjusted Life Year (DALY) rates (per 100,000 population) between females and males, 10−24 years old, 2021

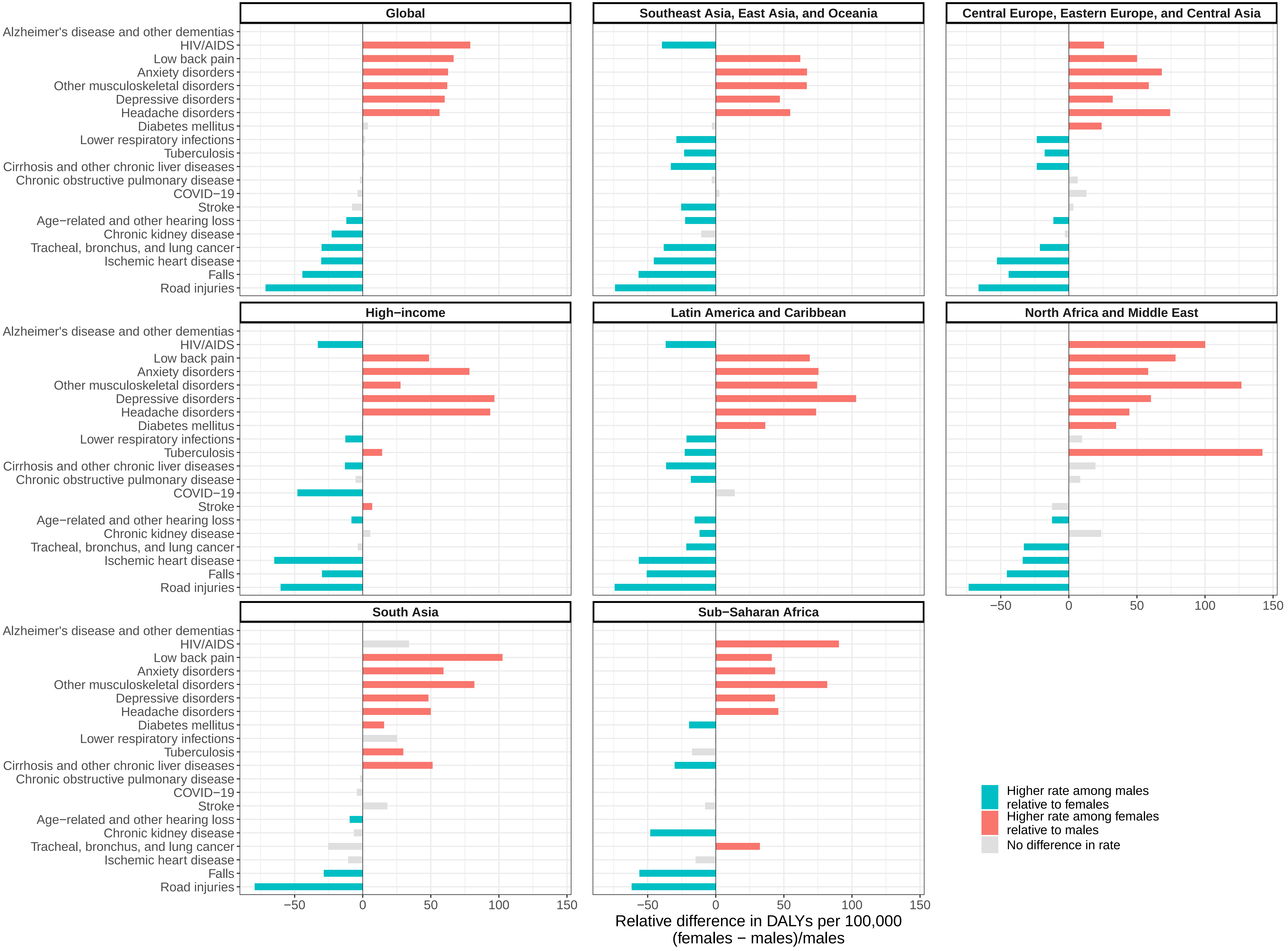

Figure S7. Global and regional relative gaps in Disability−Adjusted Life Year (DALY) rates (per 100,000 population) between females and males, 25−49 years old, 2021

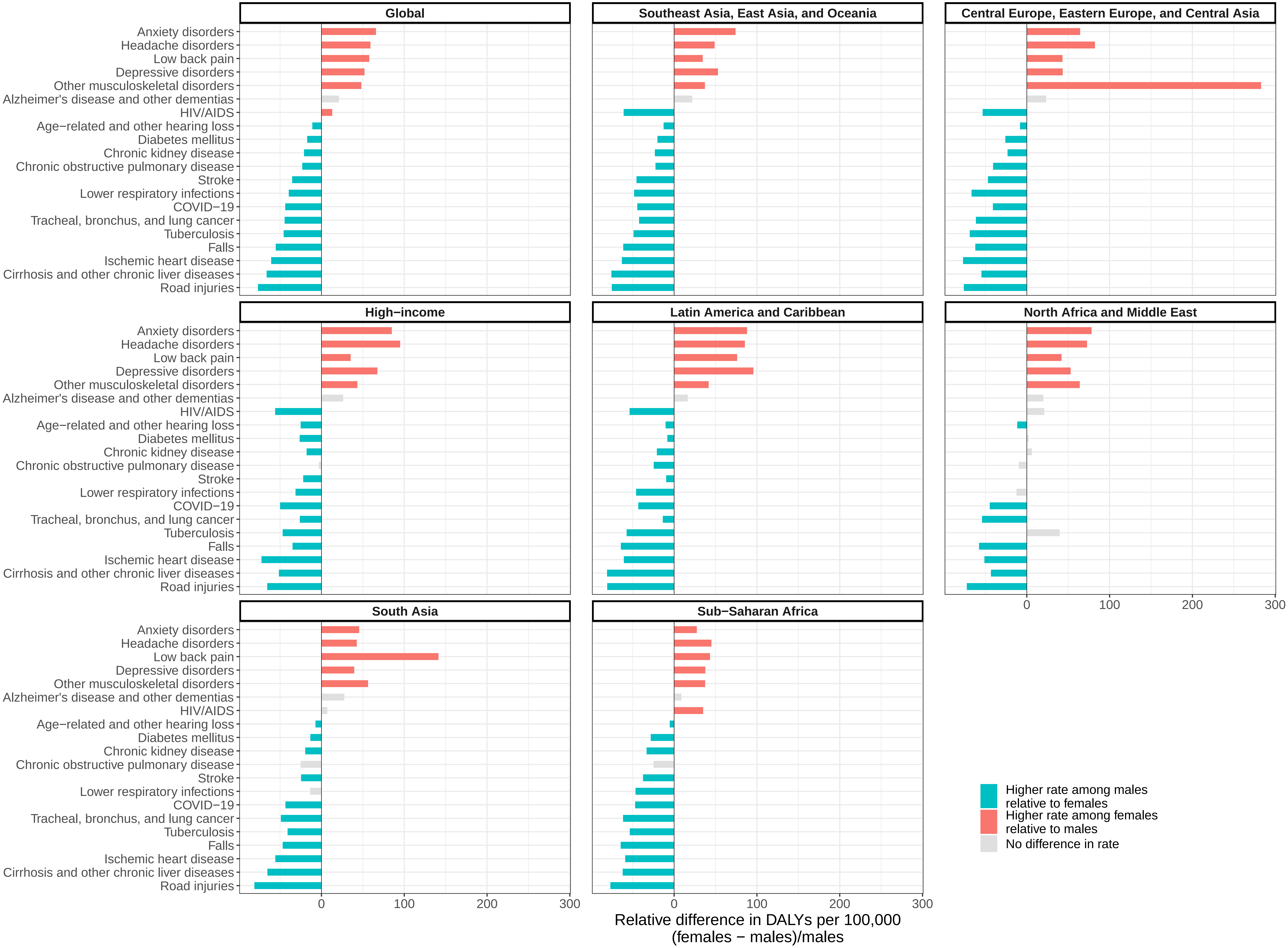

Figure S8. Global and regional relative gaps in Disability−Adjusted Life Year (DALY) rates (per 100,000 population) between females and males, 50−69 years old, 2021

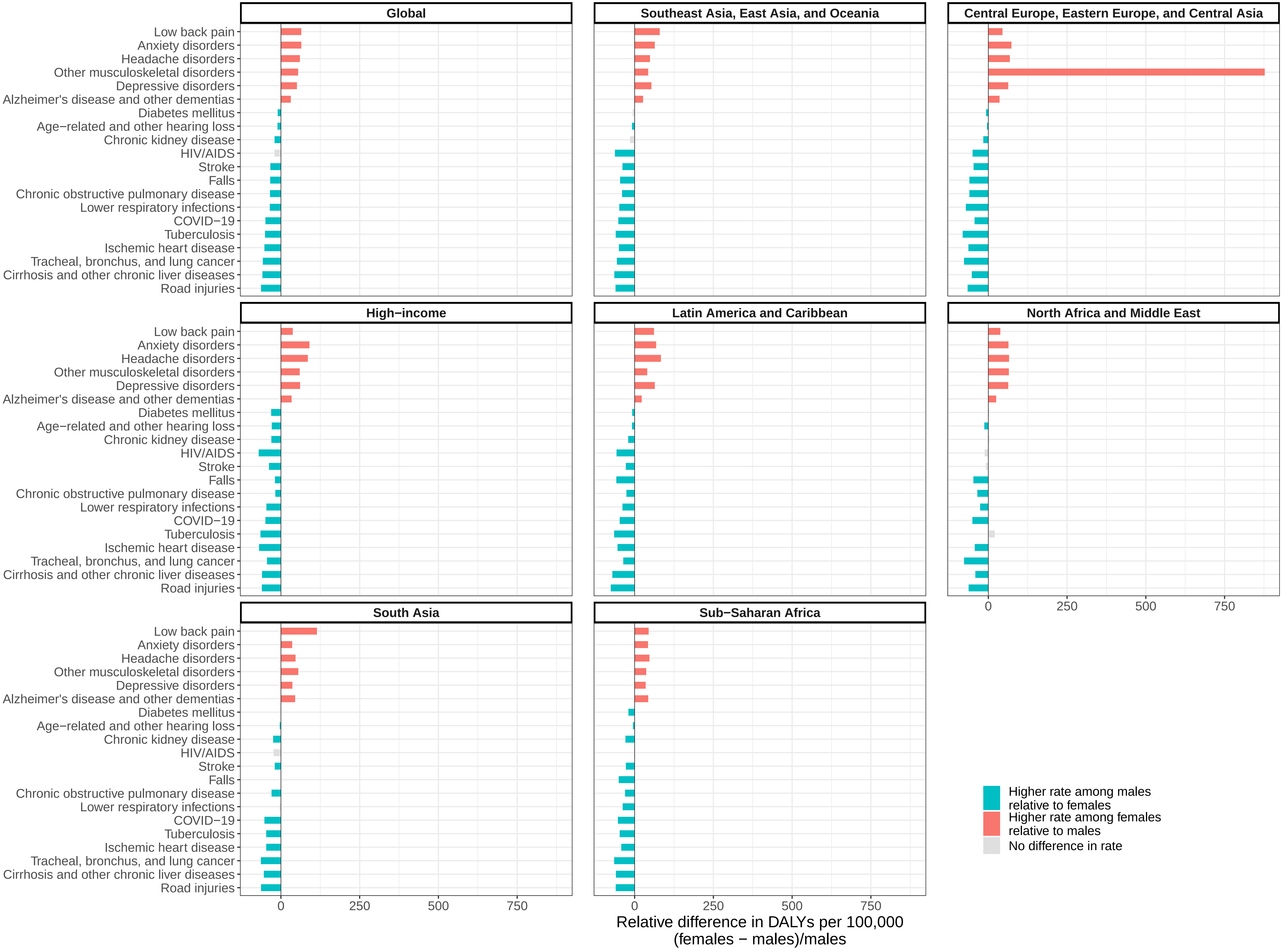

Figure S9. Global and regional relative gaps in Disability−Adjusted Life Year (DALY) rates (per 100,000 population) between females and males, 70+ years old, 2021

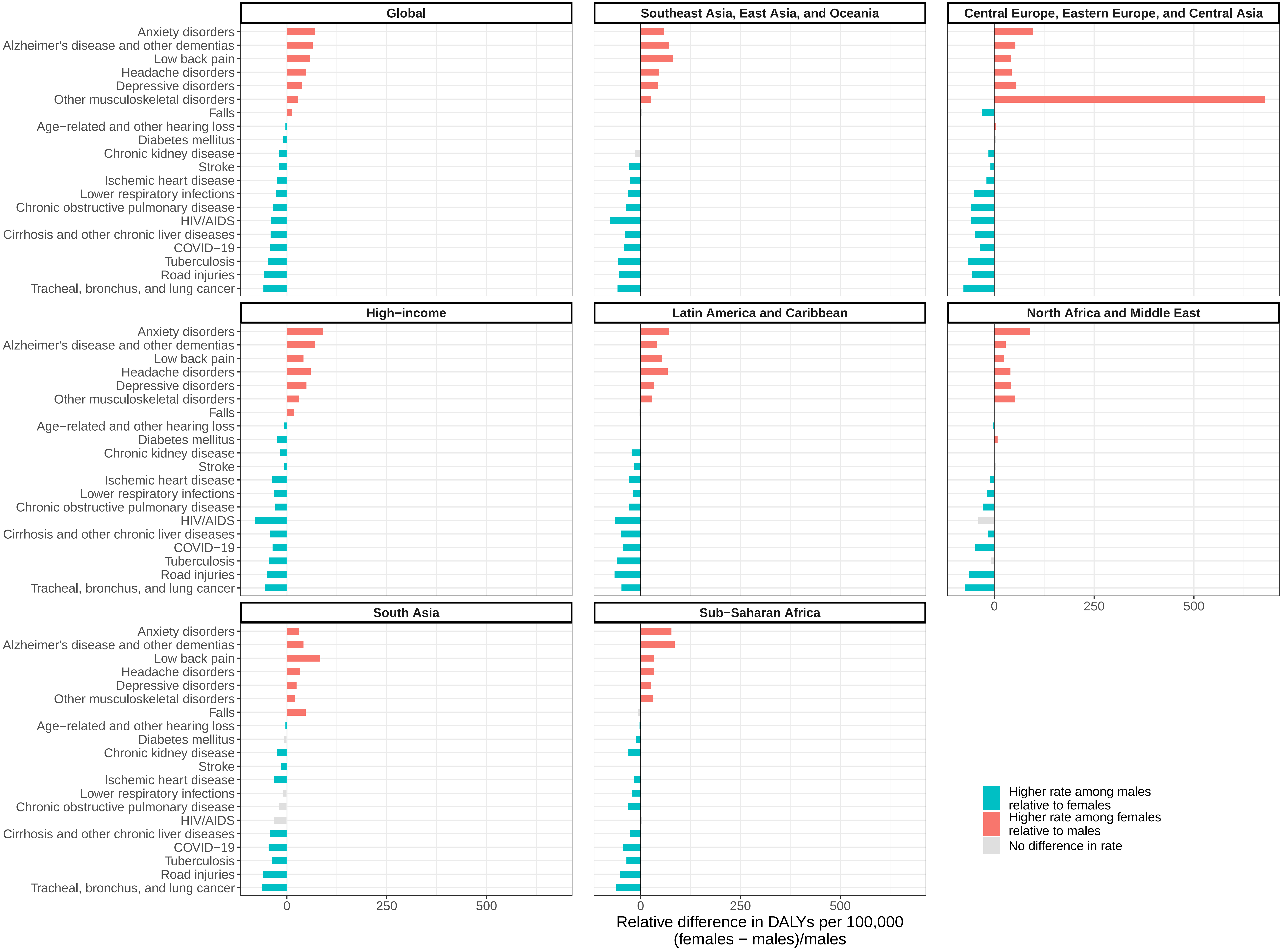

Figure S10. Change in global absolute difference in Disability−Adjusted Life Year (DALY) rates (per 100,000) among females and males between 1990 and 2021, age−standardised (10 years and older)

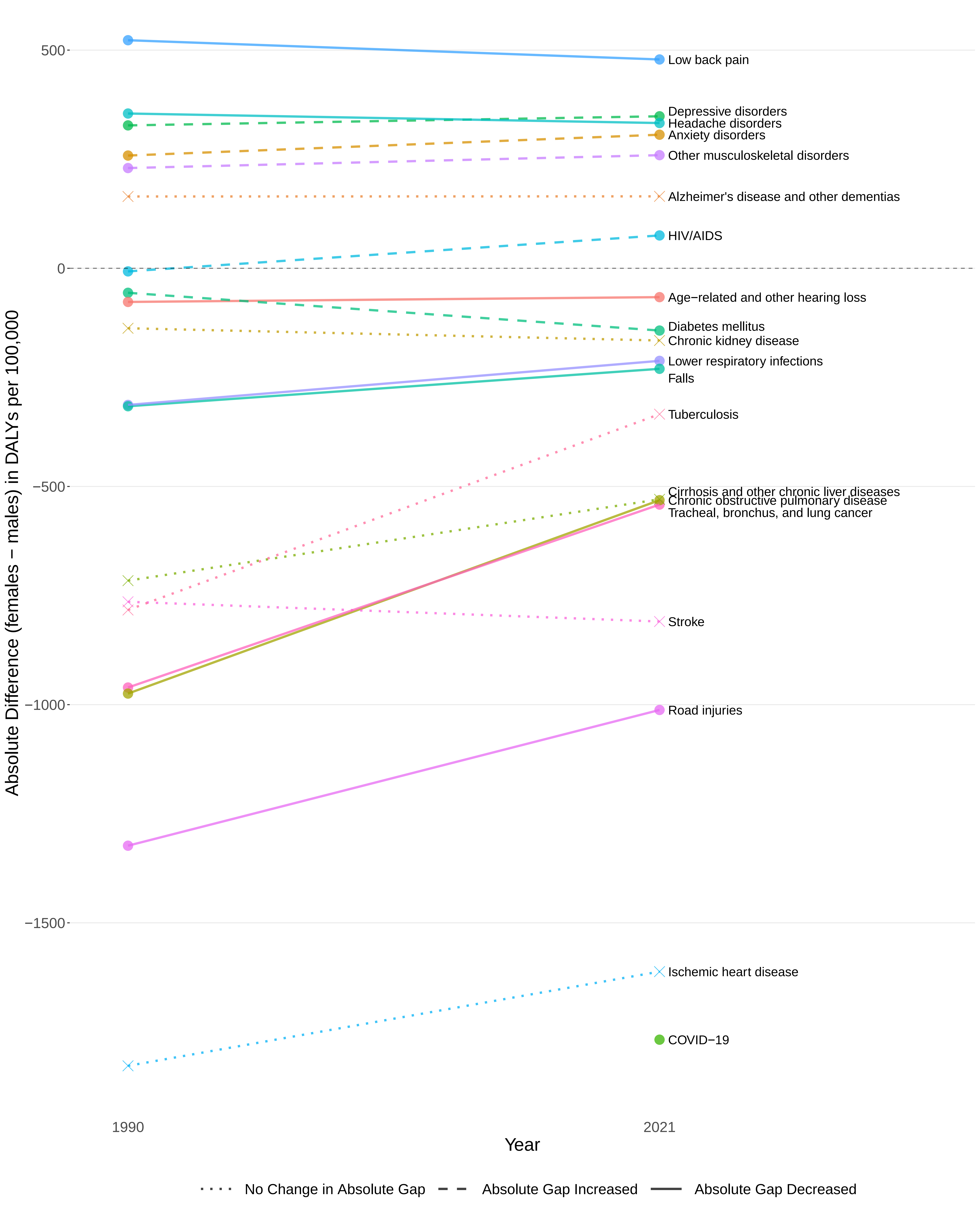

Figure S11. Change in global relative gaps in Disability−Adjusted Life Year (DALY) rates (per 100,000 population) among females and males between 1990 and 2021, age−standardised (10 years and older)

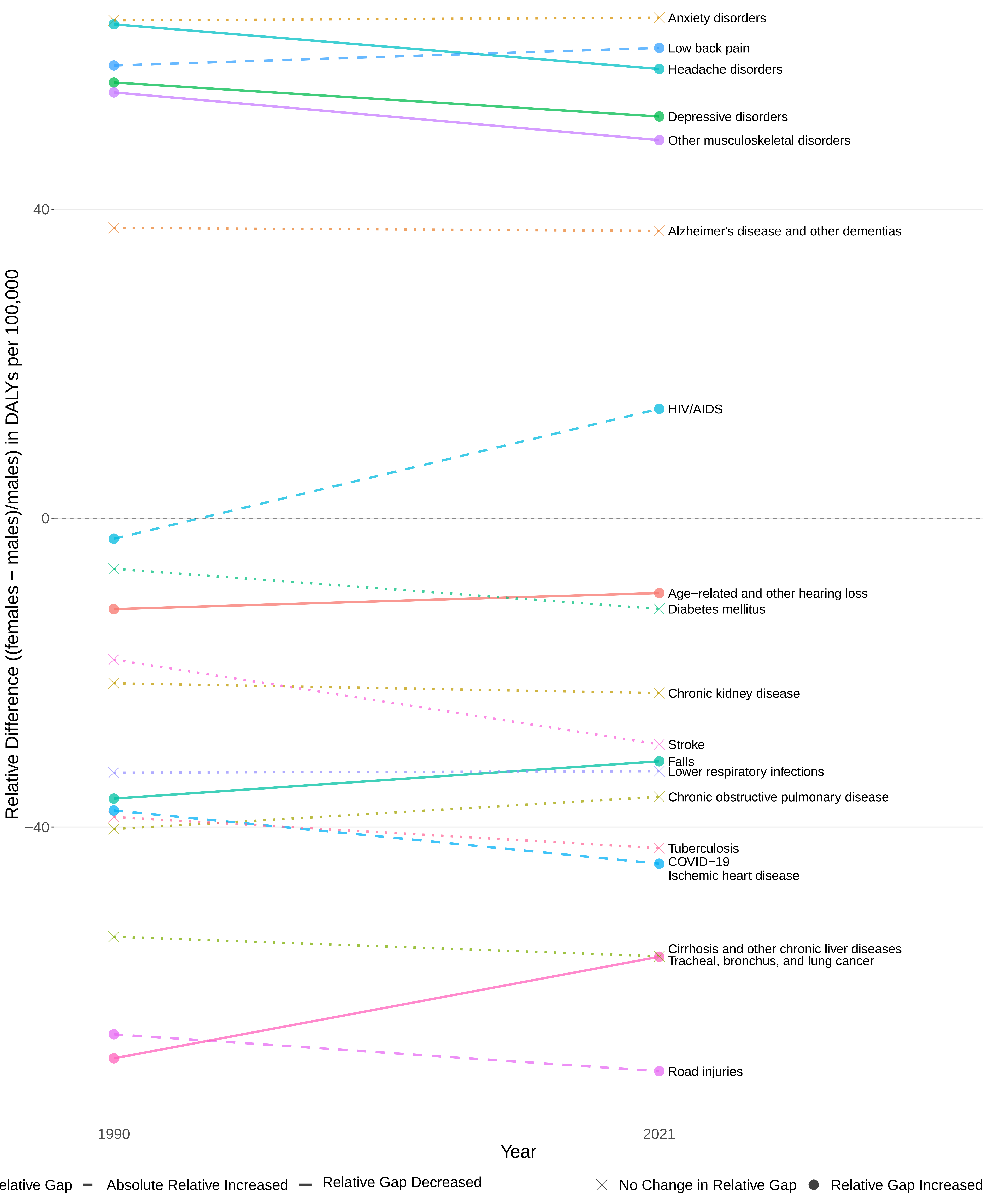

Figure S12. Temporal pattern of absolute difference between females and males in Disability-Adjusted Life Year (DALY) rates (per 100,000 population) between 1990 and 2021 in sub-Saharan Africa, age-standardised (10 years and older)

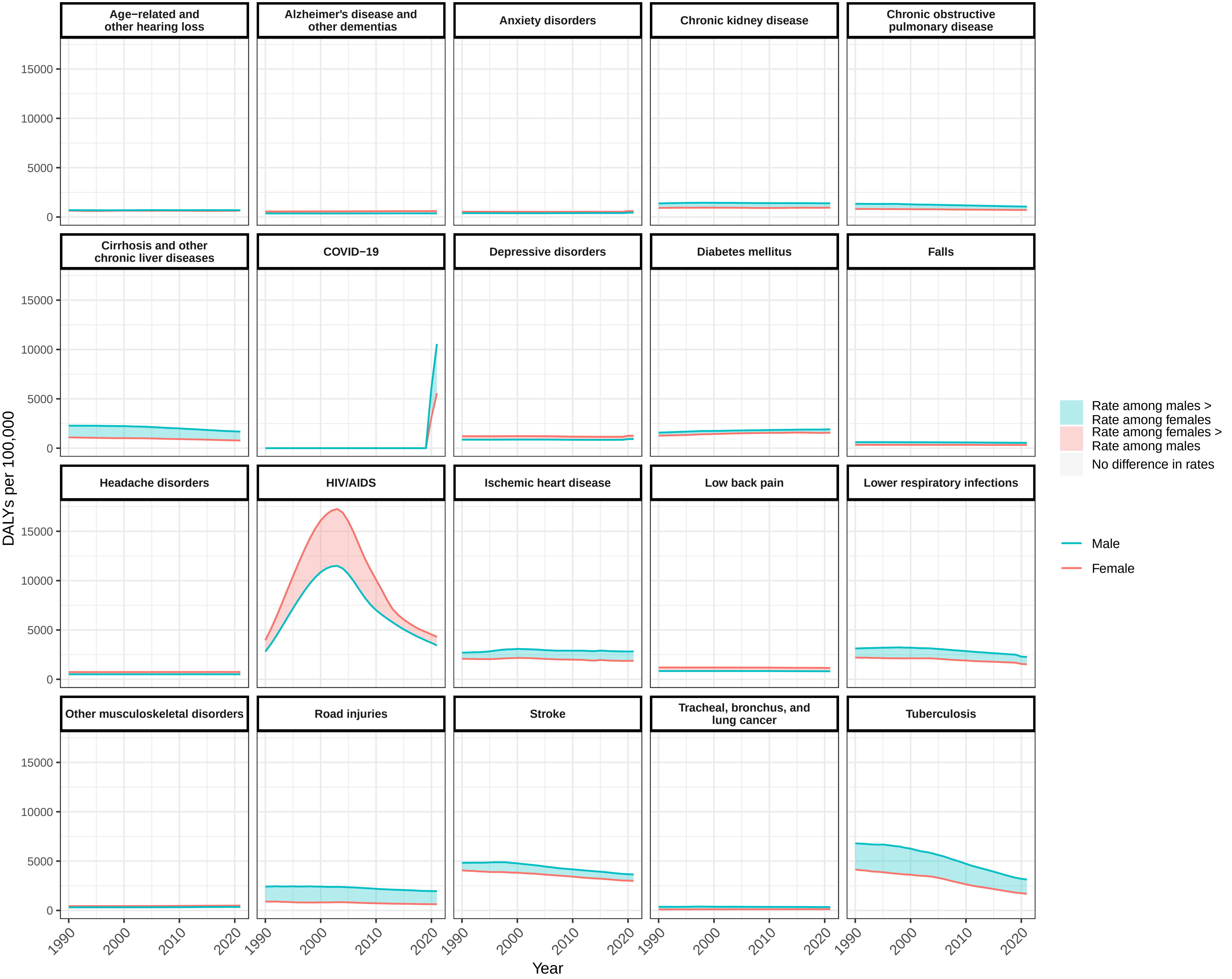

Figure S13. Temporal pattern of absolute difference between females and males in Disability-Adjusted Life Year (DALY) rates (per 100,000 population) between 1990 and 2021 in high-income countries, age-standardised (10 years and older)

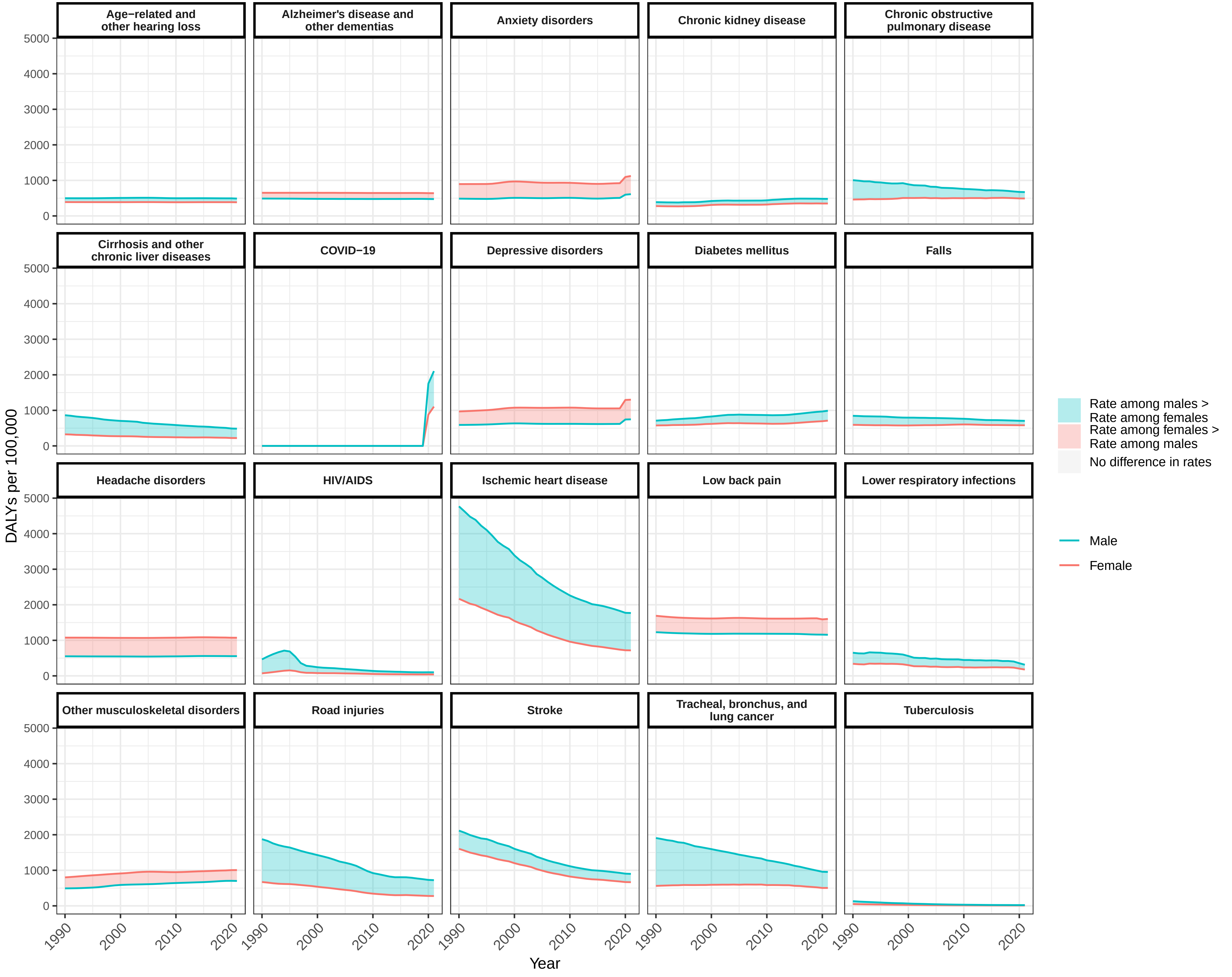

Figure S14. Temporal pattern of absolute difference between females and males in Disability-Adjusted Life Year (DALY) rates (per 100,000 population) between 1990 and 2021 in Central Europe, Eastern Europe, and Central Asia, age-standardised (10 years and older)

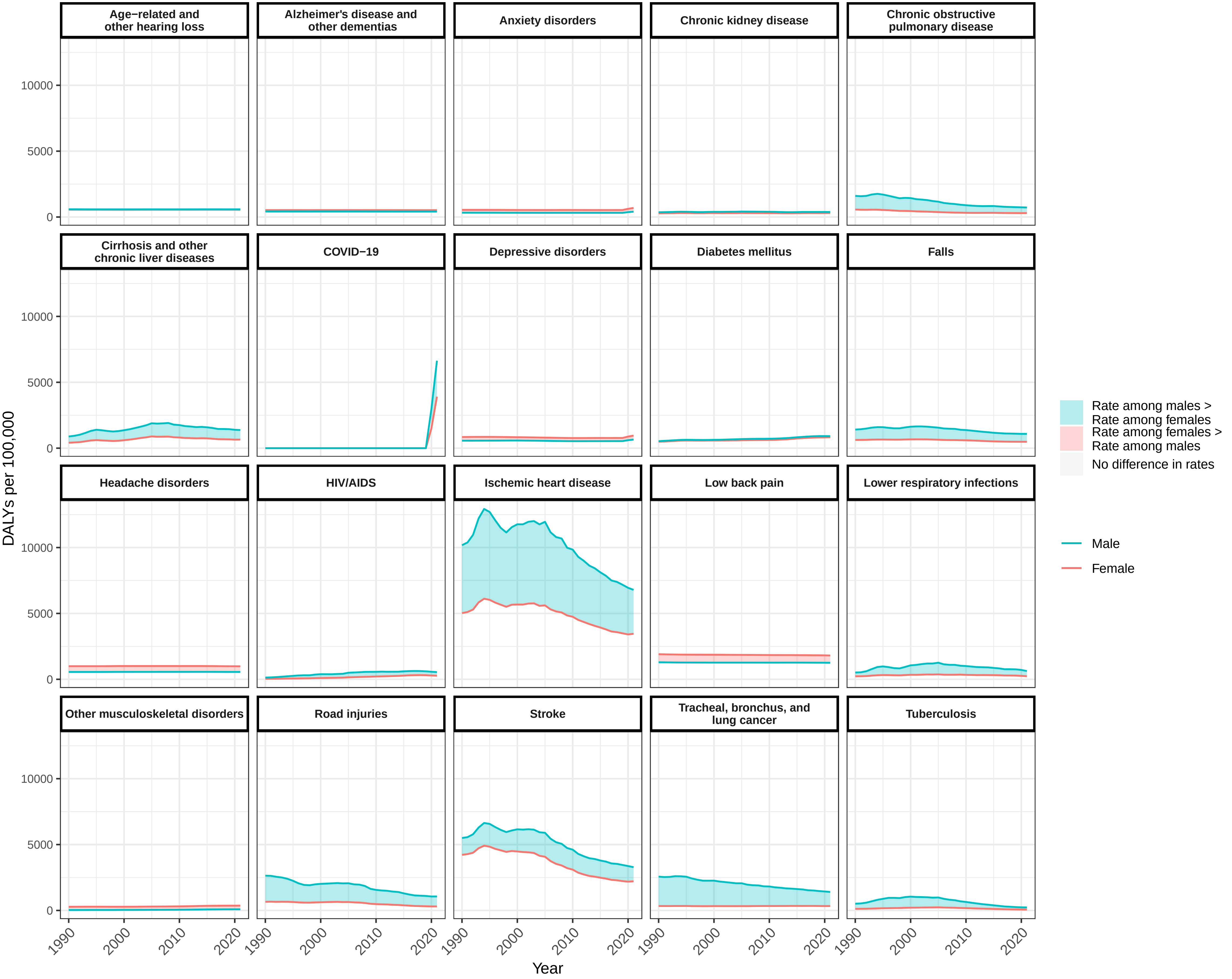

Figure S15. Temporal pattern of absolute difference between females and males in Disability-Adjusted Life Year (DALY) rates (per 100,000 population) between 1990 and 2021 in Latin America and Caribbean, age-standardised (10 years and older)

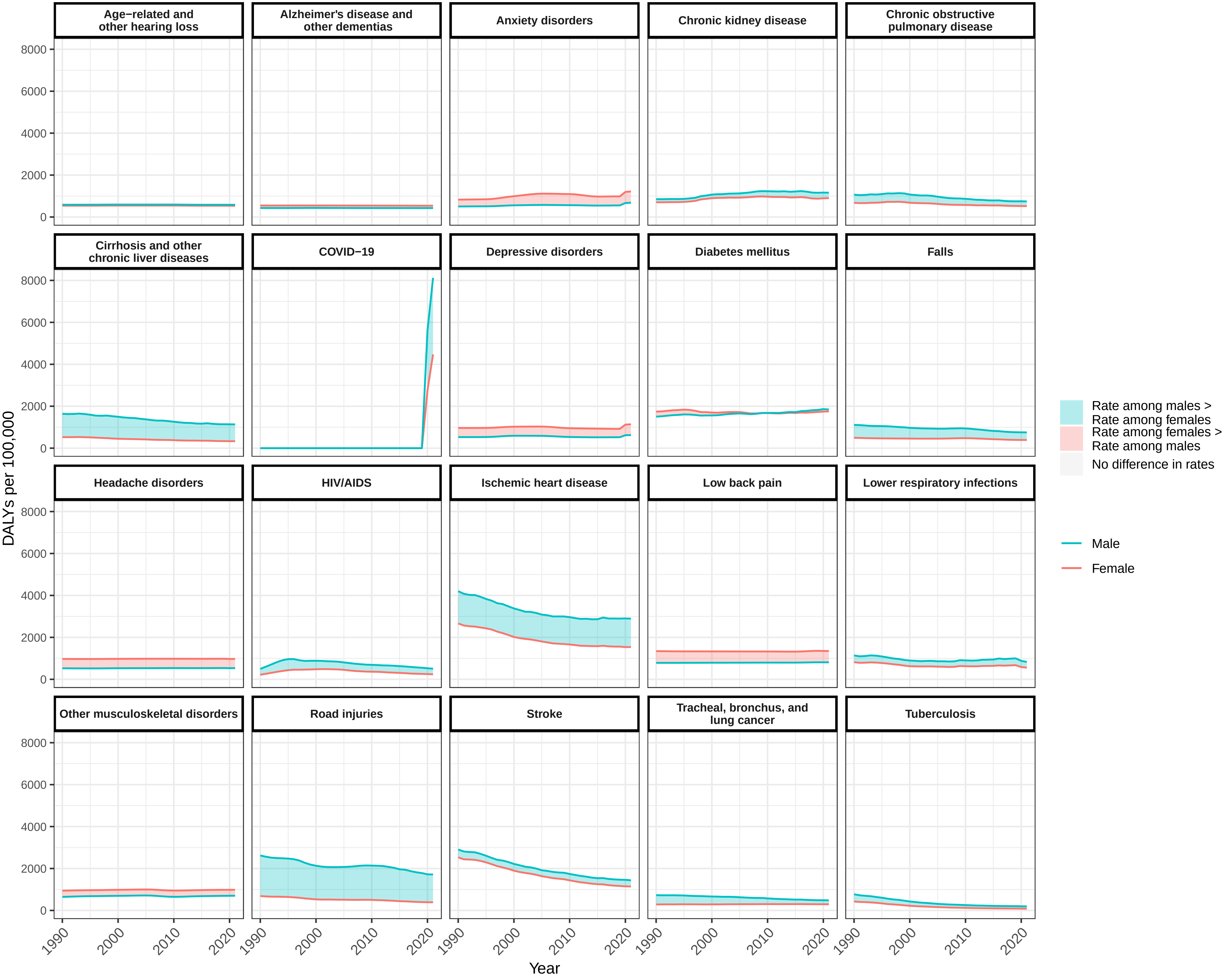

Figure S16. Temporal pattern of absolute difference between females and males in Disability-Adjusted Life Year (DALY) rates (per 100,000 population) between 1990 and 2021 in North Africa and Middle East, age-standardised (10 years and older)

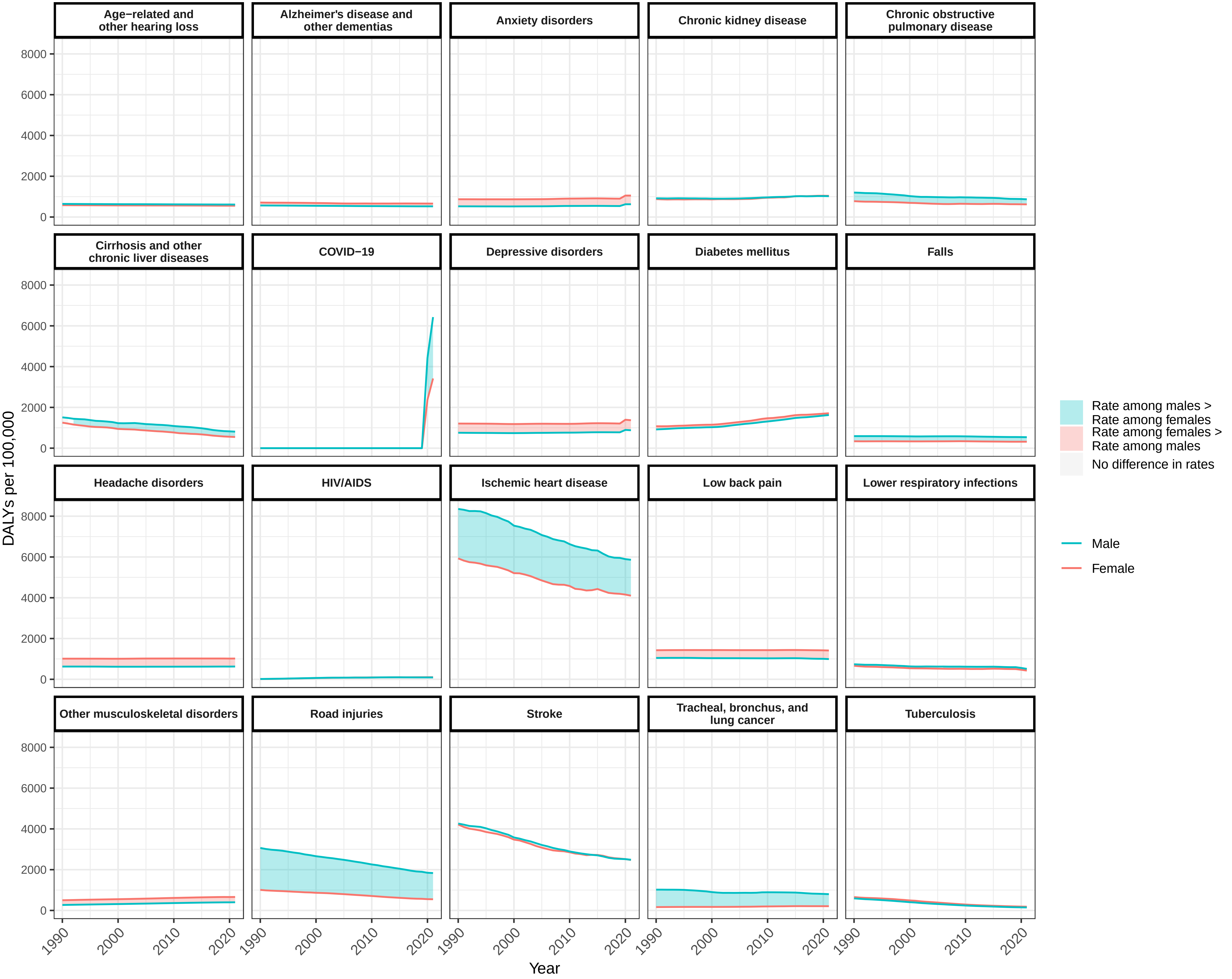

Figure S17. Temporal pattern of absolute difference between females and males in Disability-Adjusted Life Year (DALY) rates (per 100,000 population) between 1990 and 2021 in South Asia, age-standardised (10 years and older)

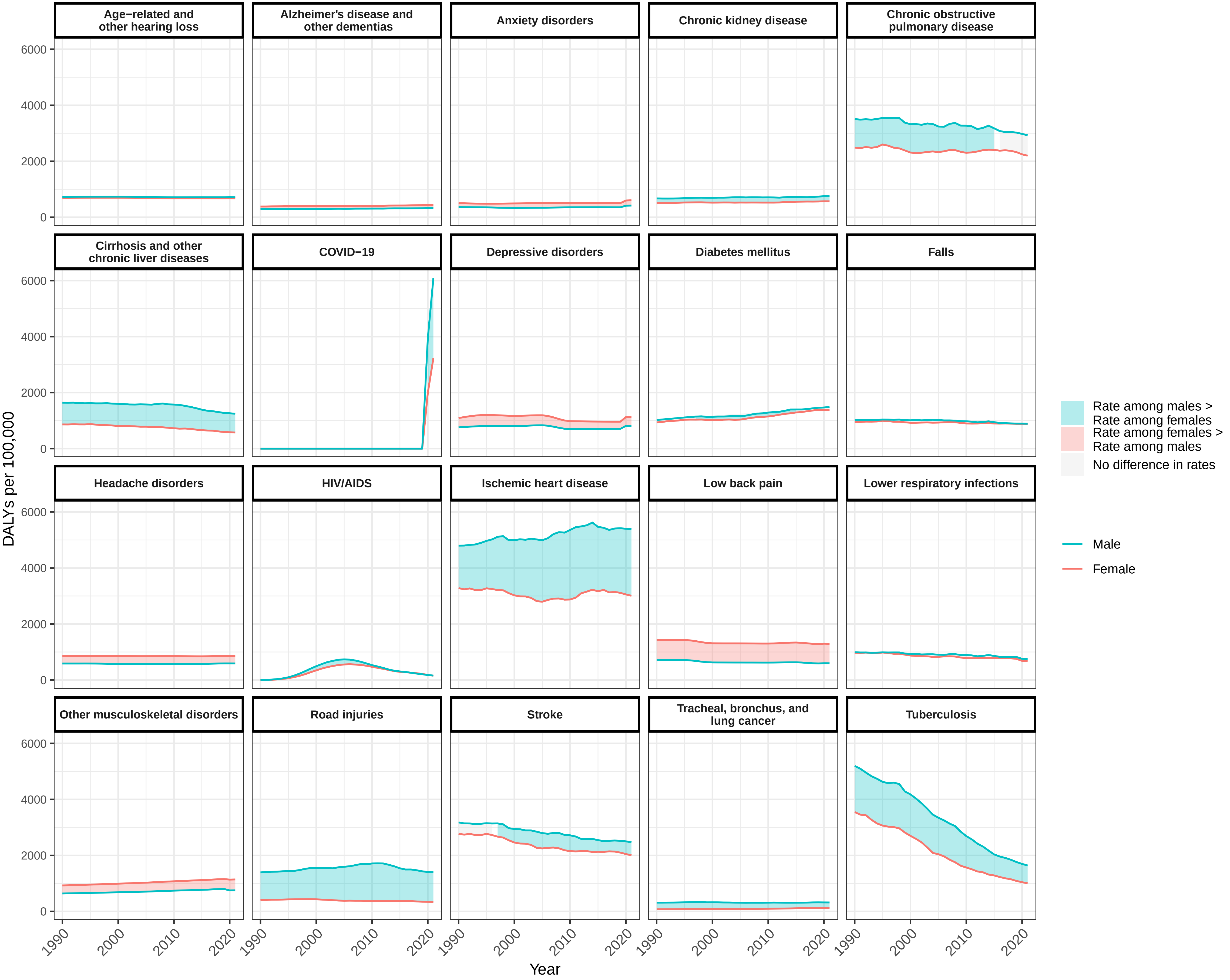

Figure S18. Temporal pattern of absolute difference between females and males in Disability-Adjusted Life Year (DALY) rates (per 100,000 population) between 1990 and 2021 in Southeast Asia, East Asia, and Oceania, age-standardised (10 years and older)

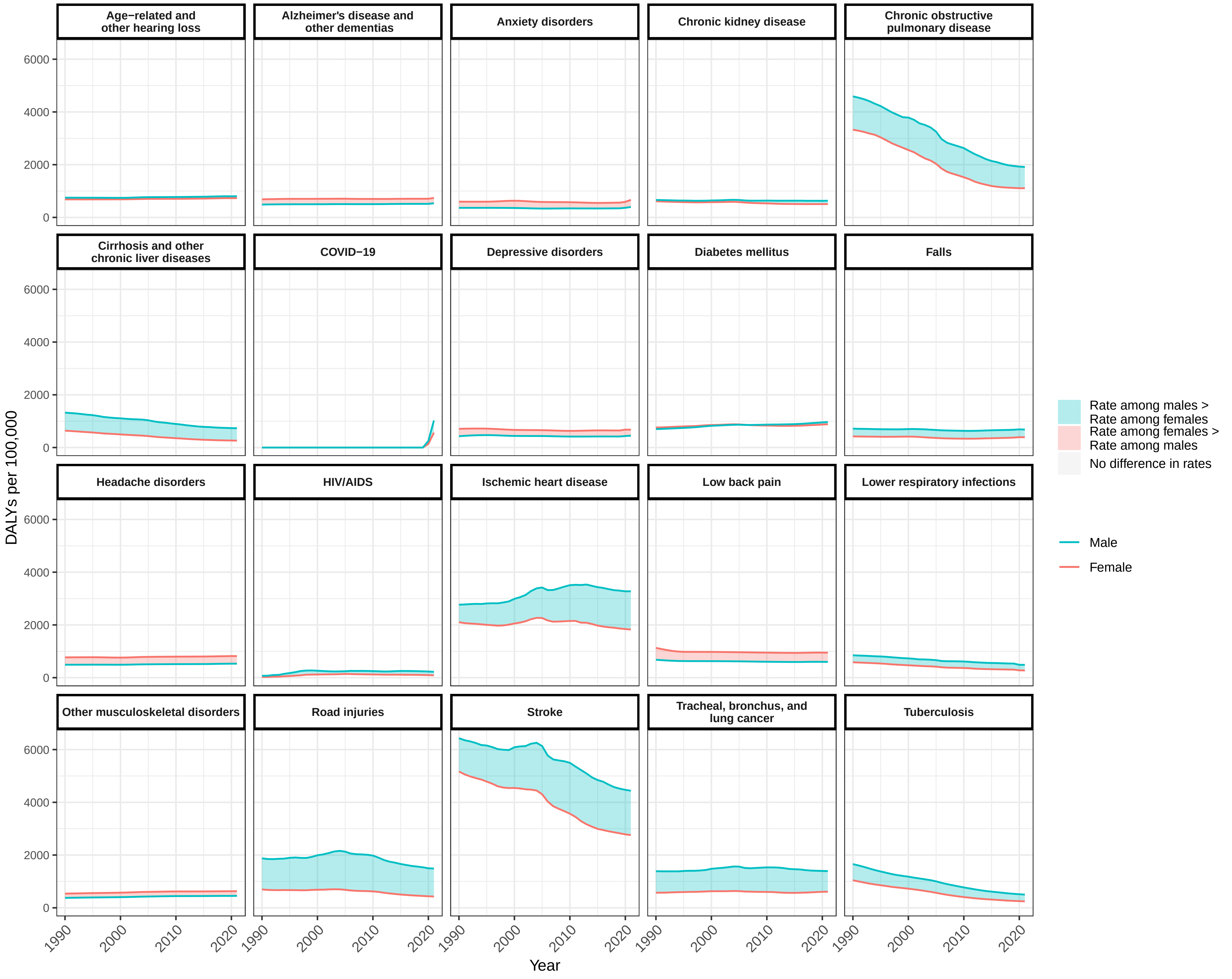

Supplement: Supplementary appendix [file mmc1.pdf]
